# Supplementary material for: Dynamin 1xA interacts with Endophilin A1 via its spliced long C-terminus for ultrafast endocytosis
Source: EMBO J. 2024 Jun 21;43(16):3327–57. doi: 10.1038/s44318-024-00145-x (PMC11329700; doi:10.1038/s44318-024-00145-x)
Supplement: Supplementary file 1 — Appendix [file 44318_2024_145_MOESM1_ESM.pdf]

Appendix Supplementary Information for

Dynamin 1xA interacts with Endophilin A1 via its spliced long C-terminus for ultrafast endocytosis

Yuuta Imoto, Jing Xue, Lin Luo, Sumana Raychaudhuri, Kie Itoh, Ye Ma, George E. Craft, Ann H. Kwan, Tyler H. Ogunmowo, Annie Ho, Joel P. Mackay, Taekjip Ha, Shigeki Watanabe, and Phillip J. Robinson

Contents

Appendix Figure S1 (Page 2)

Appendix Figure S2 (Page 4)

Appendix Figure S3 (Page 6)

Appendix Figure S4 (Page 8)

Appendix Figure S5 (Page 10)

Appendix Figure S6 (Page 12)

Appendix Figure S6 (Page 14)

Appendix Figure S1

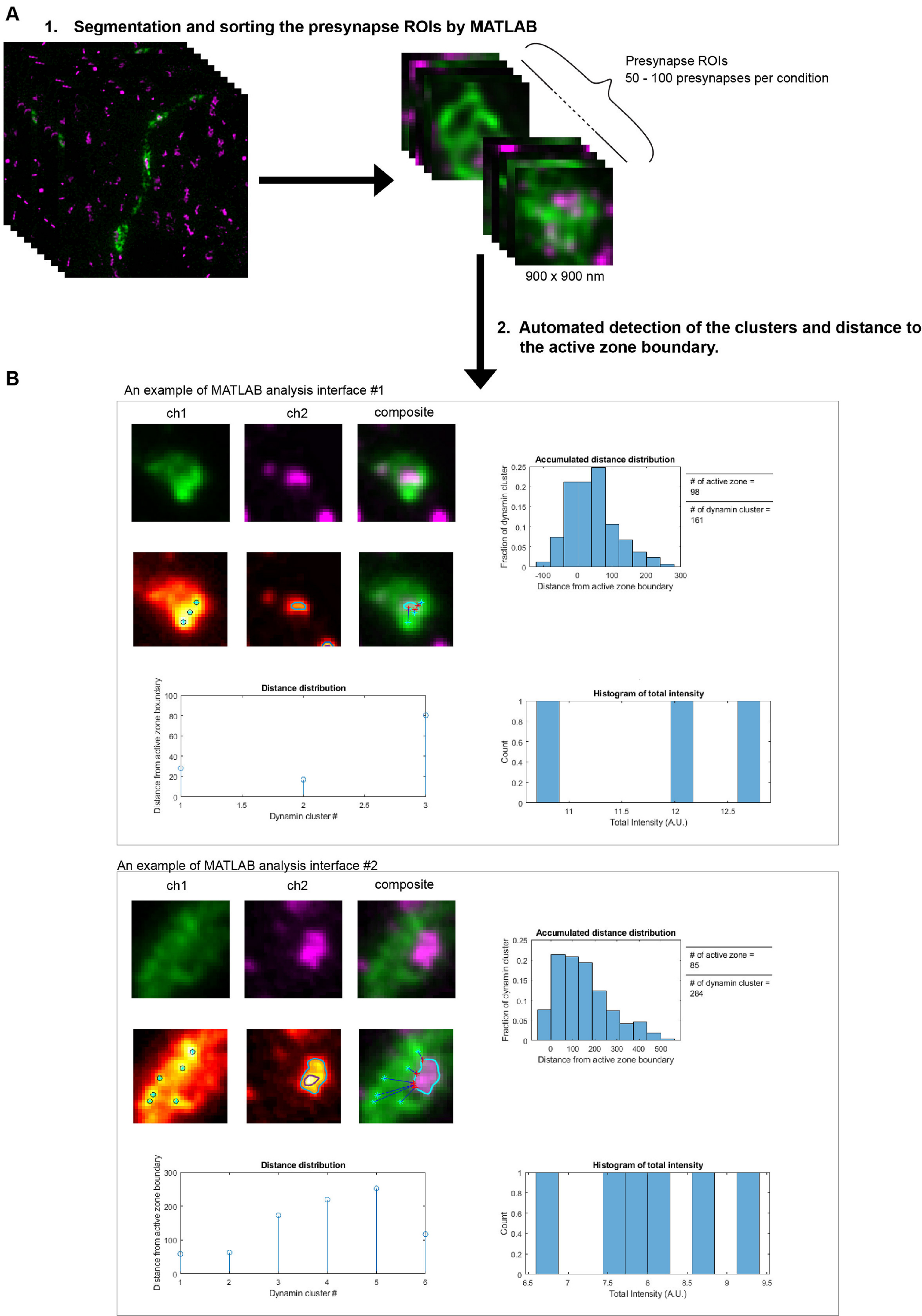

Appendix Figure S1. Workflow of the STED image analysis

Protein localization in presynapses is determined by semi-automated MATLAB script (see Method).

(A) Series of deconvoluted STED images are segmented to obtain 50-100 presynapse ROIs in each condition.

(B) Two representations of the MATLAB analysis interface are shown. The first channel (ch1, green) is processed to identify the pixels of local maxima within this channel. The second channel (ch2, magenta) is normally active zone proteins such as Bassoon. Active zone boundary is determined by the contour generated at 50% intensity of the local maxima of ch2. The contours outside of the transfected neurons are manually selected on the interface and excluded from the analysis. Minimum distances from each the pixels of local maxima in ch1 to the contour in ch2 are calculated and shown in the composite image. The plot "Distance distribution" shows all the minimum distance identified in this presynapses ROI (unit of the y axis is nanometer). The plot "Accumulated distance distribution" shows the accumulated distance distribution from the initial to the current presynapses ROI. The plot "Histogram of total intensity" shows the intensity counts around individual local maxima pixels in ch1.

Appendix Figure S2

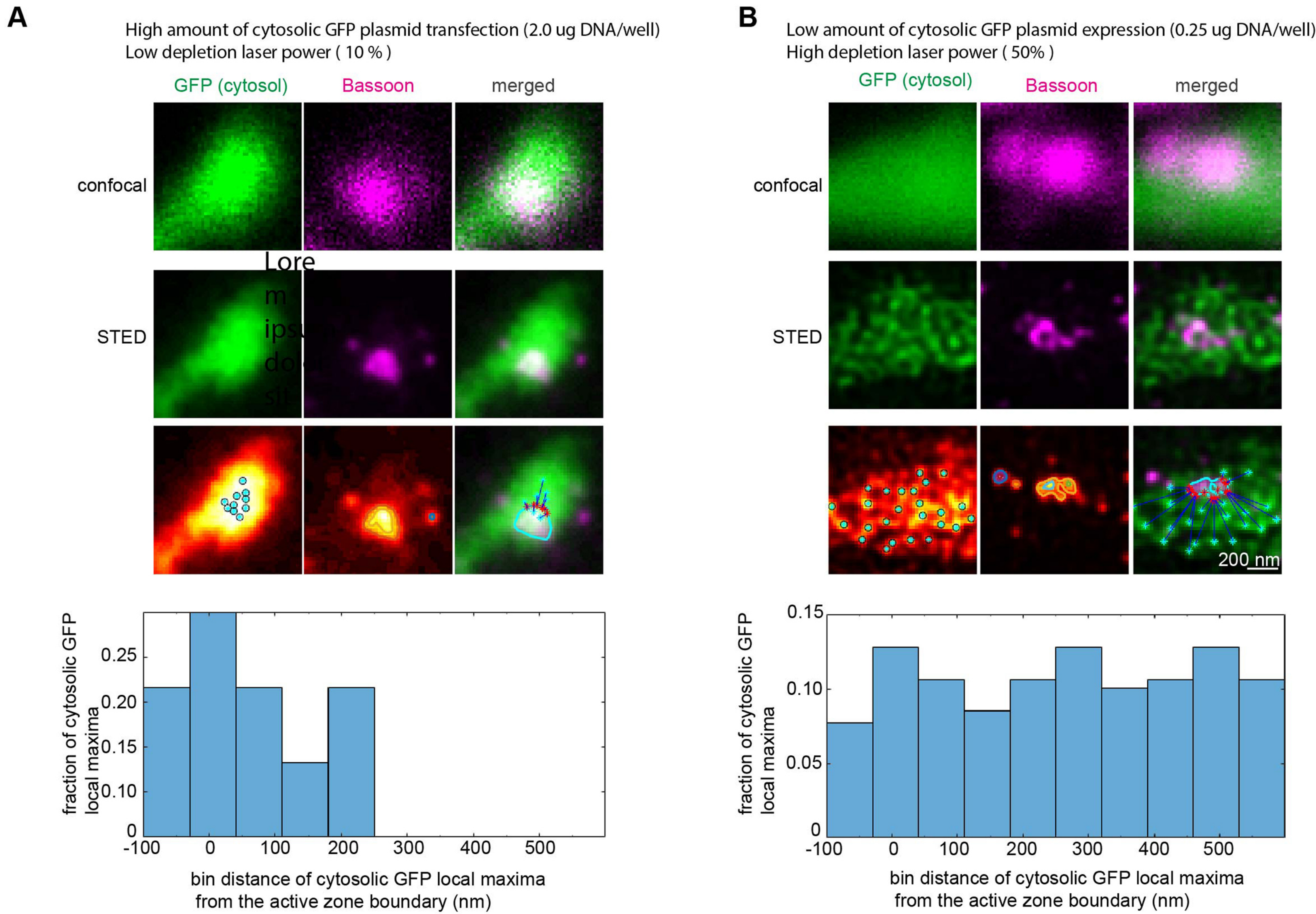

Appendix Figure S2. Different plasmid expression levels and the depletion laser power affect cluster analysis in STED images.

(A) STED images show high expression of cytosolic GFP scanned by low depletion laser power. Anti-Bassoon antibody is used as presynaptic marker. Bottom panel shows distance of misdirected GFP clusters from active zone edge.

(B) STED images show low expression of cytosolic GFP scanned by sufficient depletion laser power. Anti-Bassoon antibody is used as presynaptic marker. Bottom panel shows distance of GFP clusters from active zone edge. By using appropriate amount of plasmid transfection and the depletion laser power, diffuse cytosolic GFP clusters are detected.

Appendix Figure S3

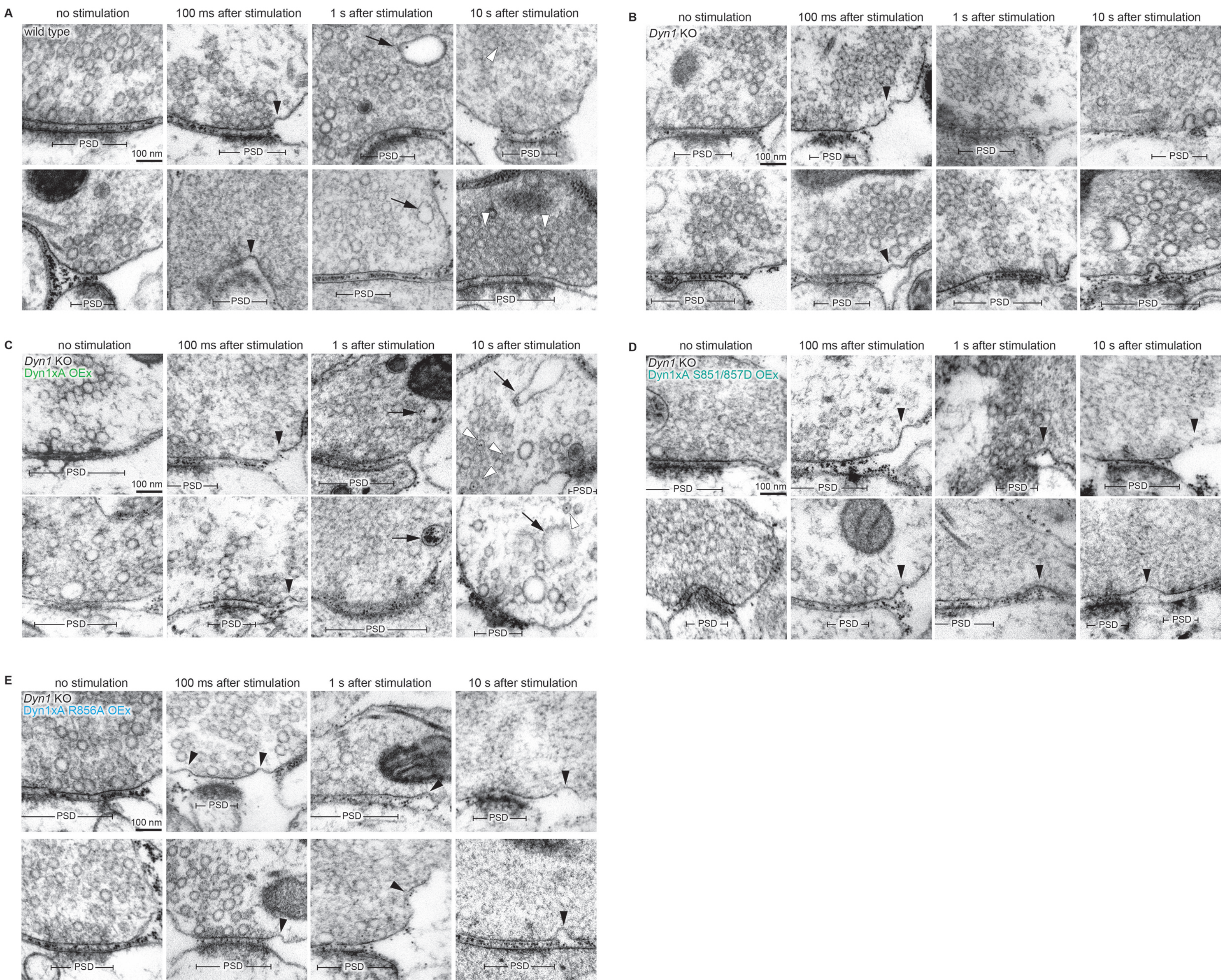

Appendix Figure S3. Additional EM images for Figure 5

Example micrographs showing endocytic pits and ferritin-containing endocytic structures at the indicated time points in wild-type neurons, *Dyn1* KO neurons, and *Dyn1* KO neurons overexpressing Dyn1xA (Dyn1xA OEx), Dyn1xA S851/857D (Dyn1xA S851/857D OEx) and Dyn1xA R846A (Dyn1xA R846A OEx). Scale bar: 100 nm.

Appendix Figure S4

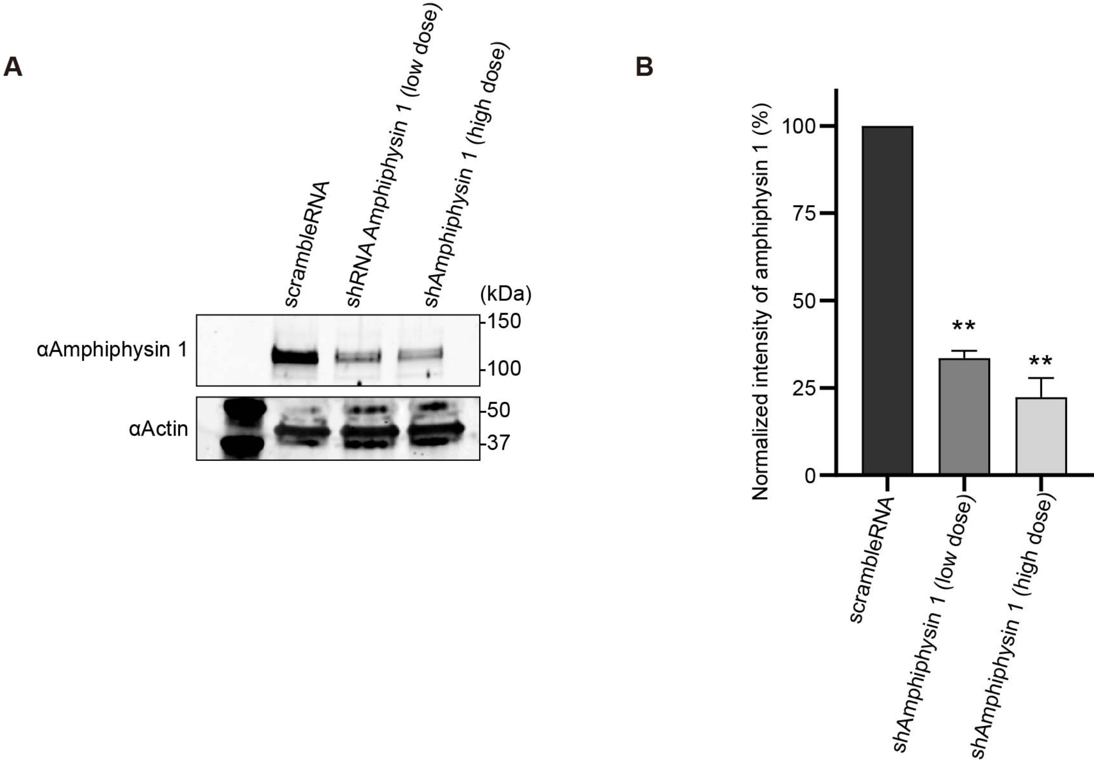

Appendix Figure S4. Evaluation of Amphiphysin 1 knock down

(A) An example immunoblotting images showing anti-Amphiphysin 1 or anti-β-Actin antibodies reactions against the lysates of cultured hippocampal neurons infected with lentivirus expressing scramble shRNA and two different doses of Amphiphysin 1 shRNA (see Material and Method for detail).

(B) Normalized signal intensities of Amphiphysin 1 quantified from two independent immunoblots.

*$p < 0.05$, unpaired t test. The mean and SEM are shown. n = 2 independent cultures.

Appendix Figure S5

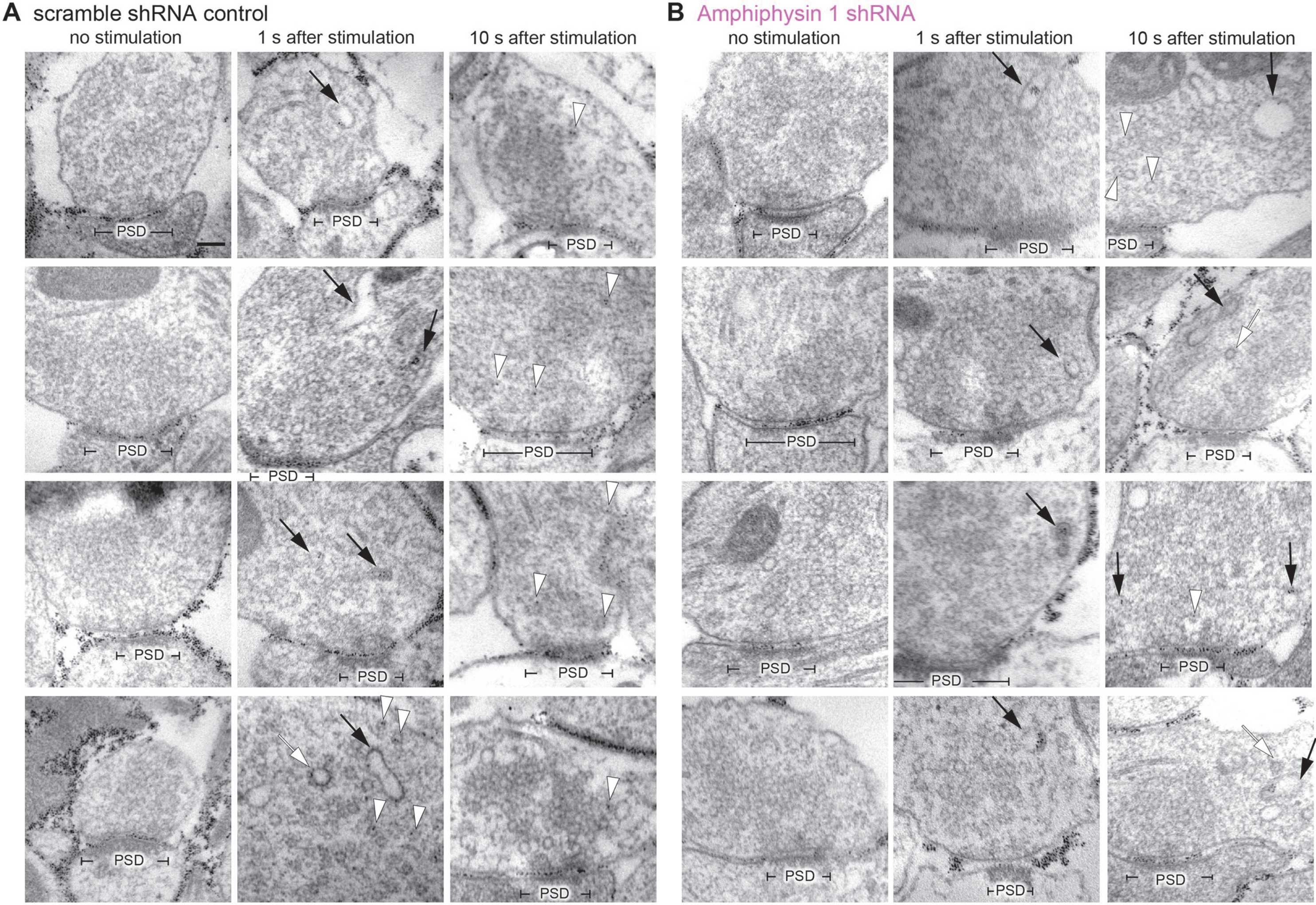

Appendix Figure S5. Additional EM images for Figure EV5

Example micrographs showing endocytic pits and ferritin-containing endocytic structures at the indicated time points in neurons expressing scramble RNA (A) and Amphiphysin 1 shRNA (C). Black arrows, ferritin-positive large endocytic vesicles (LEVs) or endosomes; white arrow, clathrin-coated vesicle; white arrowheads, ferritin-positive synaptic vesicles. Scale bar: 100 nm. PSD, post-synaptic density.

Appendix Figure S6

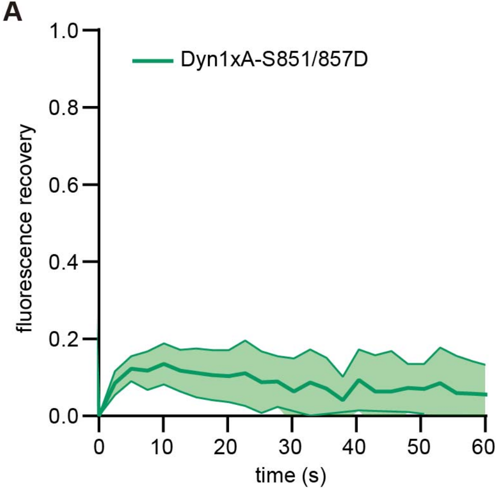

Appendix Figure S6. Dyn1xA-S851/857-GFP puncta are dominated by immobile fraction

(A) Examples live images of FRAP experiments of Dyn1xA-S851/857-GFP puncta in presynapse. mCherry-synaptobrevin 2 (mCherry-Syb2) was tandemly expressed to find presynapses. Dyn1xA-S851/857-GFP signals were photobleached at 480 nm. Time indicates after the photobleaching.

(B) Normalized fluorescence recovery of Dyn1xA-S851/857-GFP signals. Fluorescence signals were normalized to just after (0 s) photobleaching. Times indicate after the photobleaching. The median and 95% confidential interval are shown.

n = 18 presynapses from two independent cultures.

Appendix Figure S7

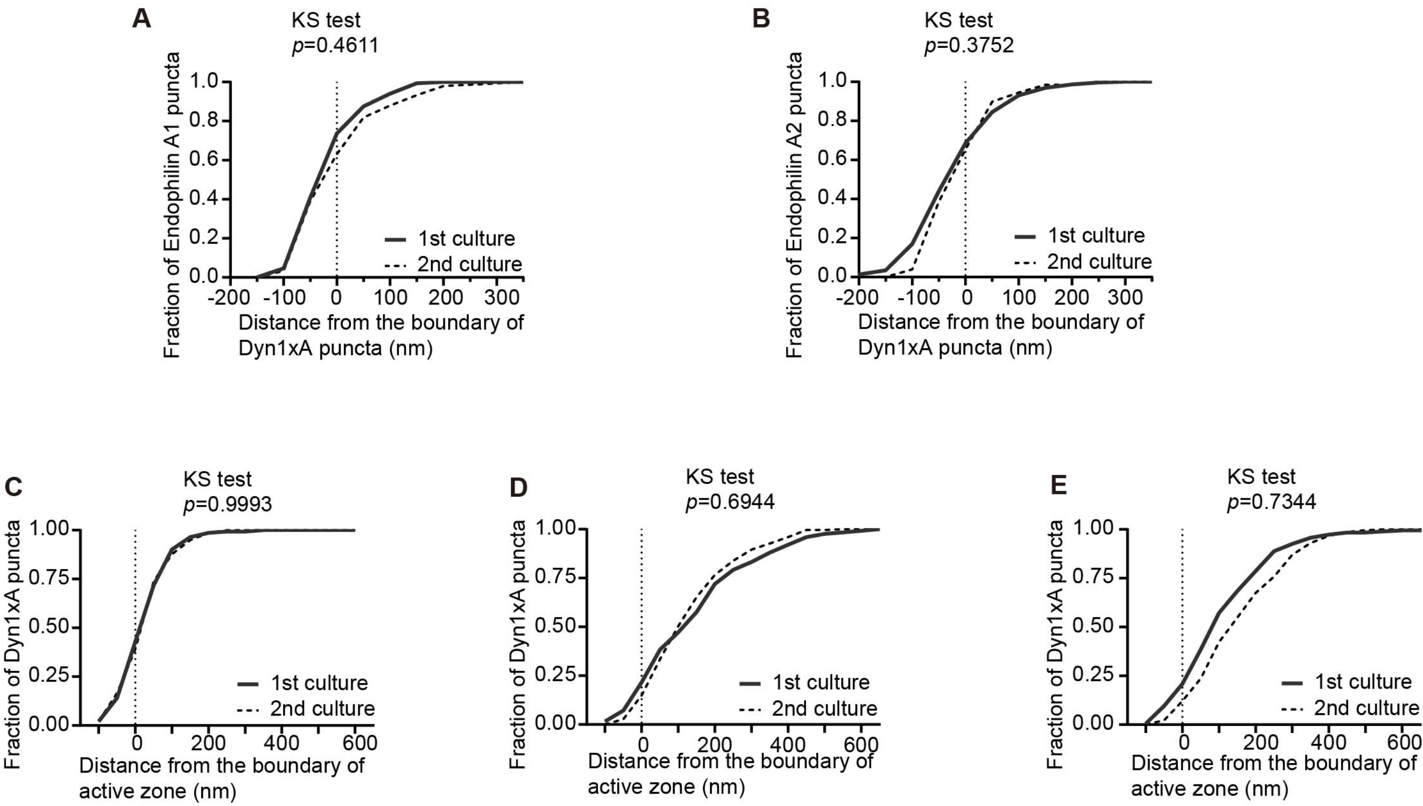

Appendix Figure S7. Data variability in Figure 4

Cumulative curves are made from each dataset of (A) distance of Endophilin A1 puncta from the edge of Dyn1xA puncta, (B) distance of Endophilin A2 puncta from the edge of Dyn1xA puncta, distance distribution of Dyn1xA from the active zone edge in (C) neurons expressing wild-type Dyn1xA-GFP, (D) Dyn1xA-S851/857-GFP and (E) Dyn1xA-R846-GFP. n > 4 coverslips from 2 independent cultures. Kolmogorov–Smirnov (KS) test, *p* values are indicated in each plot.
