# Supplementary figures and images for: Dynamin 1xA interacts with Endophilin A1 via its spliced long C-terminus for ultrafast endocytosis (part 1 of 2)
Source: EMBO J. 2024 Jun 21;43(16):3327–57. doi: 10.1038/s44318-024-00145-x (PMC11329700; doi:10.1038/s44318-024-00145-x)

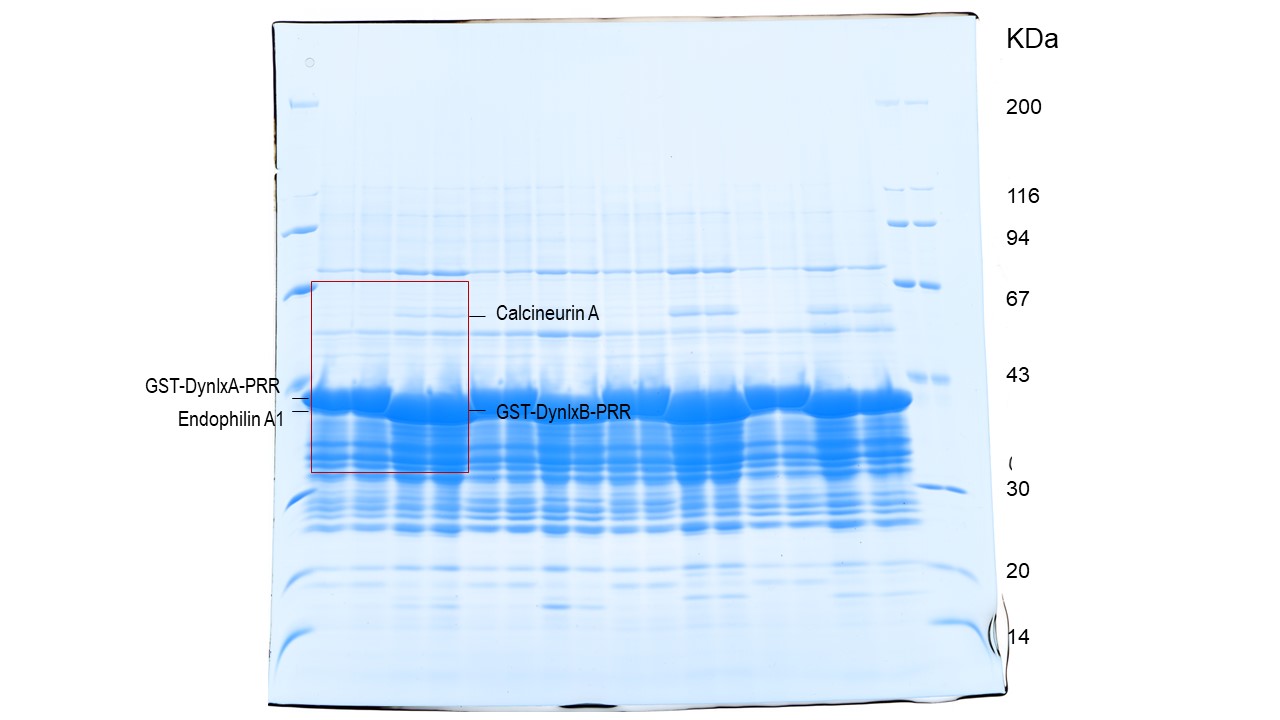

Supplement: Supplementary file 3 — Source data Fig. 1 [file 44318_2024_145_MOESM3_ESM.zip › Source_data_Figure_1/1B/1B.jpg]

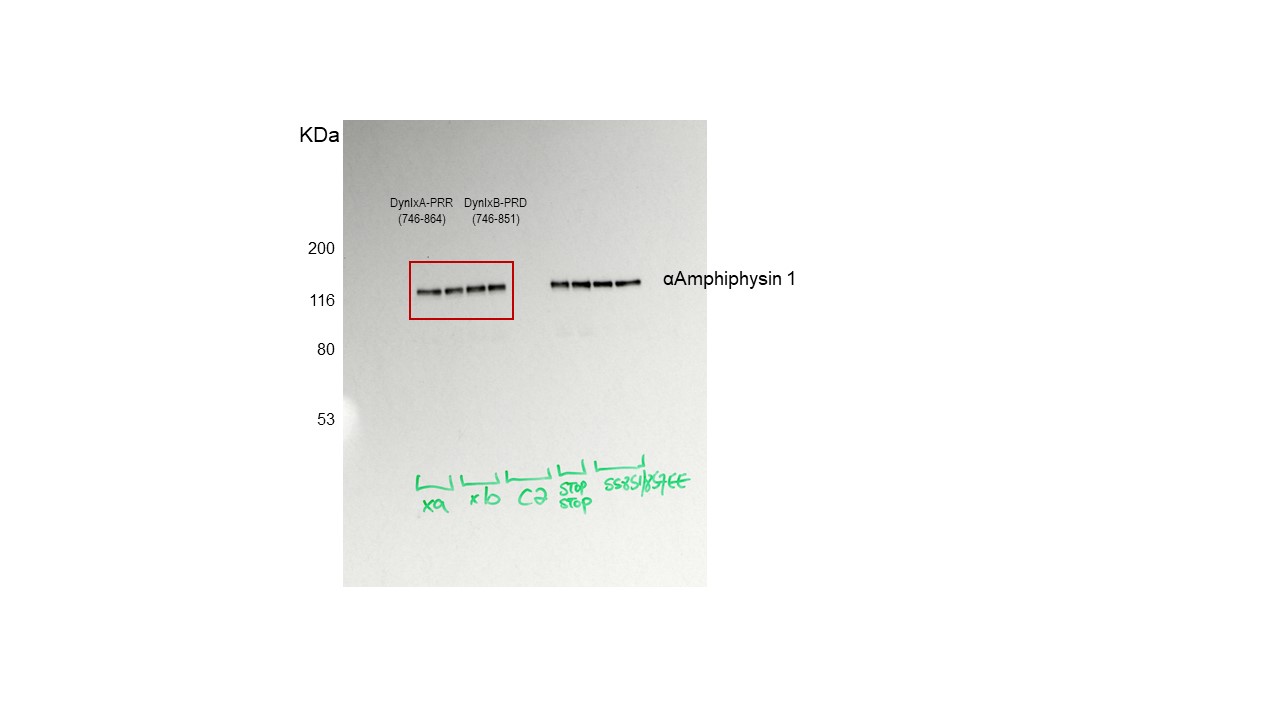

Supplement: Supplementary file 3 — Source data Fig. 1 [file 44318_2024_145_MOESM3_ESM.zip › Source_data_Figure_1/1C/Western Amphiphysin.jpg]

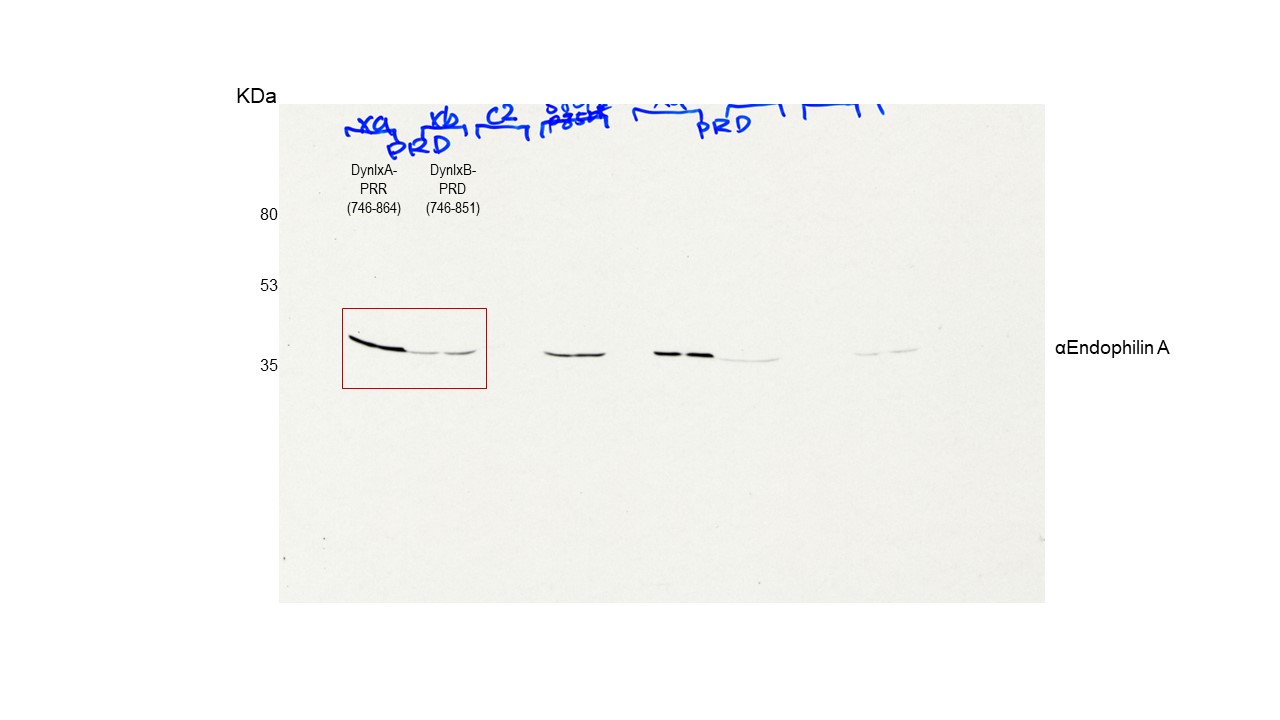

Supplement: Supplementary file 3 — Source data Fig. 1 [file 44318_2024_145_MOESM3_ESM.zip › Source_data_Figure_1/1C/Western Endophilin.jpg]

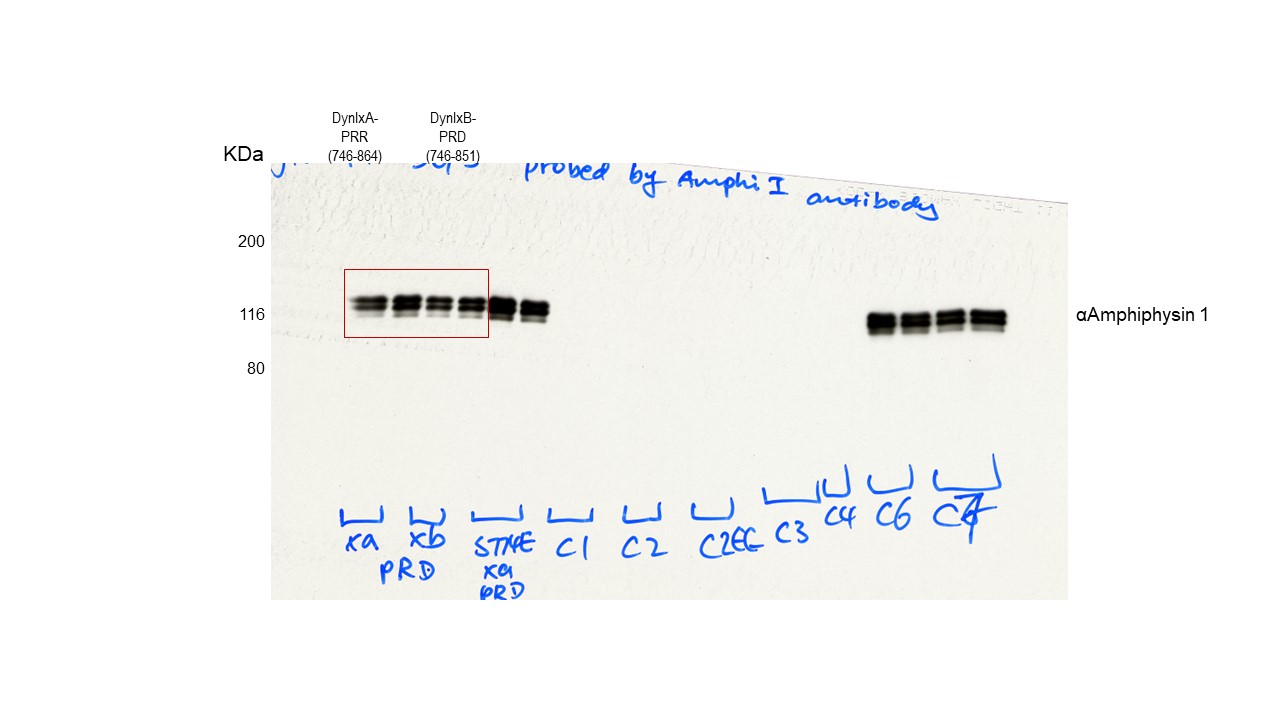

Supplement: Supplementary file 3 — Source data Fig. 1 [file 44318_2024_145_MOESM3_ESM.zip › Source_data_Figure_1/1D/Western Amphiphysin.jpg]

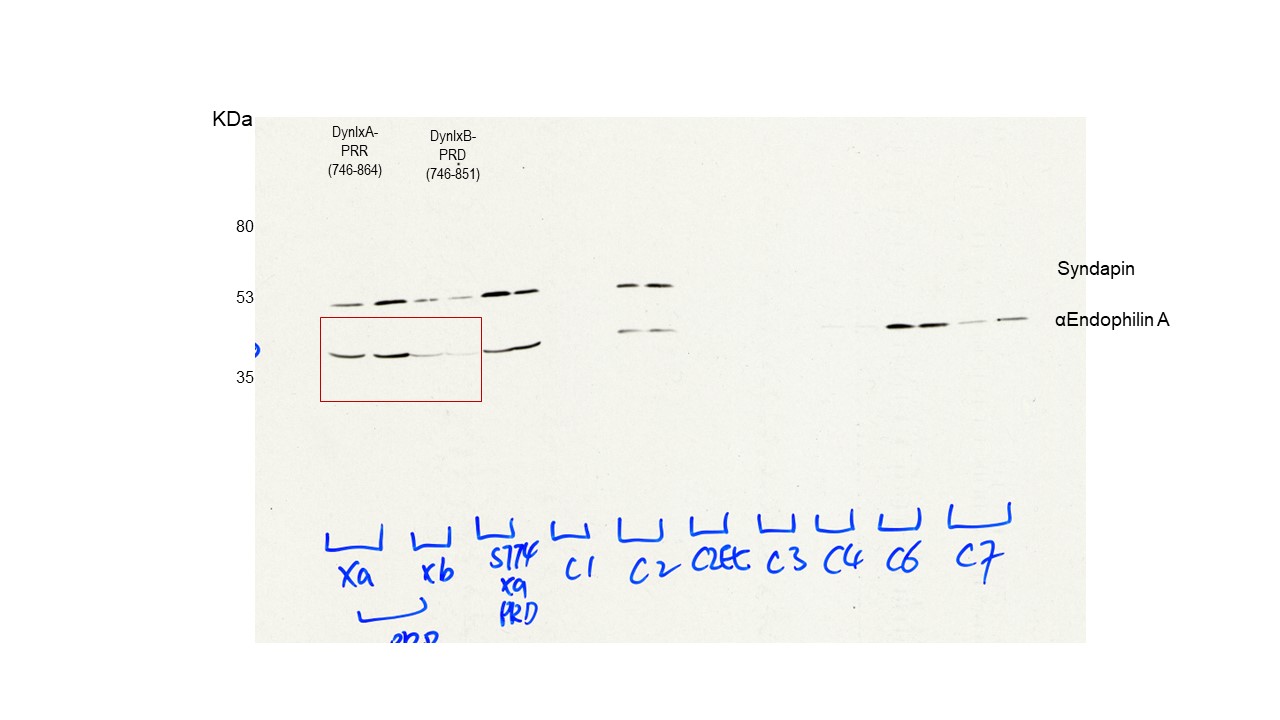

Supplement: Supplementary file 3 — Source data Fig. 1 [file 44318_2024_145_MOESM3_ESM.zip › Source_data_Figure_1/1D/Western Endophilin.jpg]

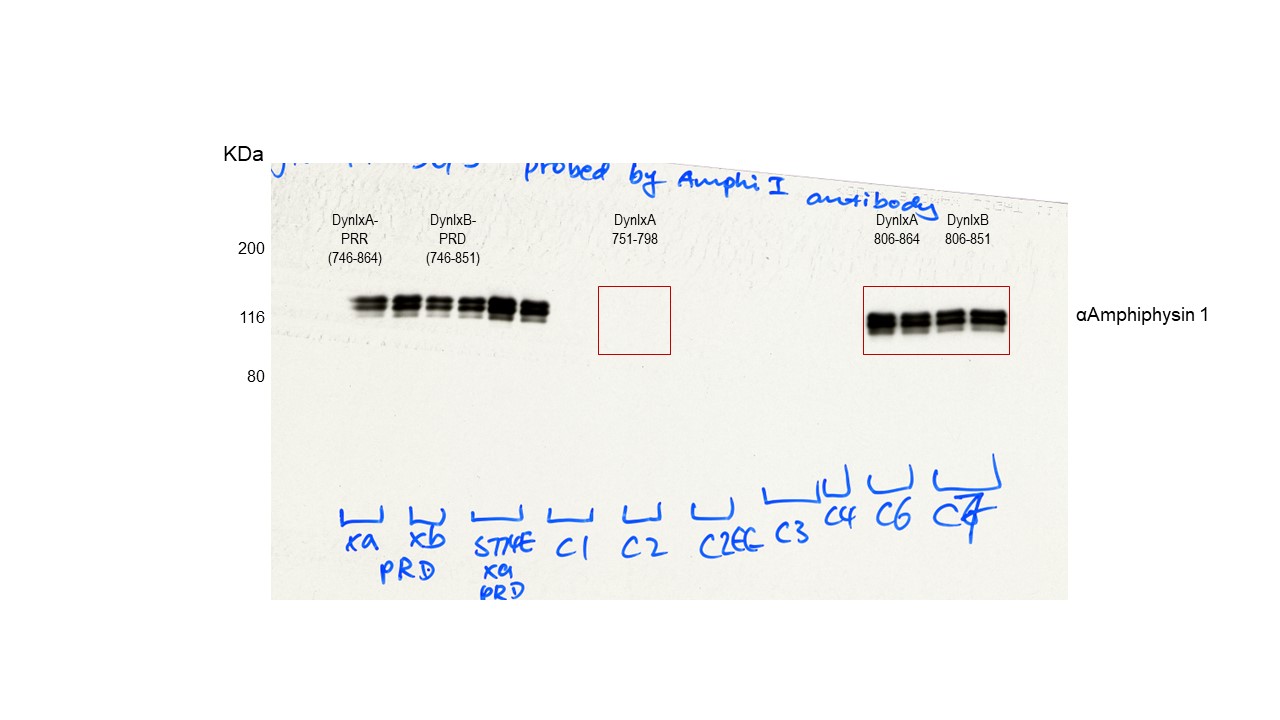

Supplement: Supplementary file 3 — Source data Fig. 1 [file 44318_2024_145_MOESM3_ESM.zip › Source_data_Figure_1/1E/Western Amphiphysin.jpg]

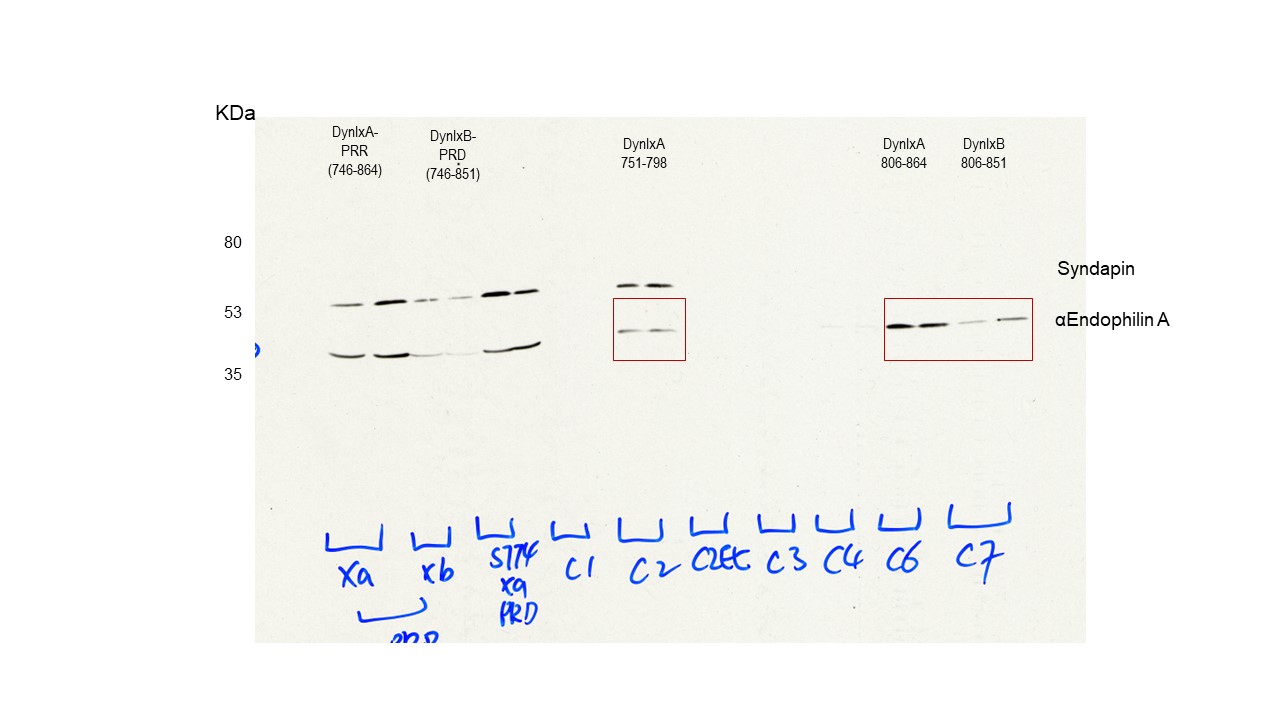

Supplement: Supplementary file 3 — Source data Fig. 1 [file 44318_2024_145_MOESM3_ESM.zip › Source_data_Figure_1/1E/Western Endophilin.jpg]

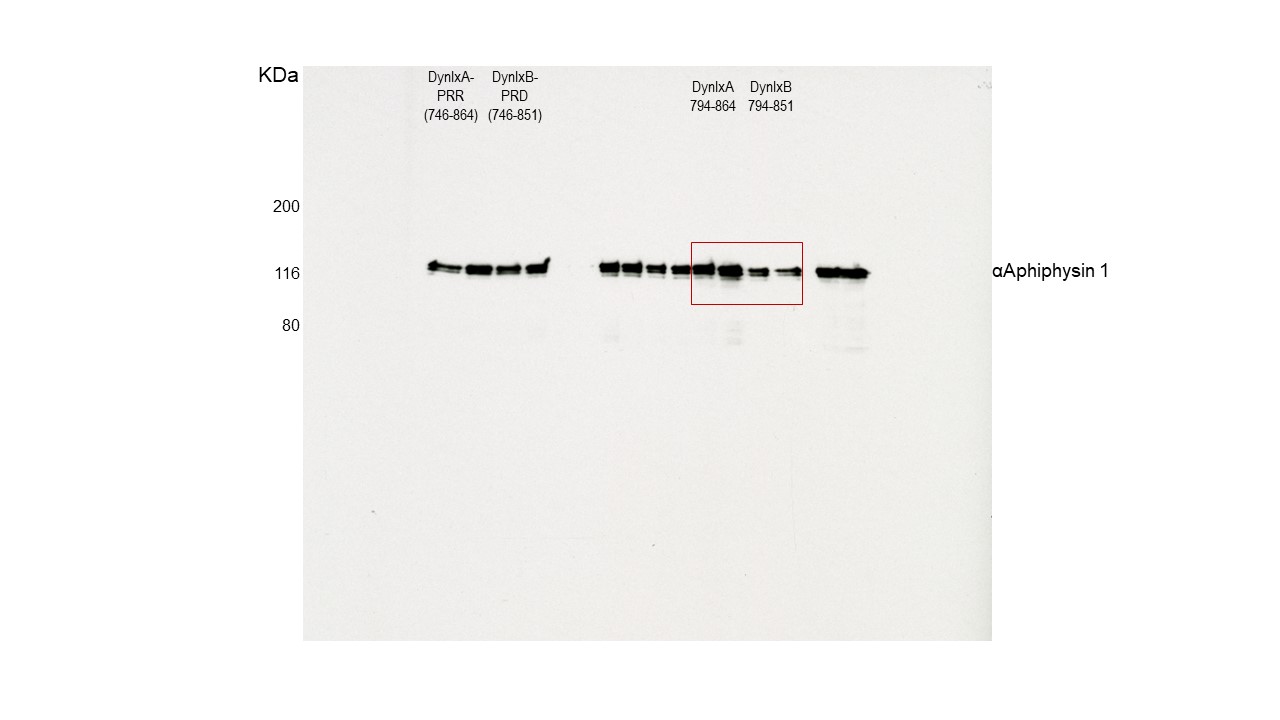

Supplement: Supplementary file 3 — Source data Fig. 1 [file 44318_2024_145_MOESM3_ESM.zip › Source_data_Figure_1/1F/Western Amphiphysin.jpg]

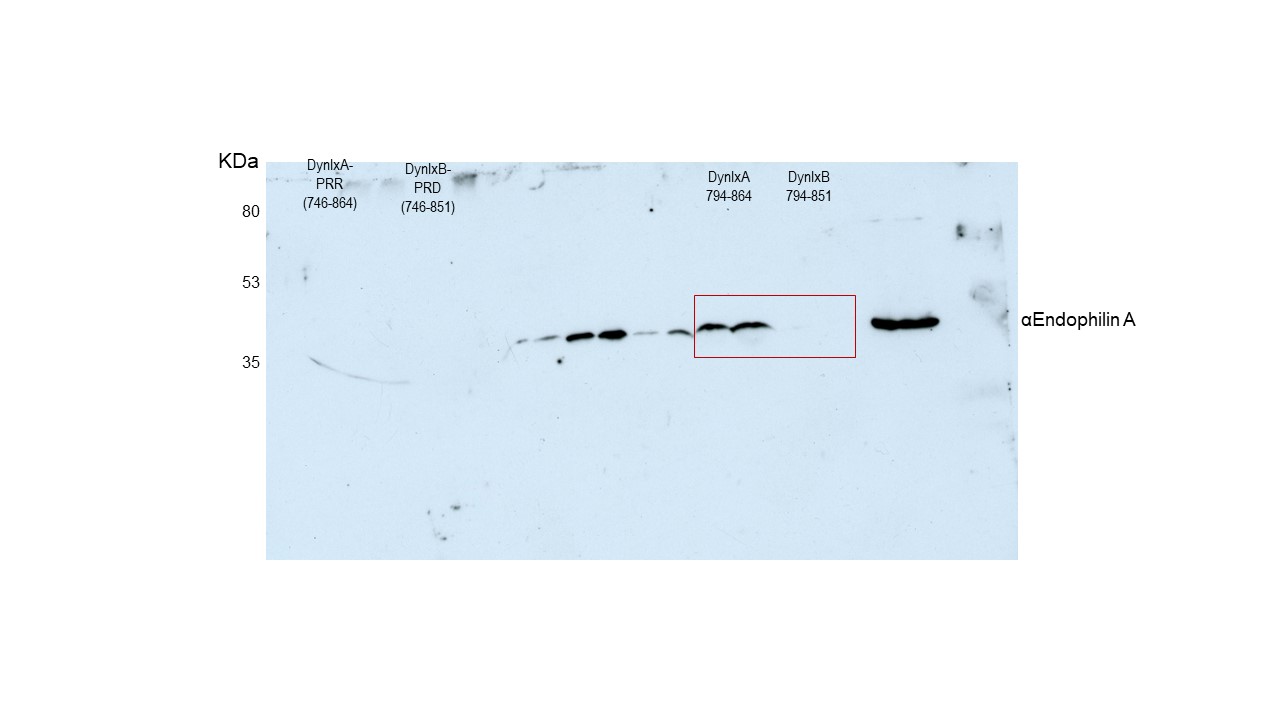

Supplement: Supplementary file 3 — Source data Fig. 1 [file 44318_2024_145_MOESM3_ESM.zip › Source_data_Figure_1/1F/Western Endophilin.jpg]

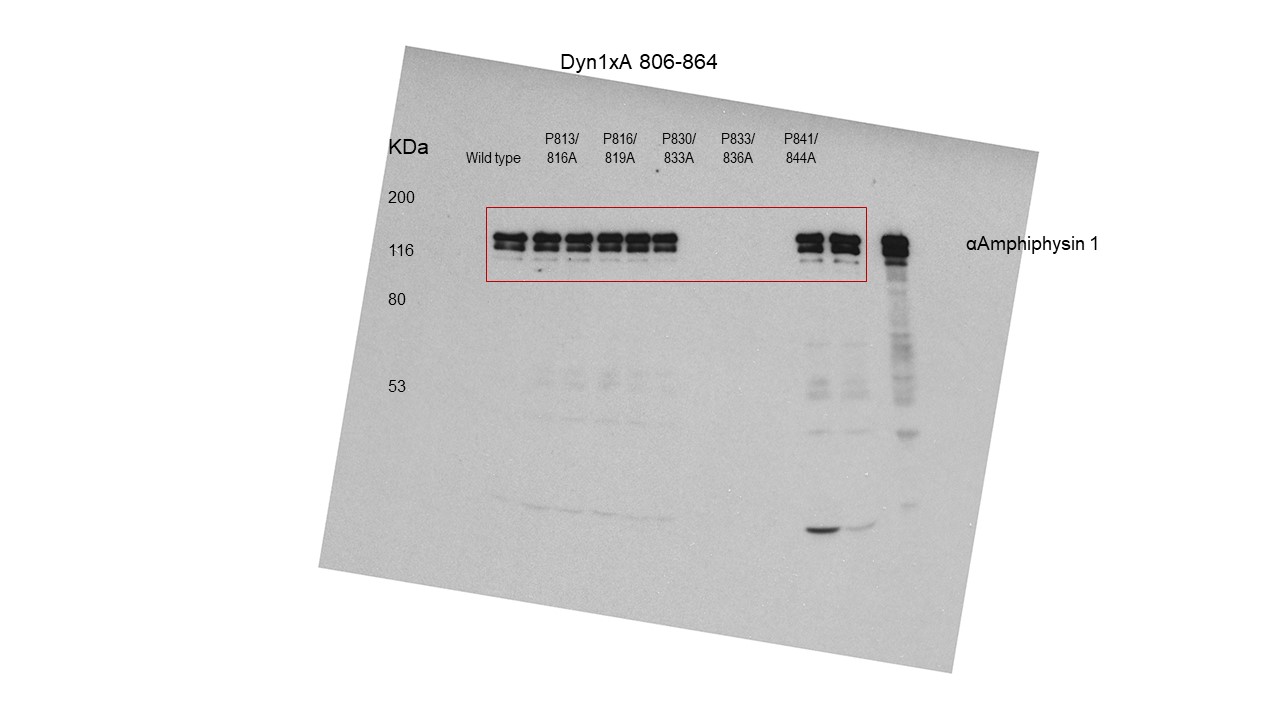

Supplement: Supplementary file 5 — Source data Fig. 3 [file 44318_2024_145_MOESM5_ESM.zip › Source_data_Figure_3/3B/Western Amphiphysin.jpg]

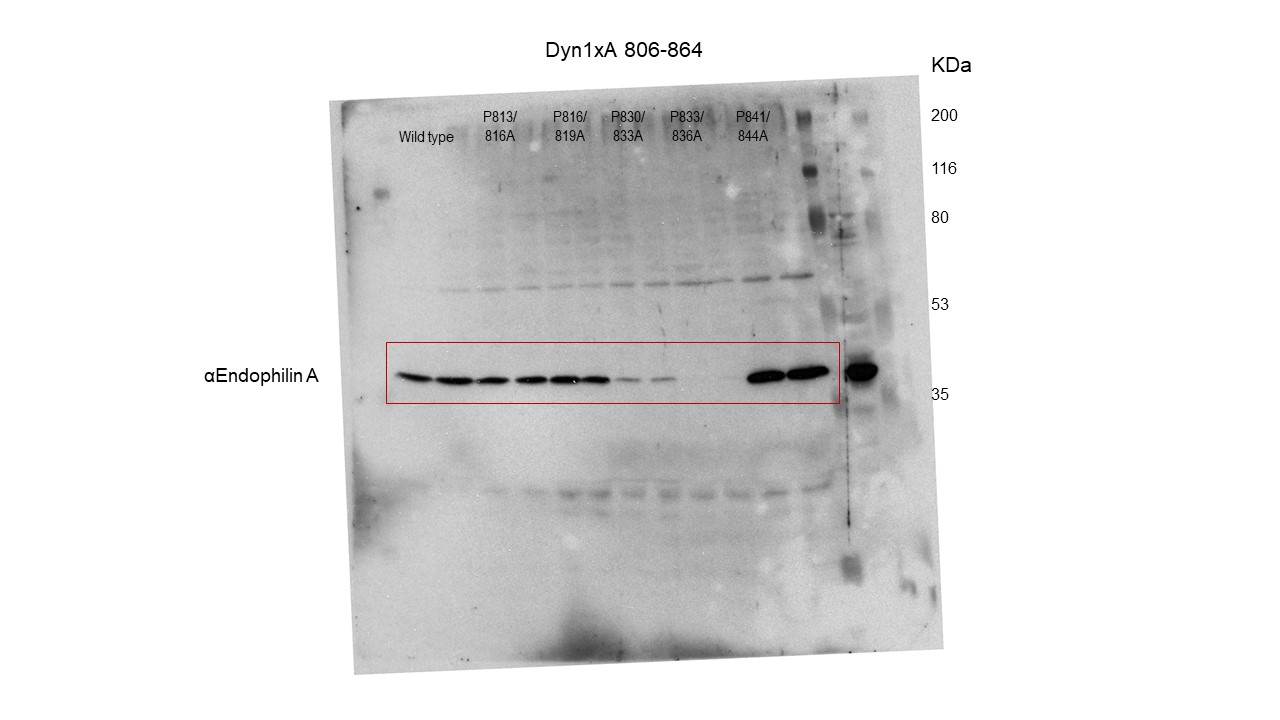

Supplement: Supplementary file 5 — Source data Fig. 3 [file 44318_2024_145_MOESM5_ESM.zip › Source_data_Figure_3/3B/Western Endophilin.jpg]

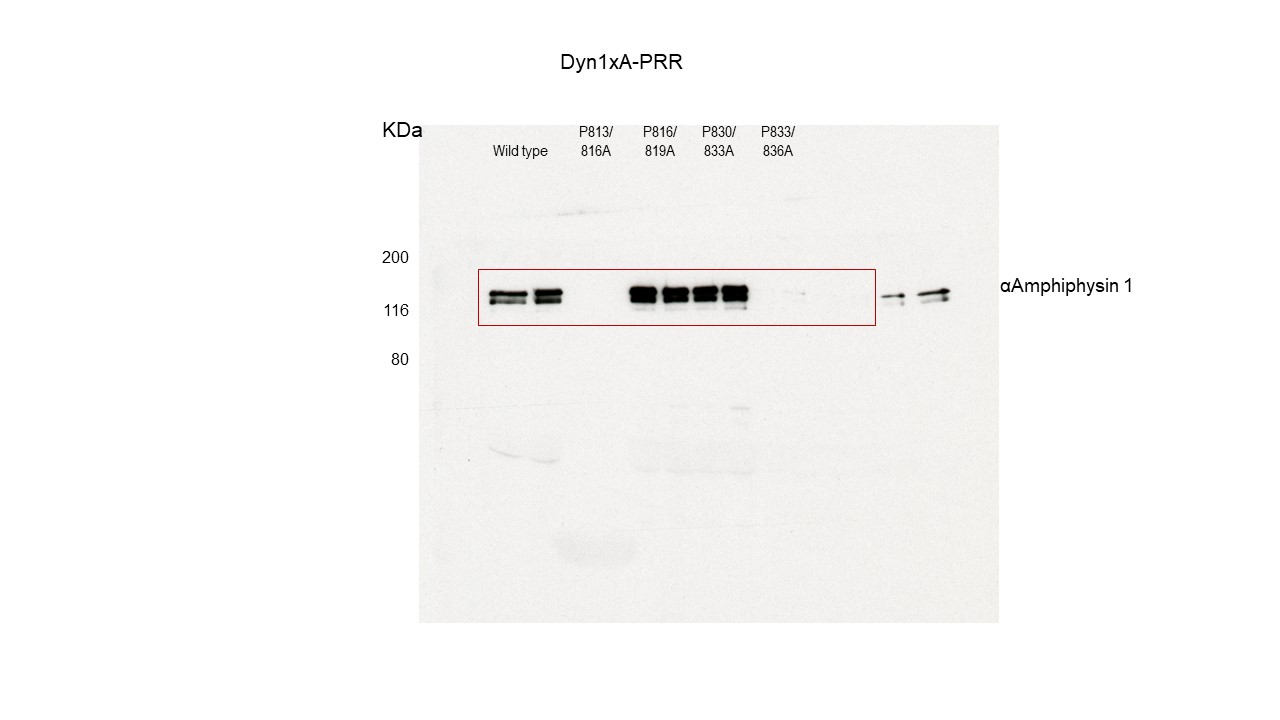

Supplement: Supplementary file 5 — Source data Fig. 3 [file 44318_2024_145_MOESM5_ESM.zip › Source_data_Figure_3/3C/Western Amphiphysin.jpg]

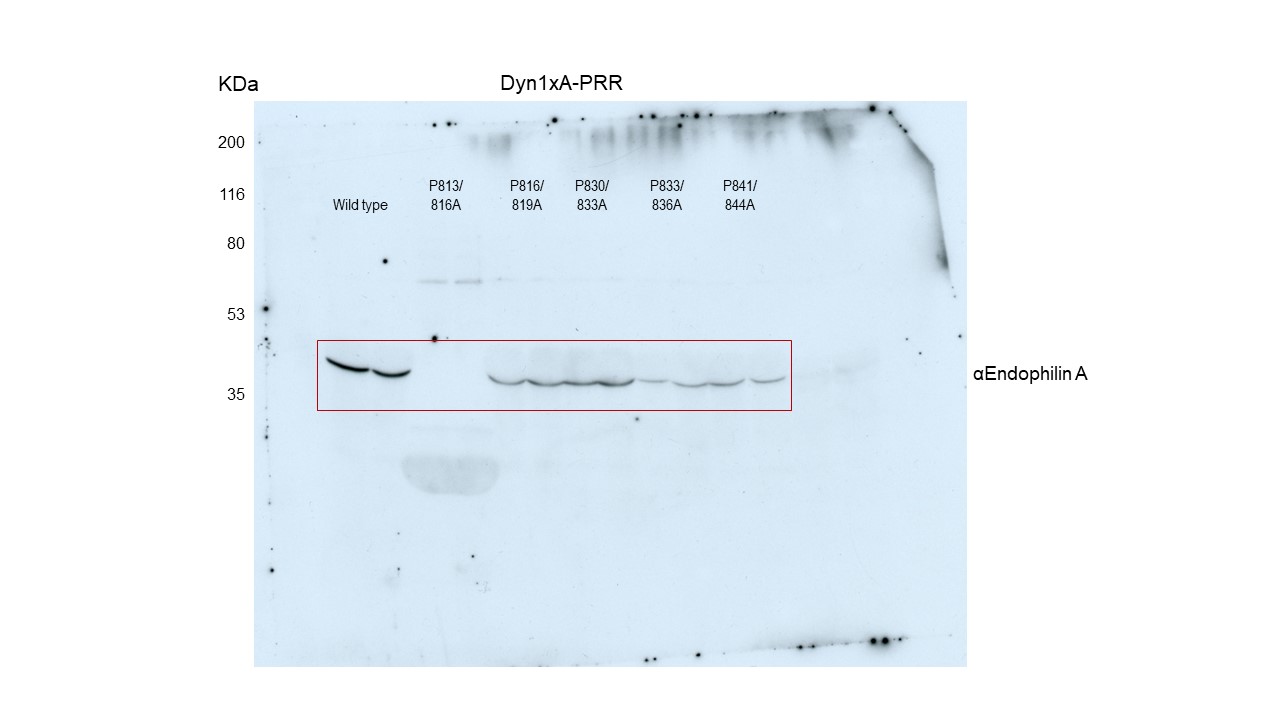

Supplement: Supplementary file 5 — Source data Fig. 3 [file 44318_2024_145_MOESM5_ESM.zip › Source_data_Figure_3/3C/Western Endophilin.jpg]

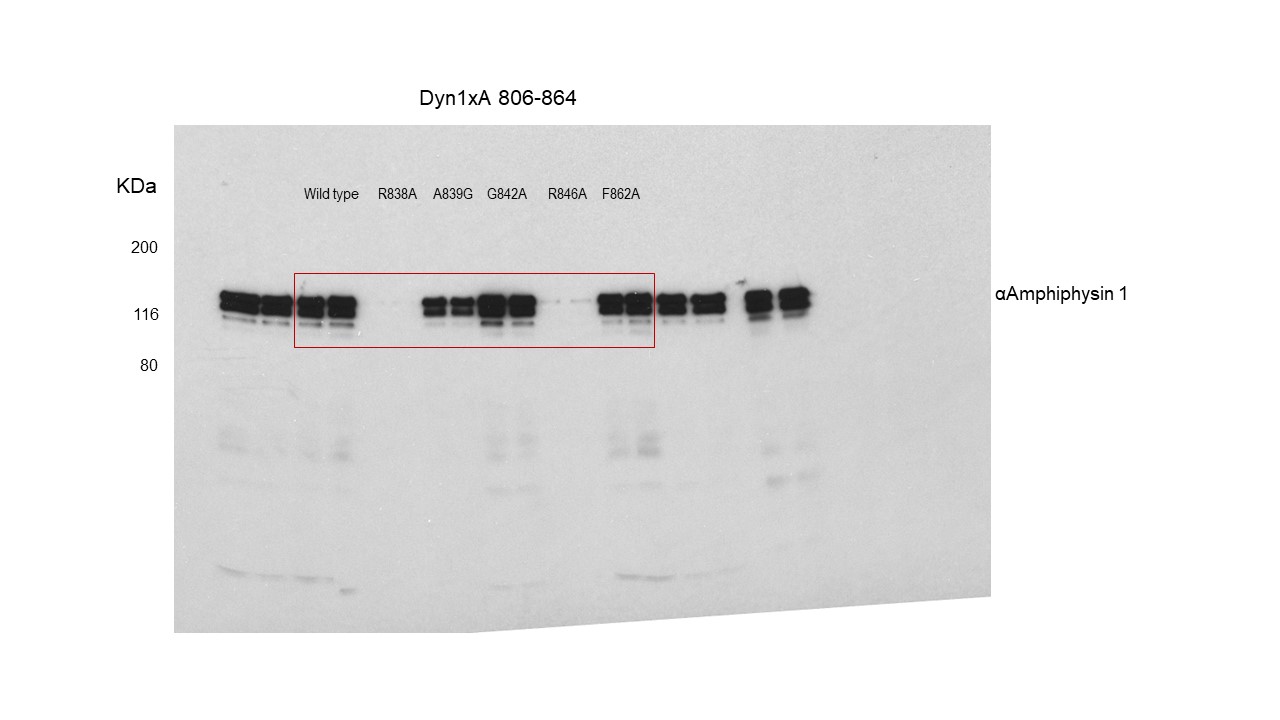

Supplement: Supplementary file 5 — Source data Fig. 3 [file 44318_2024_145_MOESM5_ESM.zip › Source_data_Figure_3/3D/Western Amphiphysin.jpg]

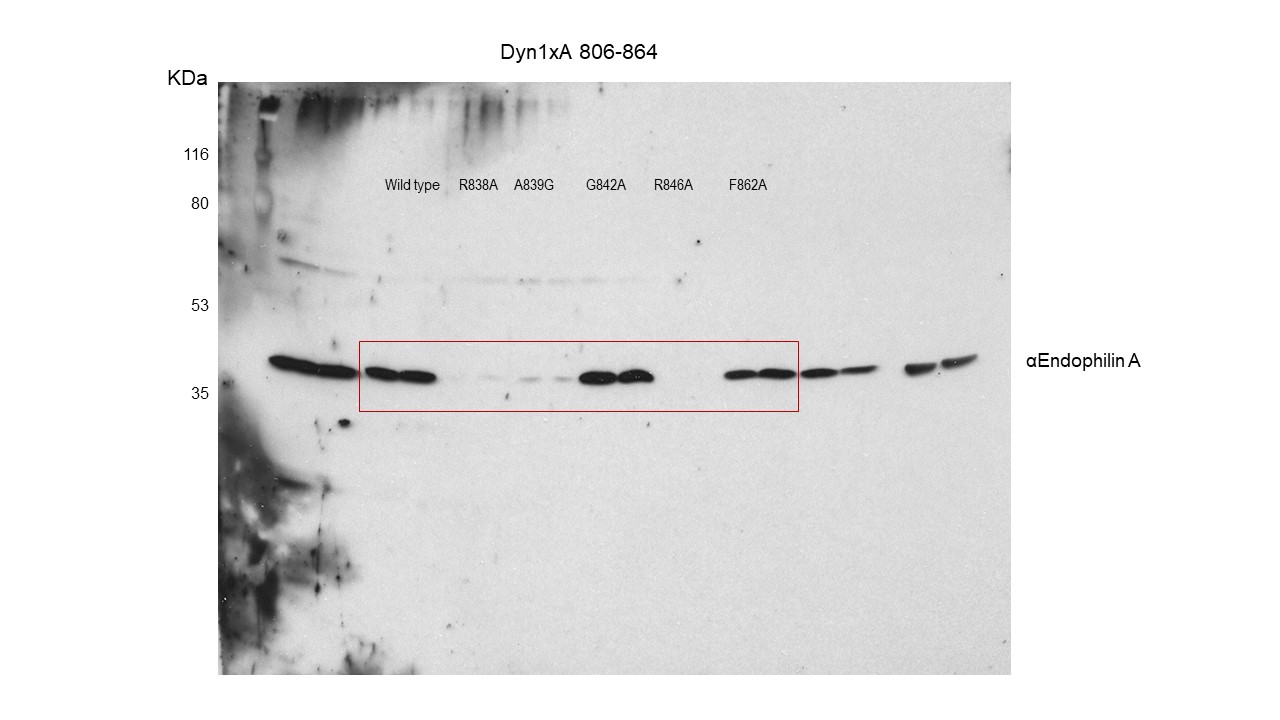

Supplement: Supplementary file 5 — Source data Fig. 3 [file 44318_2024_145_MOESM5_ESM.zip › Source_data_Figure_3/3D/Western Endophilin.jpg]

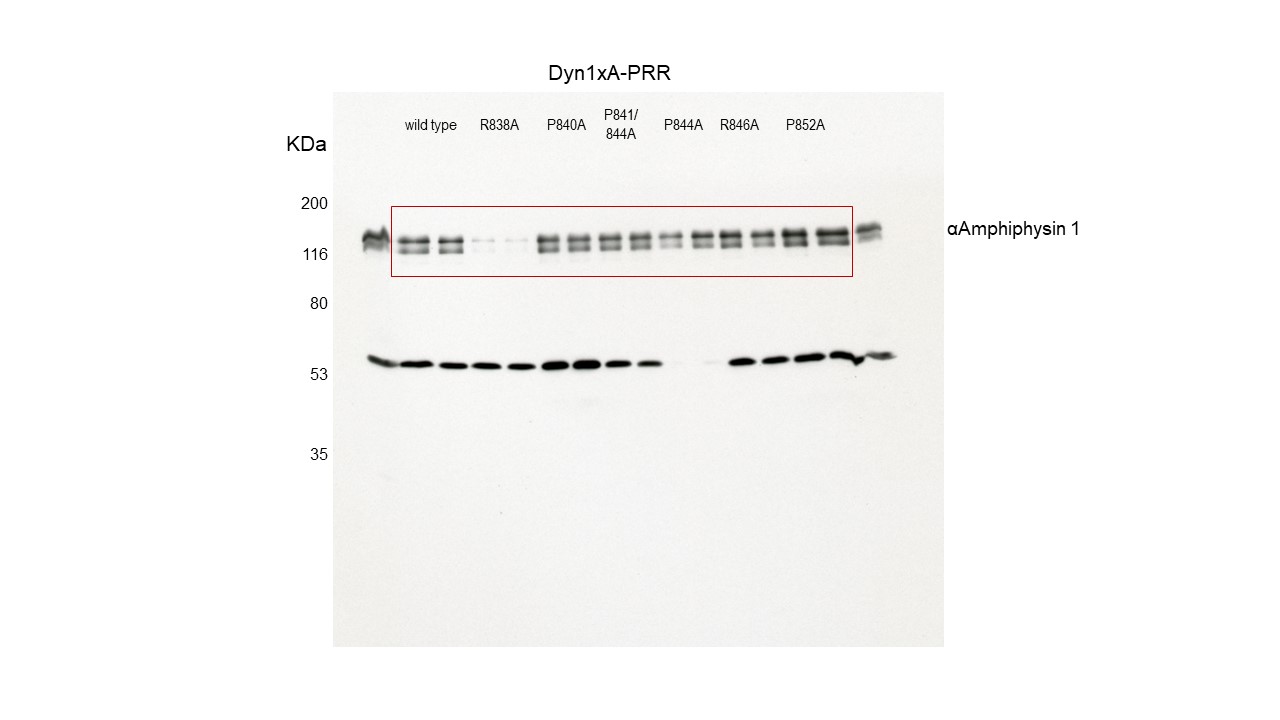

Supplement: Supplementary file 5 — Source data Fig. 3 [file 44318_2024_145_MOESM5_ESM.zip › Source_data_Figure_3/3E/Western Amphiphysin.jpg]

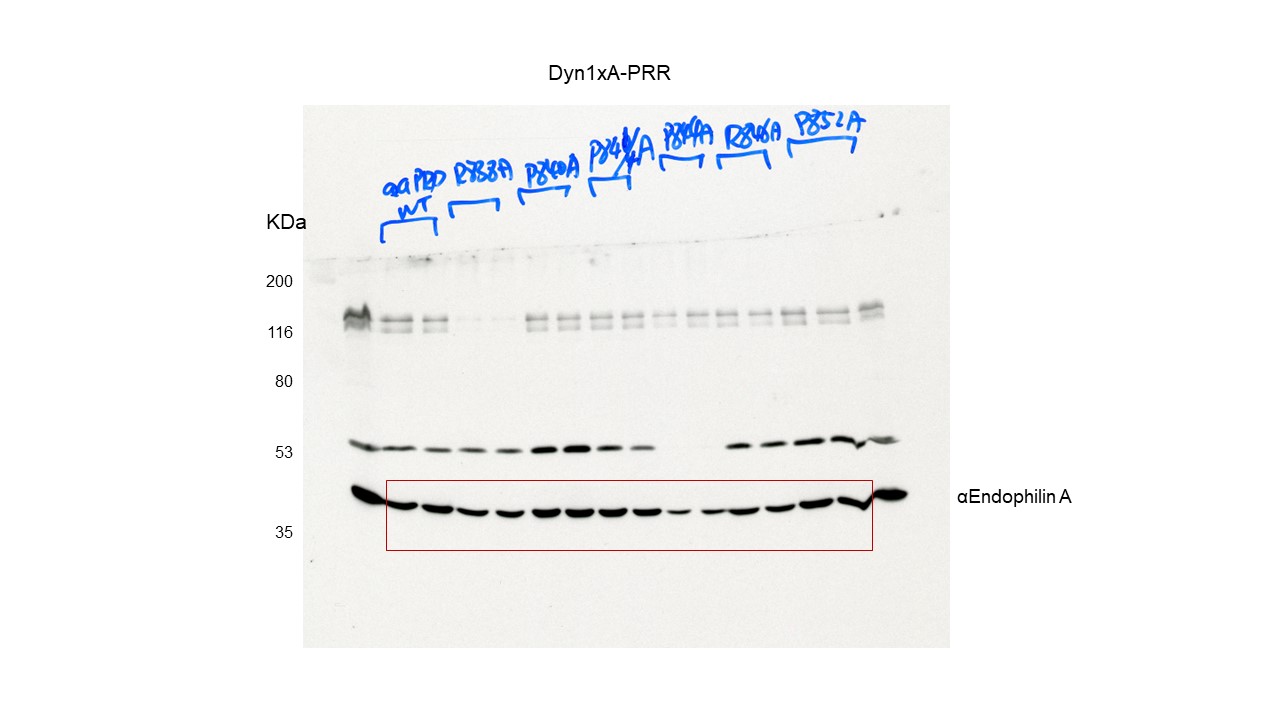

Supplement: Supplementary file 5 — Source data Fig. 3 [file 44318_2024_145_MOESM5_ESM.zip › Source_data_Figure_3/3E/Western Endophilin.jpg]

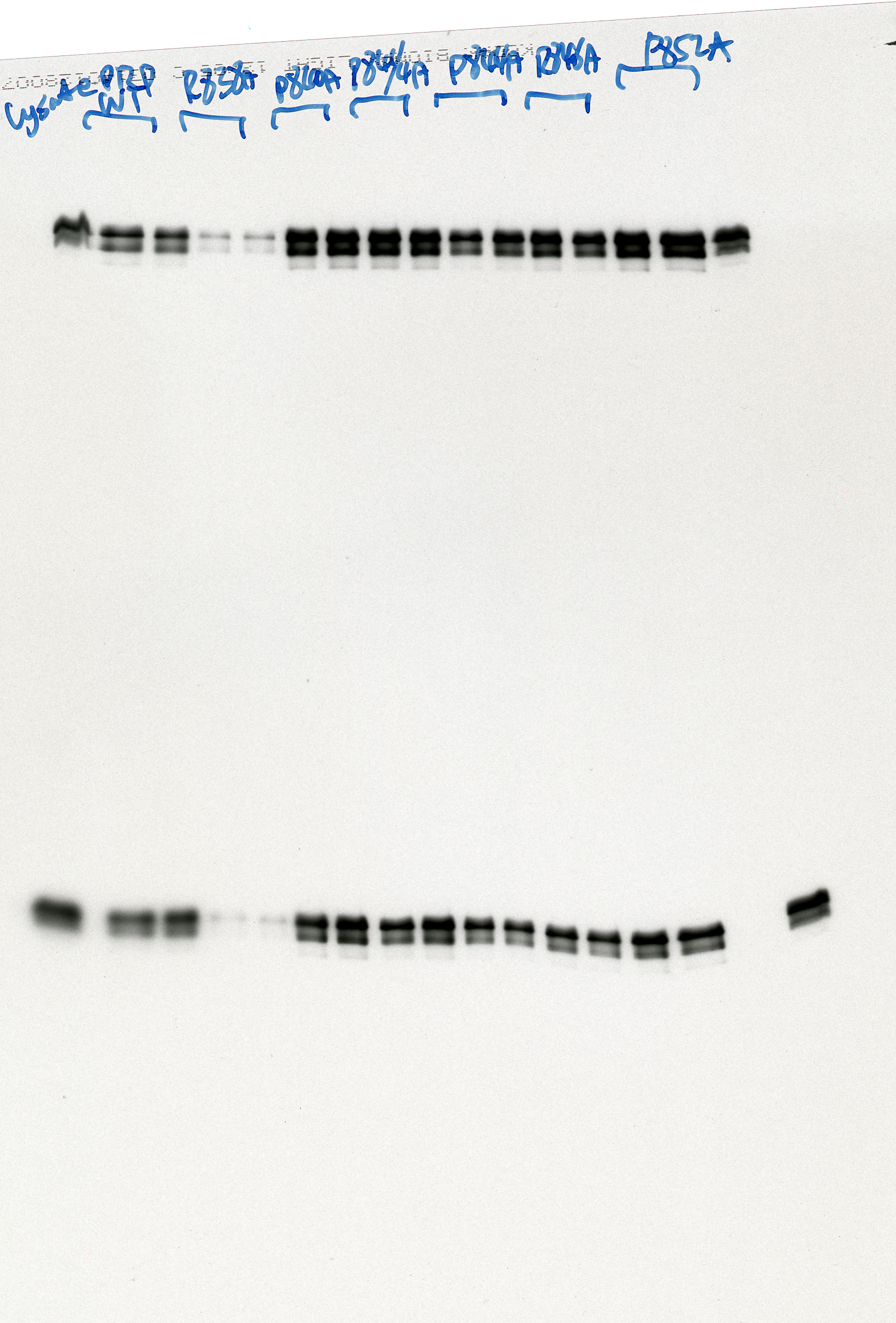

Supplement: Supplementary file 5 — Source data Fig. 3 [file 44318_2024_145_MOESM5_ESM.zip › Source_data_Figure_3/3F and 3G/Original WB Scans for endo binding to R838A mutant quantification/jx-131_Amph.tif]

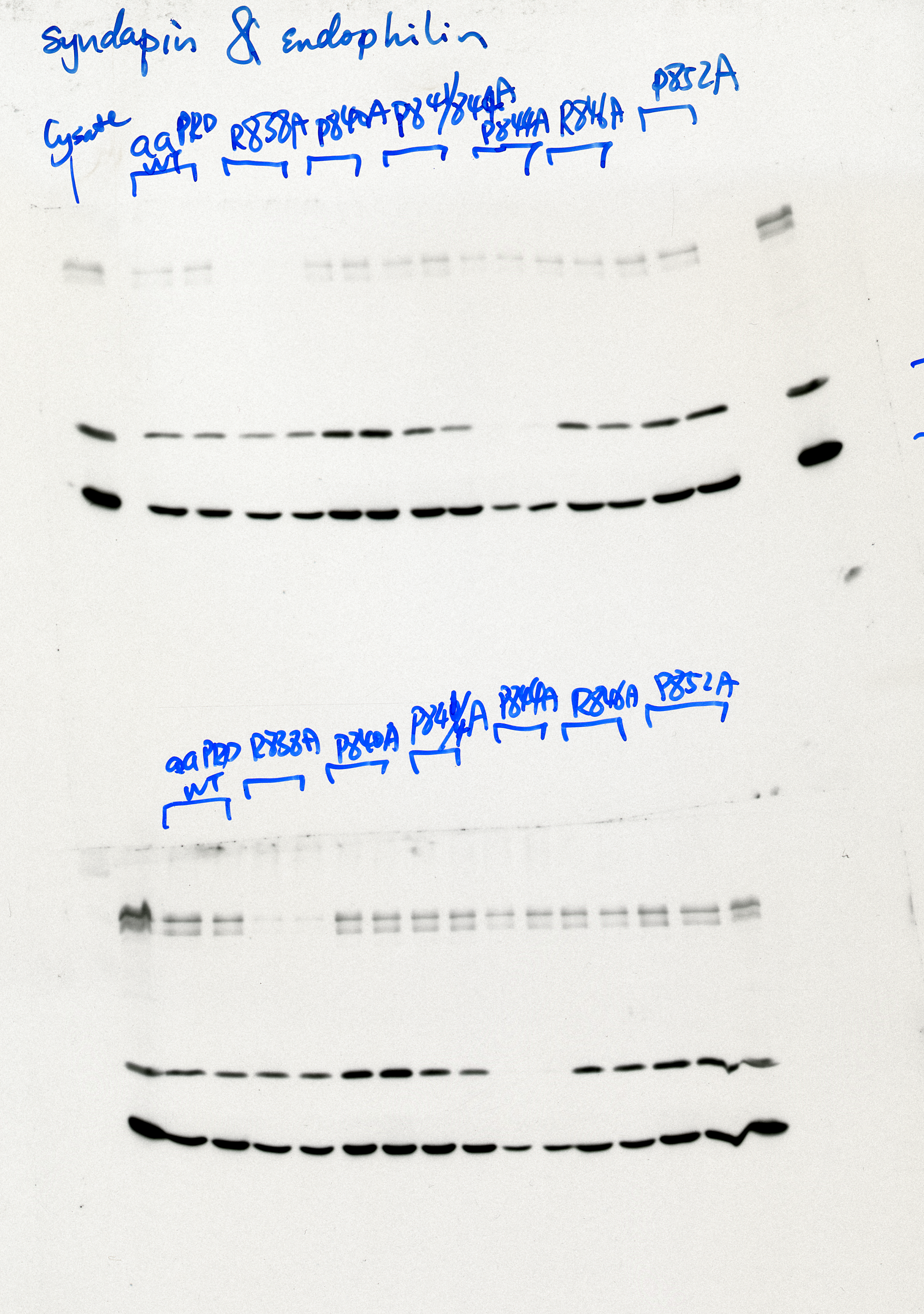

Supplement: Supplementary file 5 — Source data Fig. 3 [file 44318_2024_145_MOESM5_ESM.zip › Source_data_Figure_3/3F and 3G/Original WB Scans for endo binding to R838A mutant quantification/jx-131_endo and synda.tif]

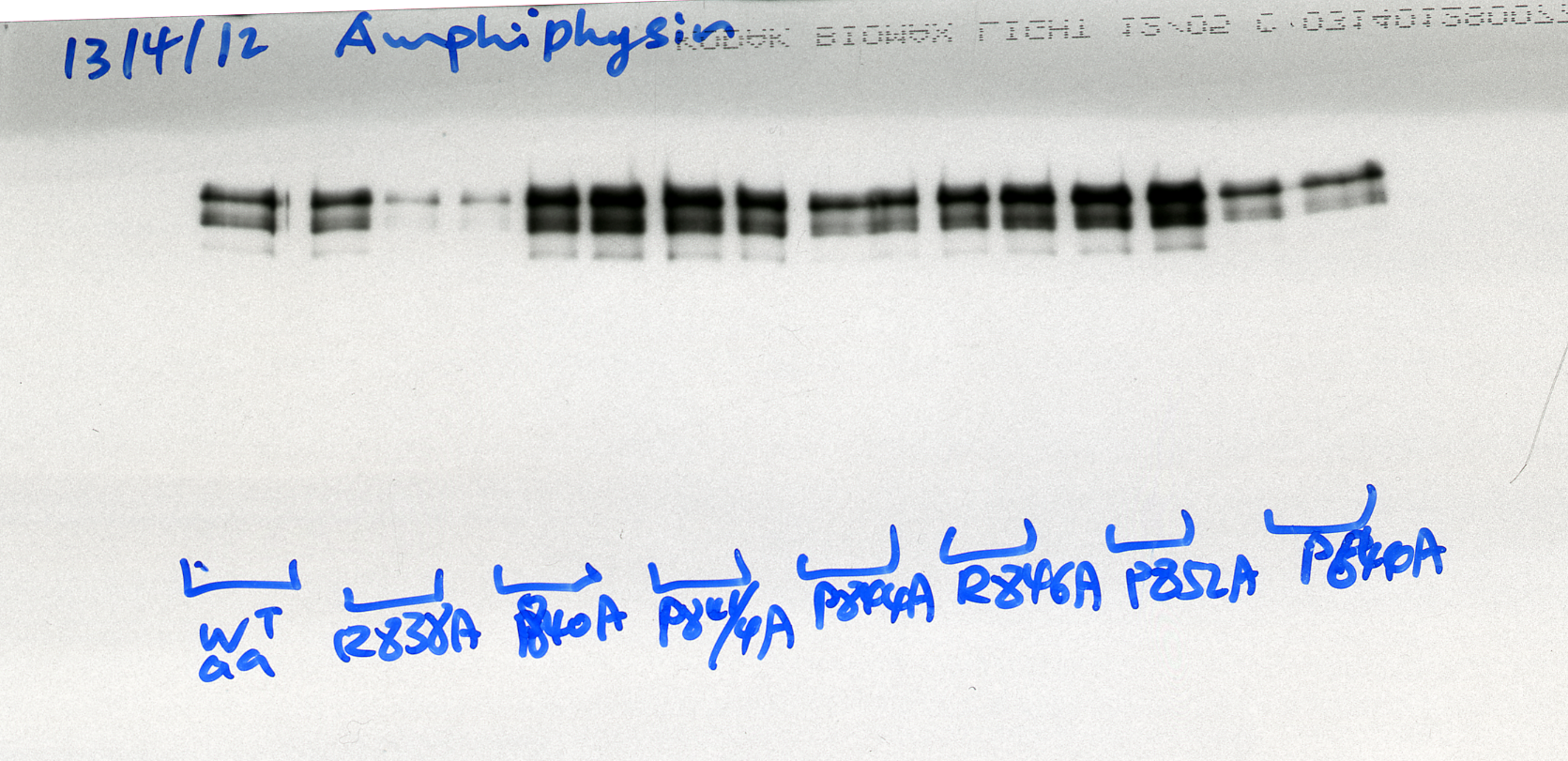

Supplement: Supplementary file 5 — Source data Fig. 3 [file 44318_2024_145_MOESM5_ESM.zip › Source_data_Figure_3/3F and 3G/Original WB Scans for endo binding to R838A mutant quantification/jx-132_Amph.tif]

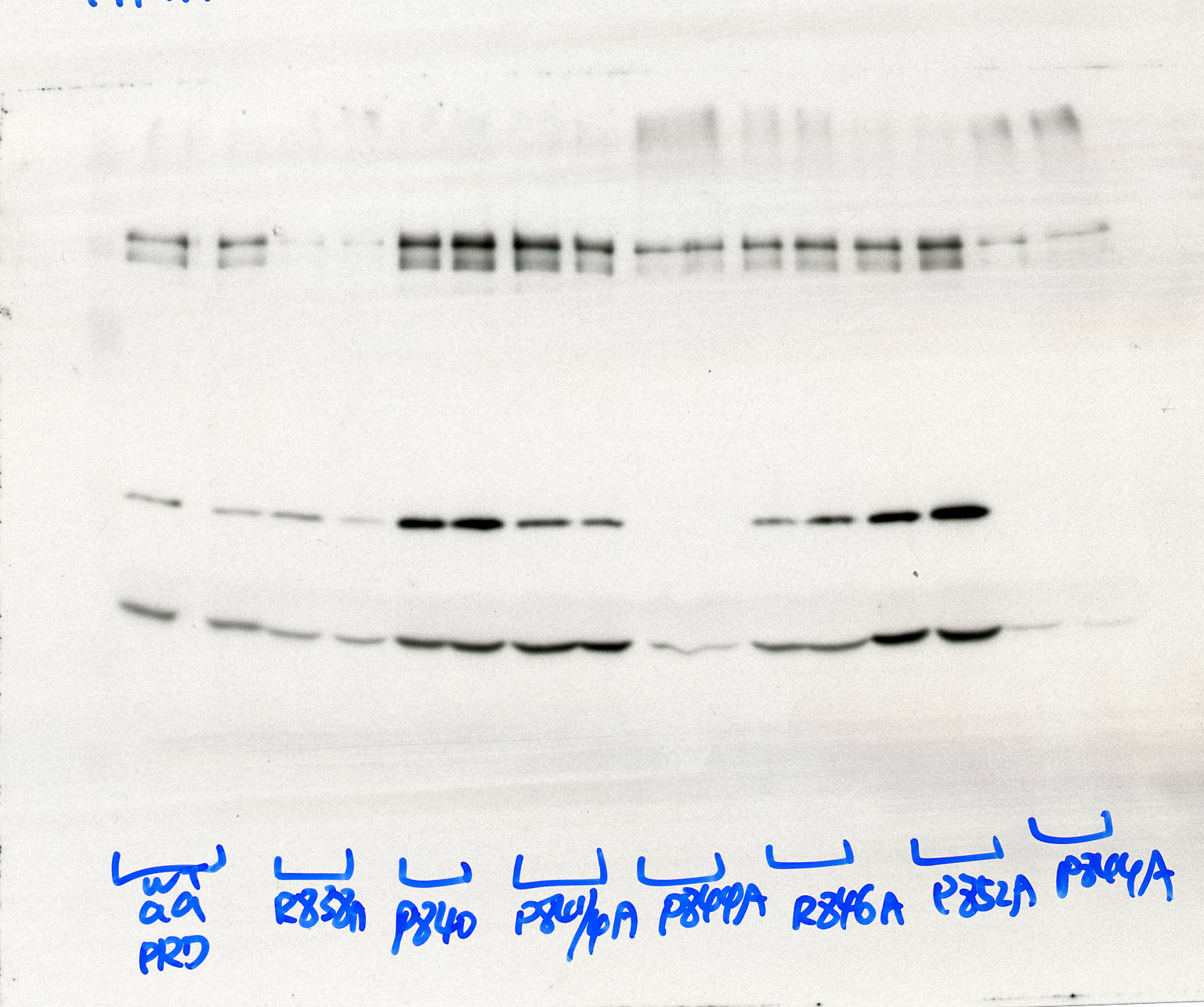

Supplement: Supplementary file 5 — Source data Fig. 3 [file 44318_2024_145_MOESM5_ESM.zip › Source_data_Figure_3/3F and 3G/Original WB Scans for endo binding to R838A mutant quantification/jx-132_endo.tif]

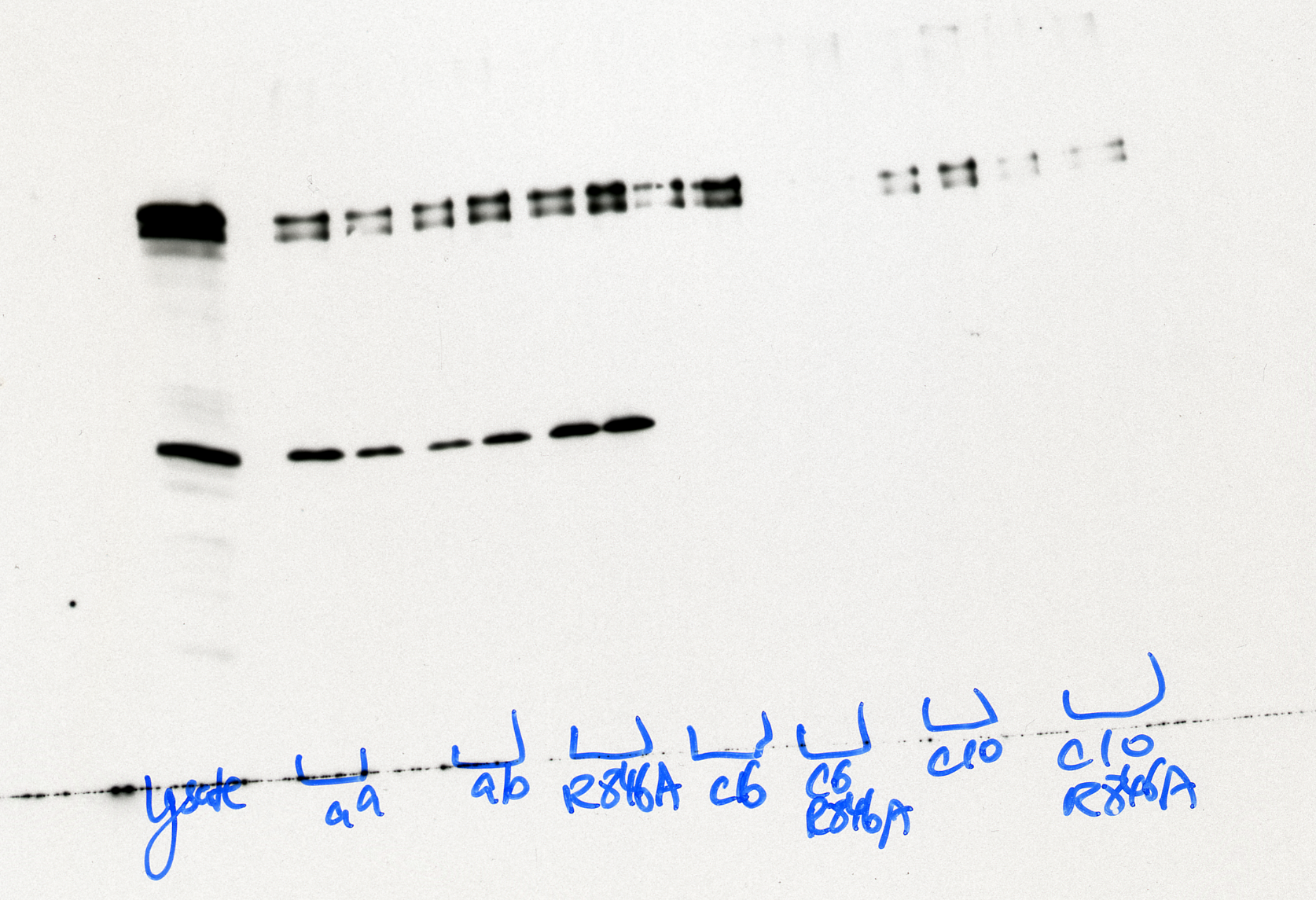

Supplement: Supplementary file 5 — Source data Fig. 3 [file 44318_2024_145_MOESM5_ESM.zip › Source_data_Figure_3/3F and 3G/Original WB Scans for endo binding to R846A mutant quantification/jx-123_Amph and synda.tif]

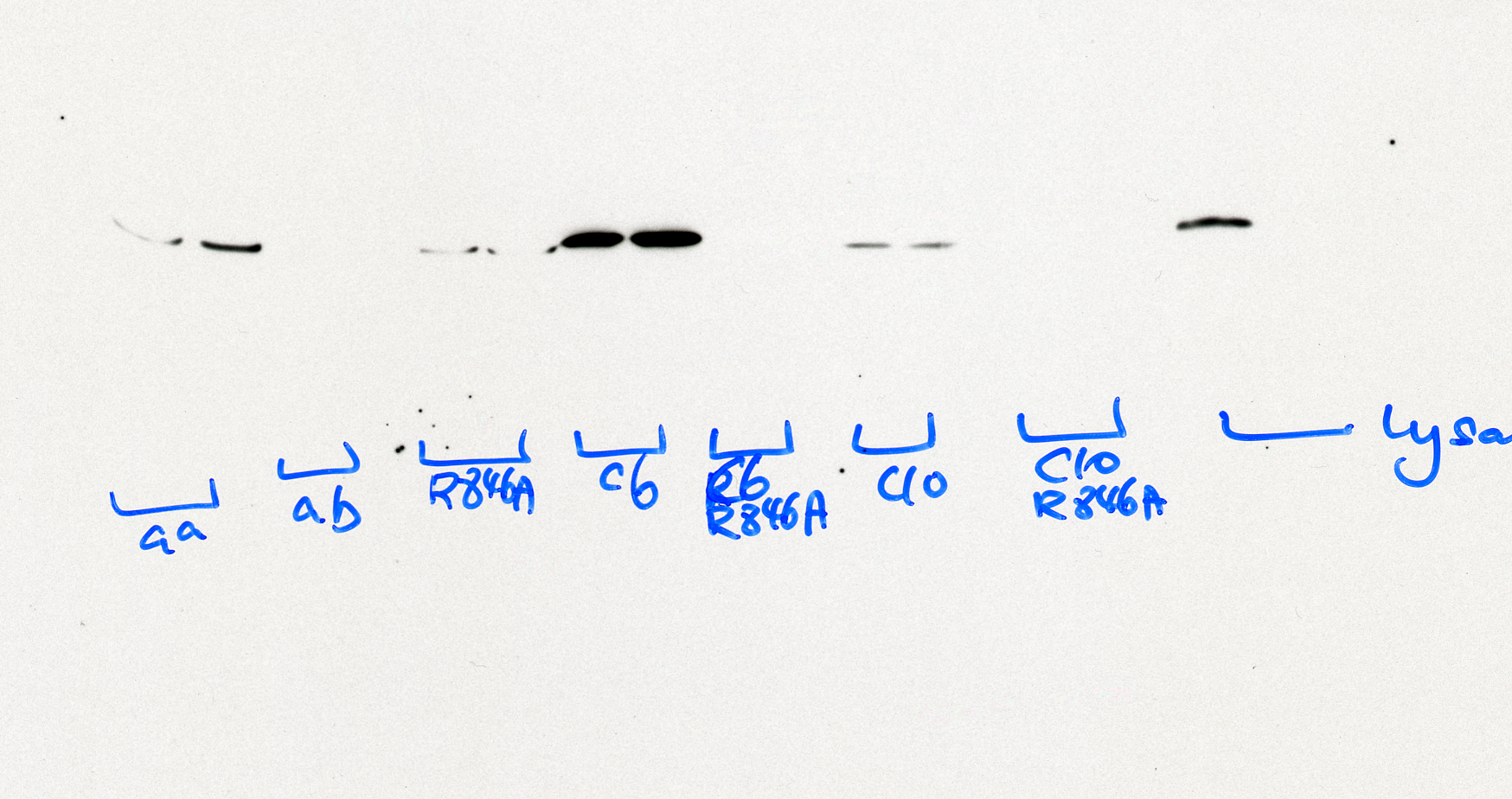

Supplement: Supplementary file 5 — Source data Fig. 3 [file 44318_2024_145_MOESM5_ESM.zip › Source_data_Figure_3/3F and 3G/Original WB Scans for endo binding to R846A mutant quantification/jx-123_endo.tif]

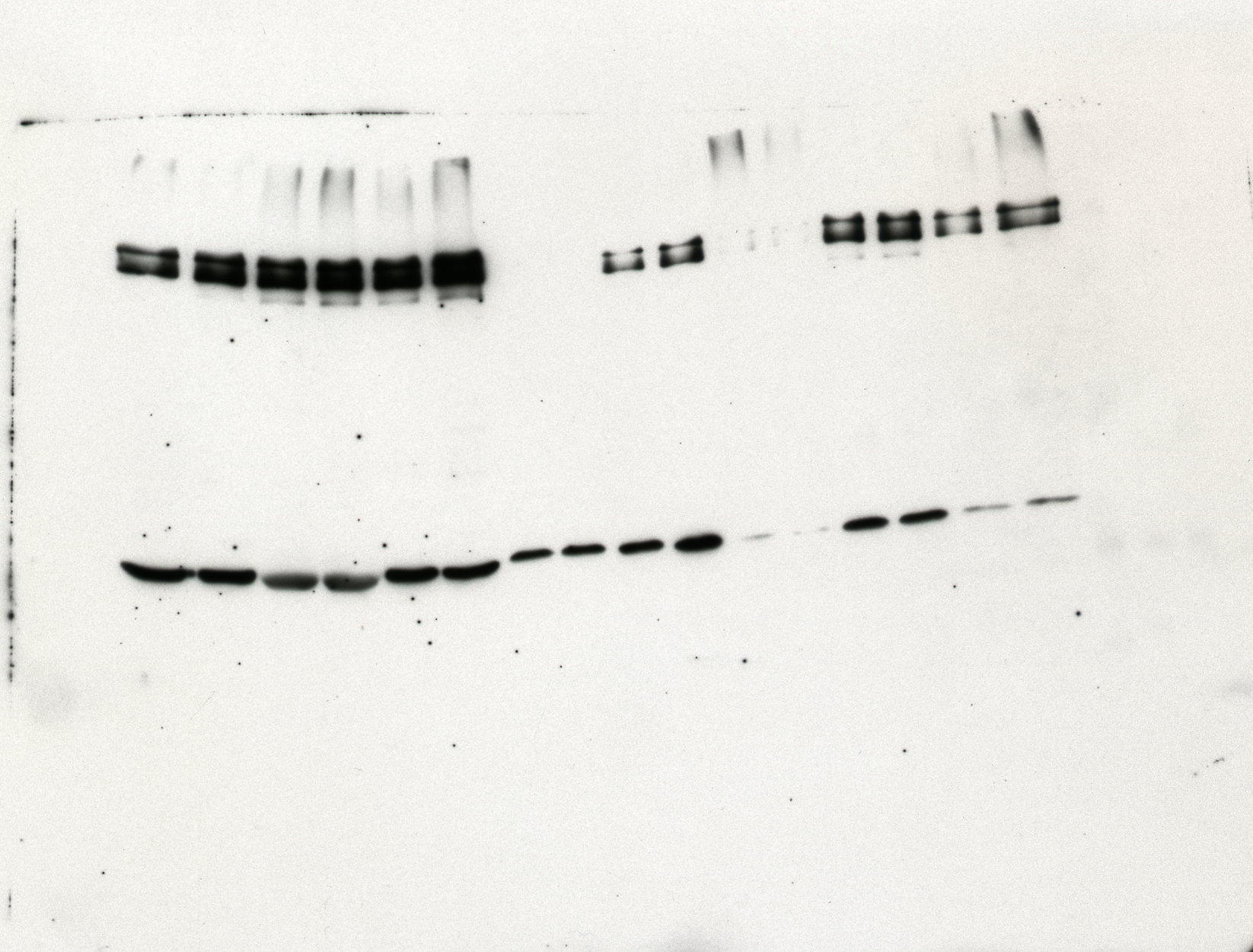

Supplement: Supplementary file 5 — Source data Fig. 3 [file 44318_2024_145_MOESM5_ESM.zip › Source_data_Figure_3/3F and 3G/Original WB Scans for endo binding to R846A mutant quantification/jx-127_Amph.tif]

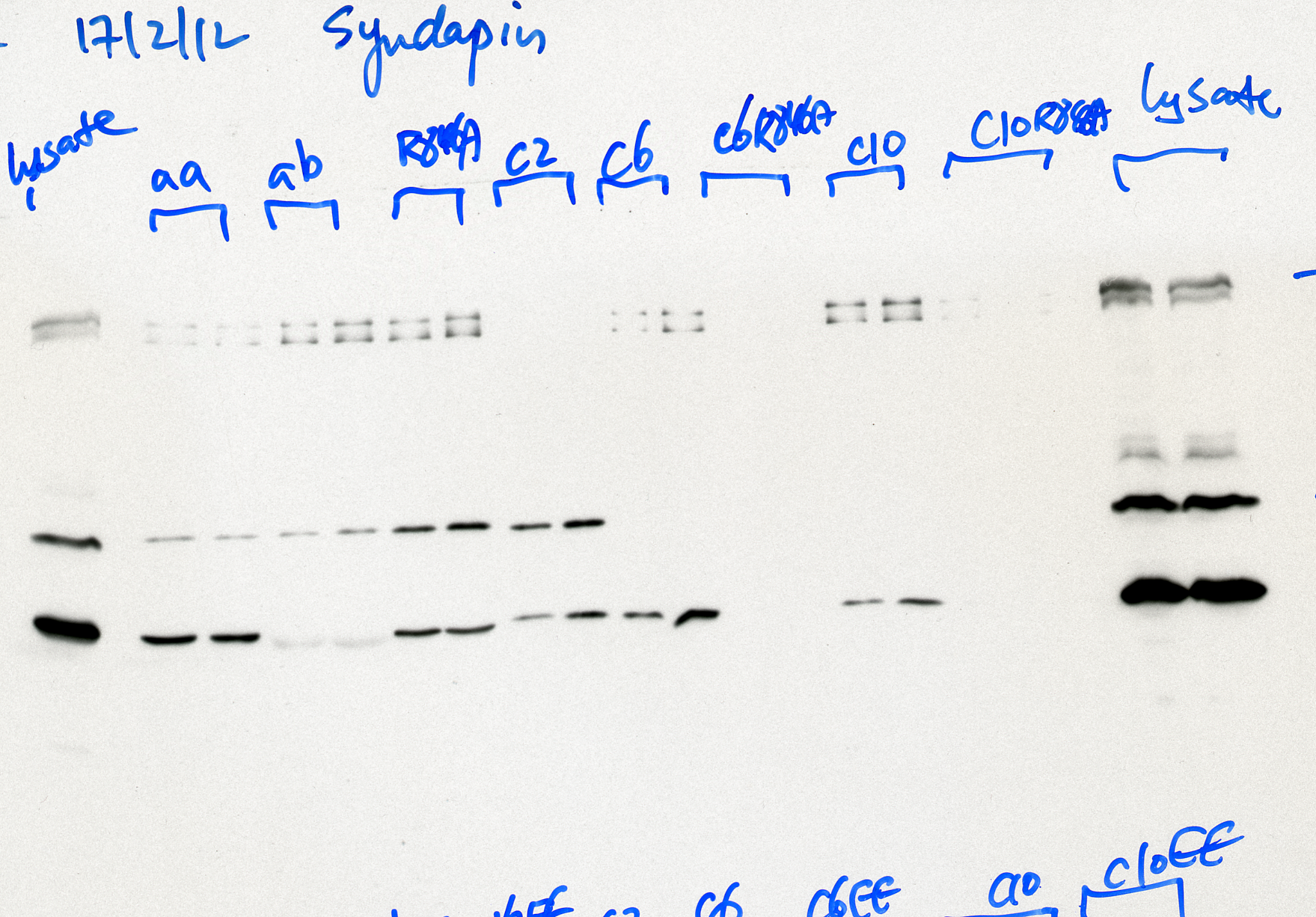

Supplement: Supplementary file 5 — Source data Fig. 3 [file 44318_2024_145_MOESM5_ESM.zip › Source_data_Figure_3/3F and 3G/Original WB Scans for endo binding to R846A mutant quantification/jx-127_endo.tif]

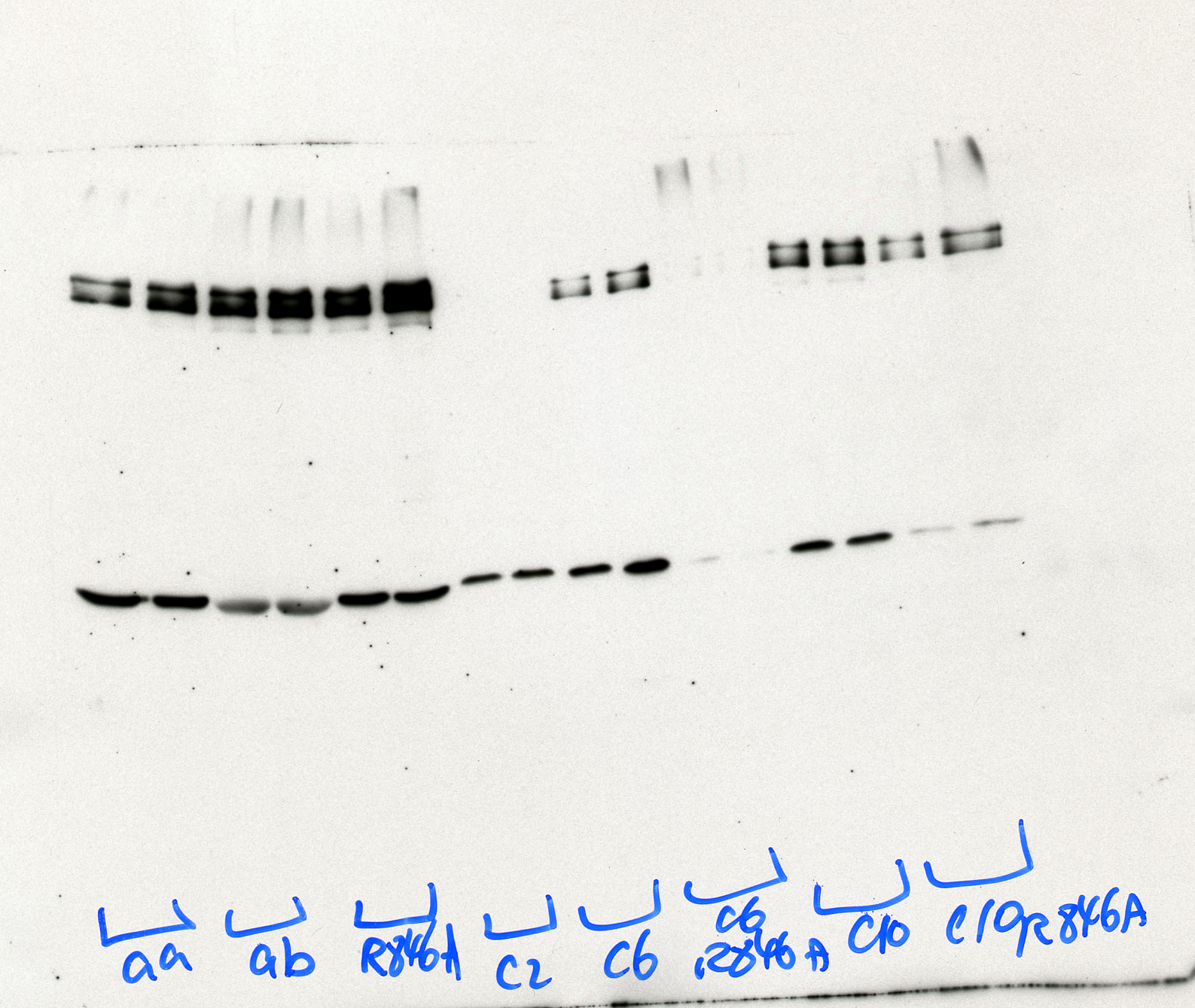

Supplement: Supplementary file 5 — Source data Fig. 3 [file 44318_2024_145_MOESM5_ESM.zip › Source_data_Figure_3/3F and 3G/Original WB Scans for endo binding to R846A mutant quantification/jx-127_repeat_Amph.tif]

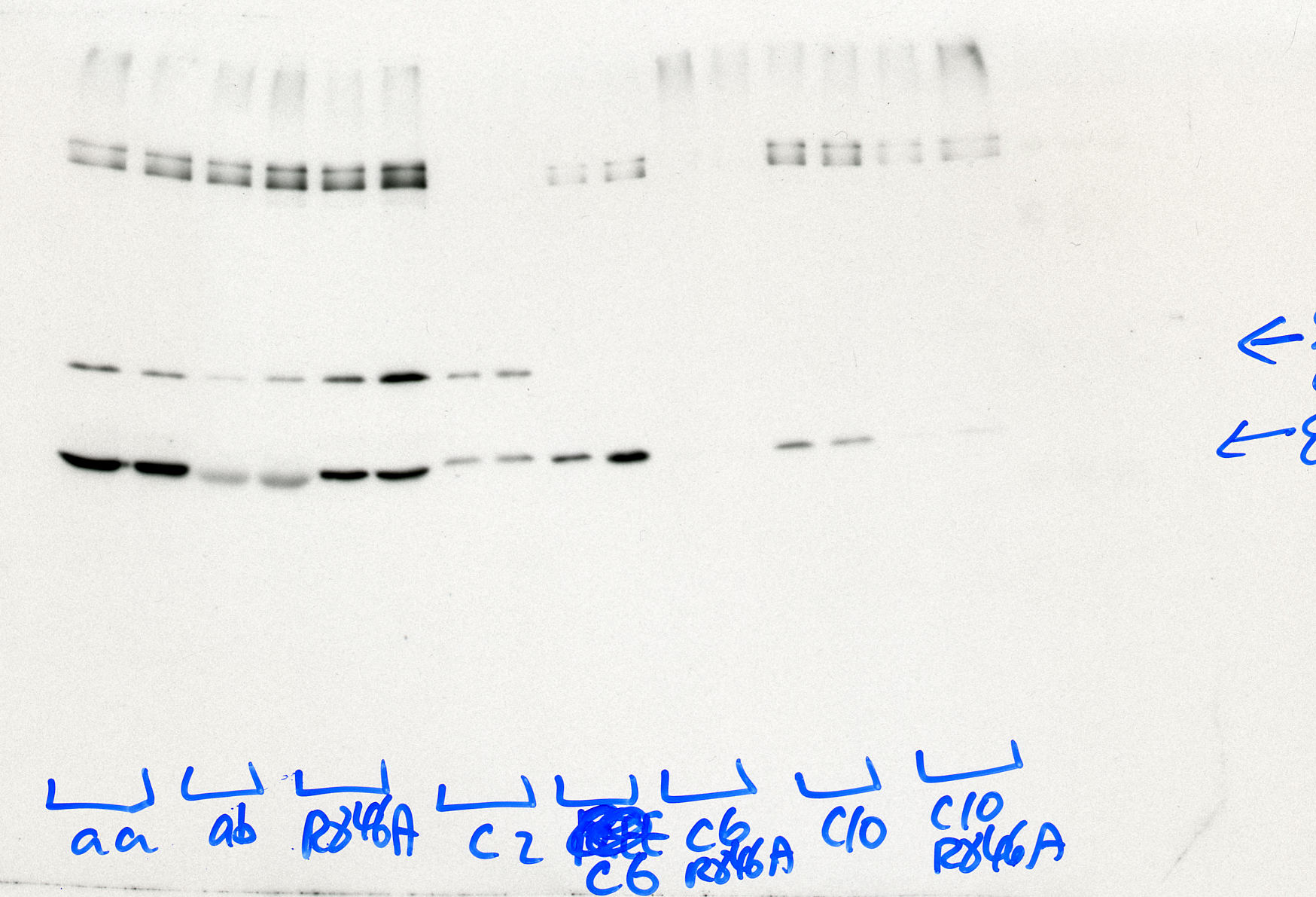

Supplement: Supplementary file 5 — Source data Fig. 3 [file 44318_2024_145_MOESM5_ESM.zip › Source_data_Figure_3/3F and 3G/Original WB Scans for endo binding to R846A mutant quantification/jx-127_repeat_endo and synda.tif]

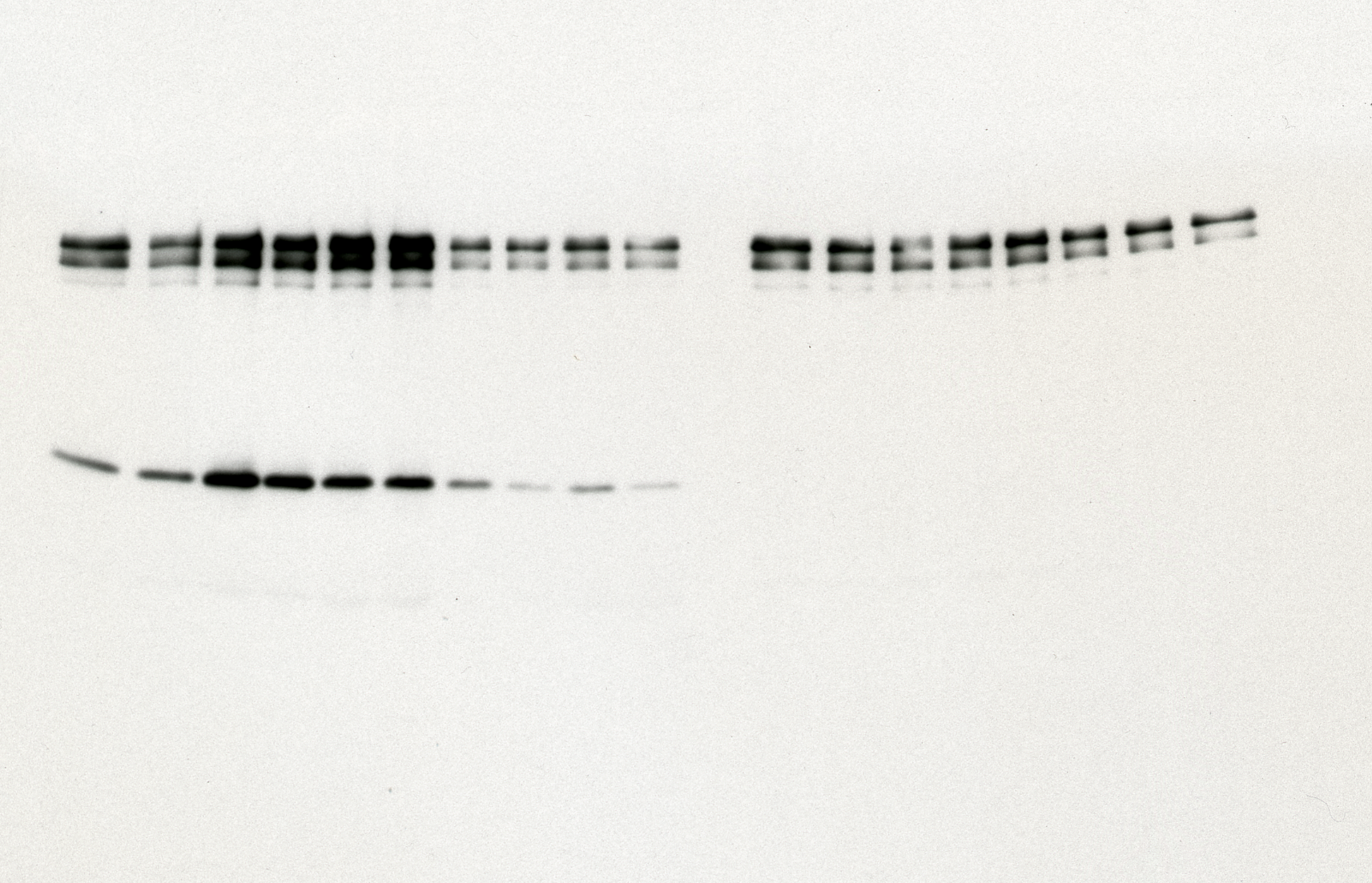

Supplement: Supplementary file 5 — Source data Fig. 3 [file 44318_2024_145_MOESM5_ESM.zip › Source_data_Figure_3/3F and 3G/Original WB Scans for endo binding to R846A mutant quantification/jx-148_Amph and Syndapin.tif]

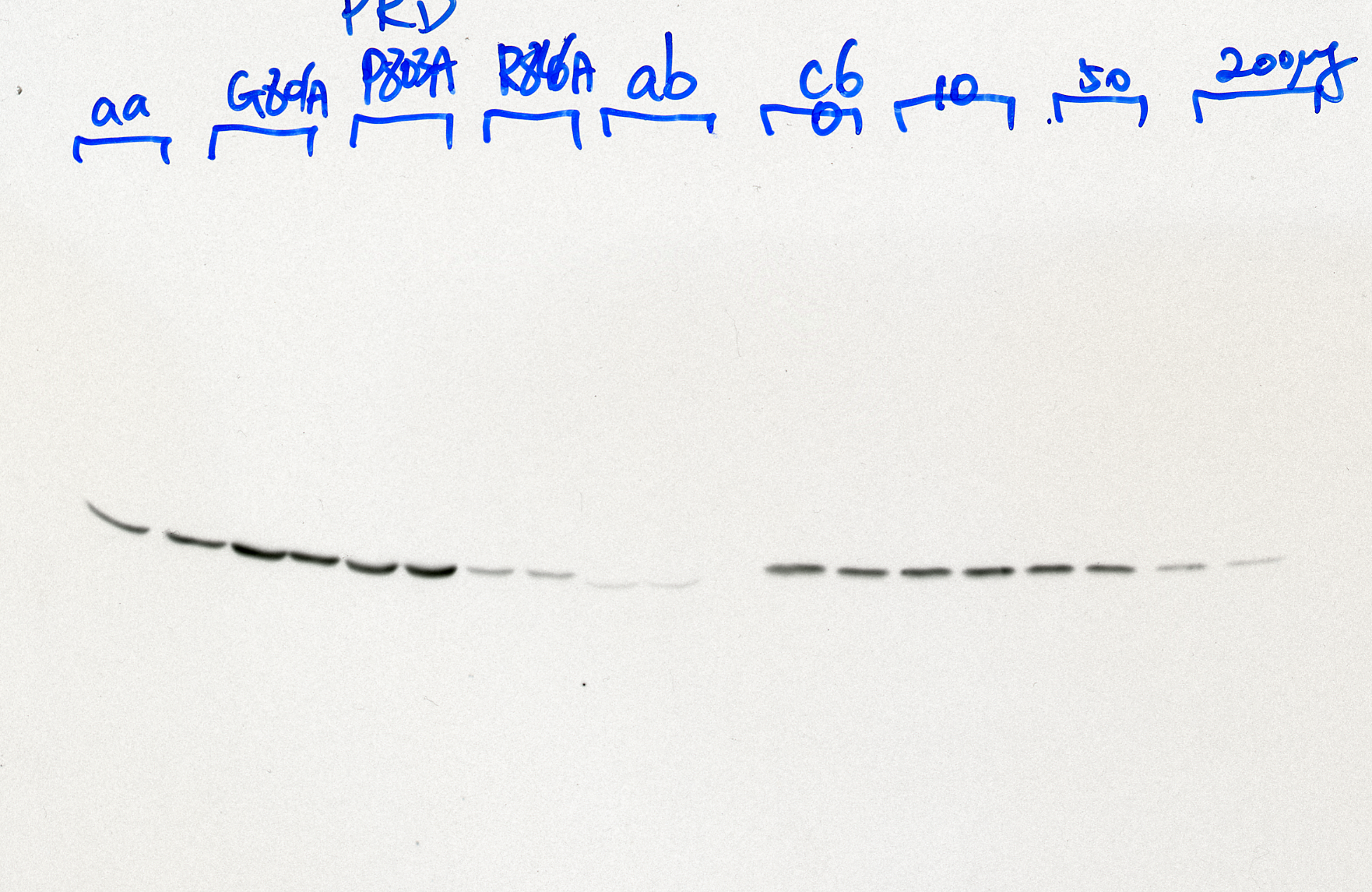

Supplement: Supplementary file 5 — Source data Fig. 3 [file 44318_2024_145_MOESM5_ESM.zip › Source_data_Figure_3/3F and 3G/Original WB Scans for endo binding to R846A mutant quantification/jx-148_endo.tif]

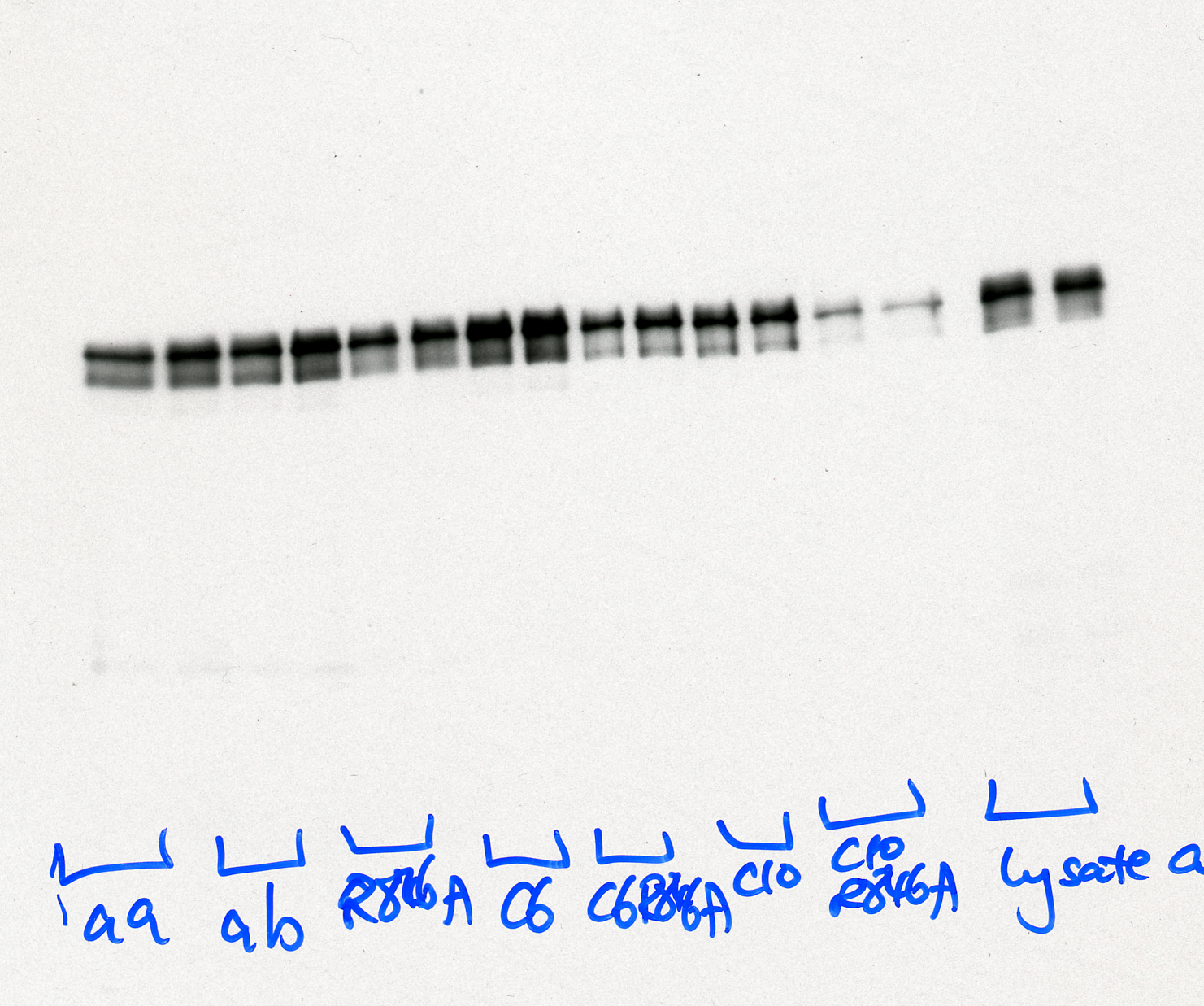

Supplement: Supplementary file 5 — Source data Fig. 3 [file 44318_2024_145_MOESM5_ESM.zip › Source_data_Figure_3/3F and 3G/Original WB Scans for endo binding to R846A mutant quantification/jx-191_amph.tif]

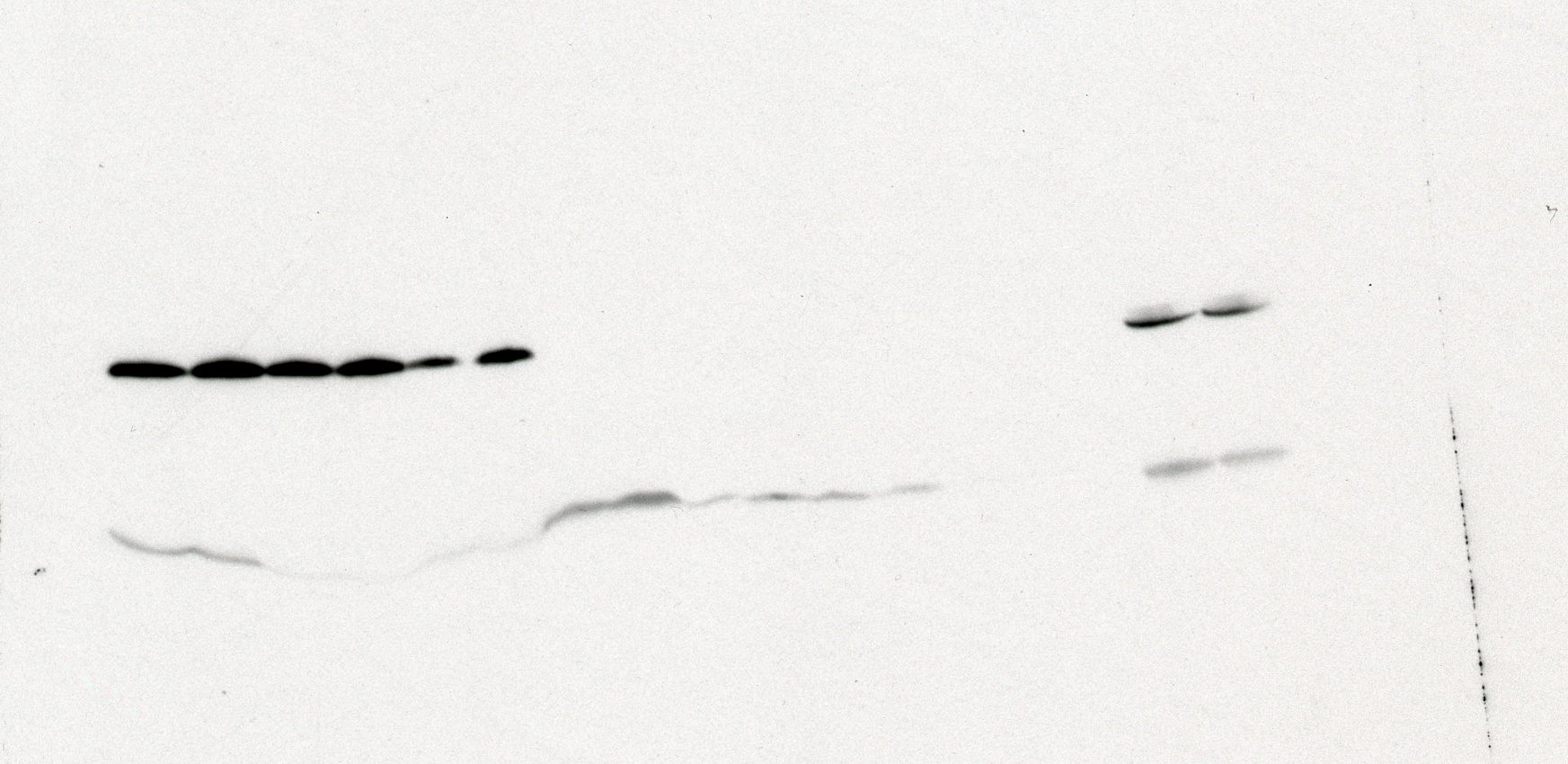

Supplement: Supplementary file 5 — Source data Fig. 3 [file 44318_2024_145_MOESM5_ESM.zip › Source_data_Figure_3/3F and 3G/Original WB Scans for endo binding to R846A mutant quantification/jx-191_synda and endo.tif]

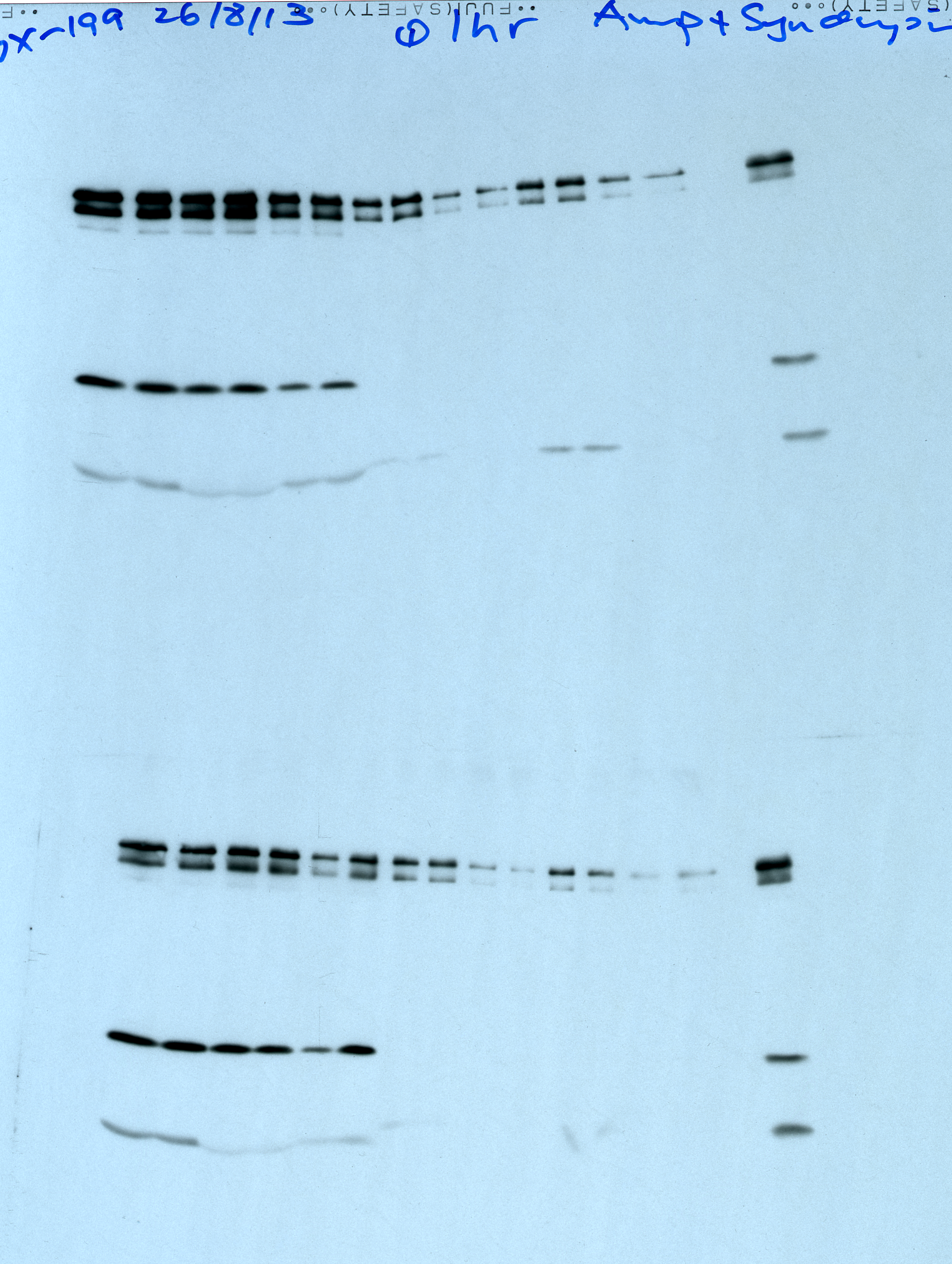

Supplement: Supplementary file 5 — Source data Fig. 3 [file 44318_2024_145_MOESM5_ESM.zip › Source_data_Figure_3/3F and 3G/Original WB Scans for endo binding to R846A mutant quantification/jx-199.tif]

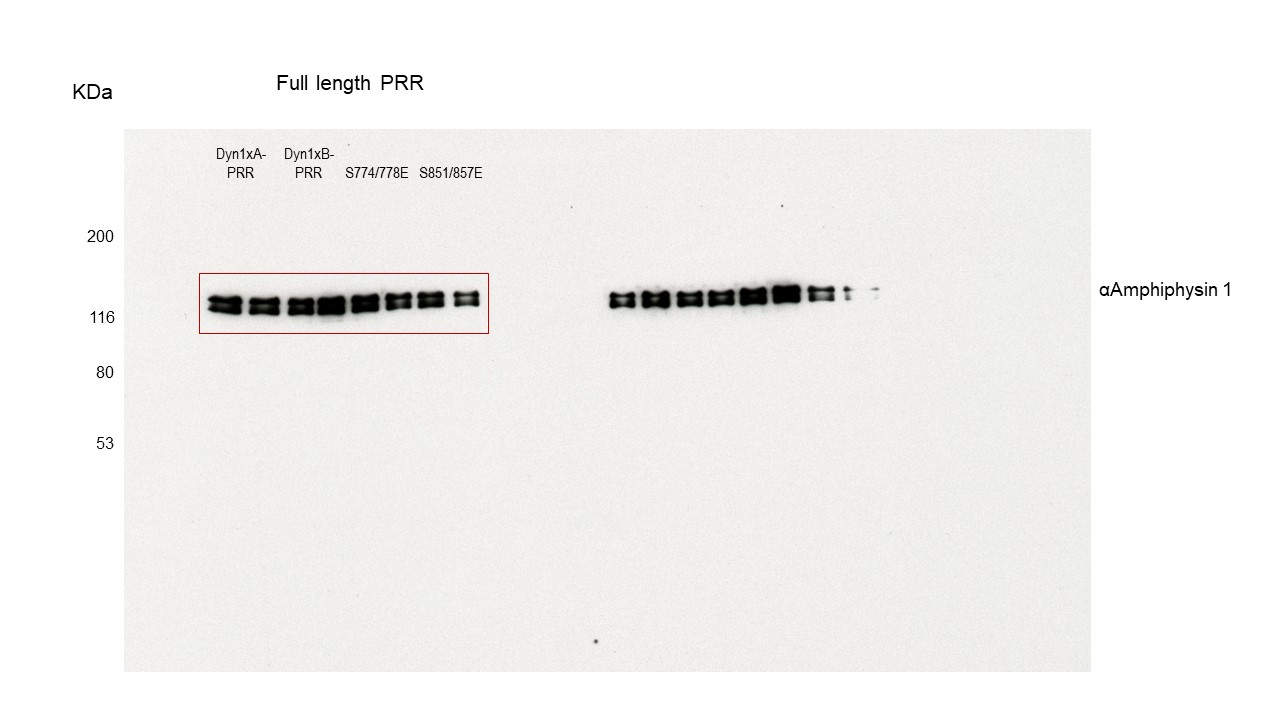

Supplement: Supplementary file 5 — Source data Fig. 3 [file 44318_2024_145_MOESM5_ESM.zip › Source_data_Figure_3/3K/Western Amphiphysin_left panel.jpg]

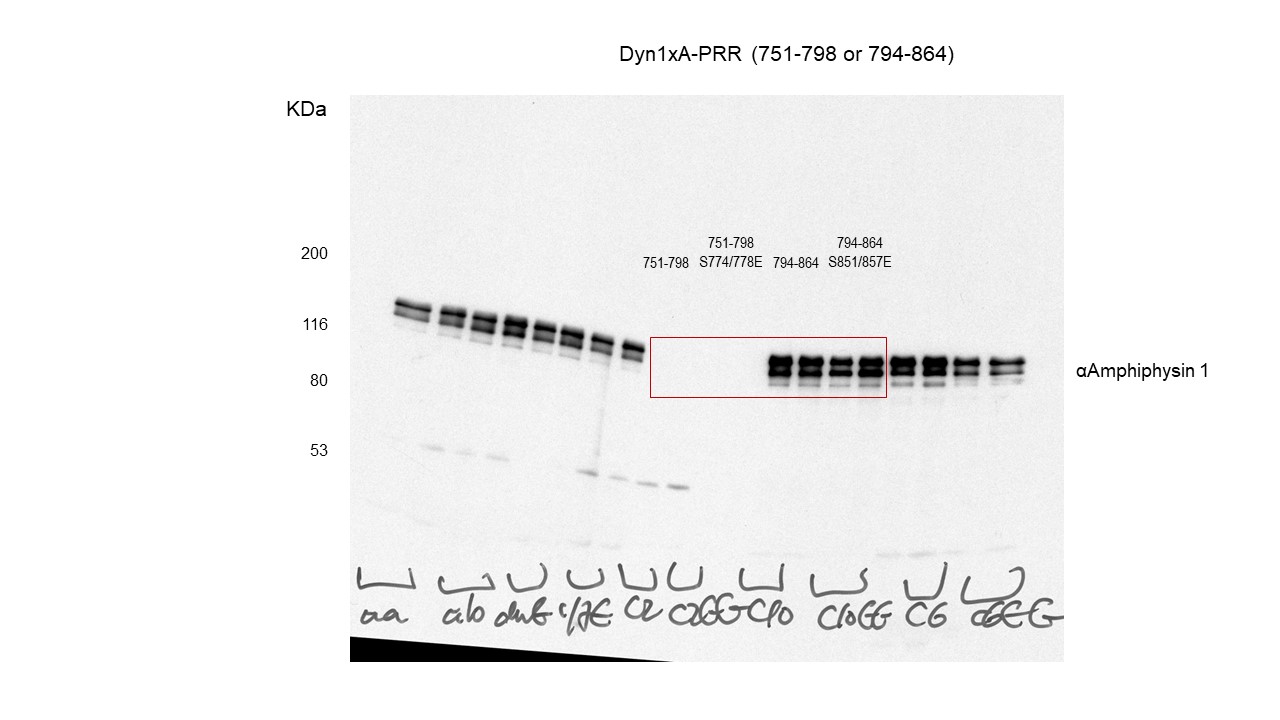

Supplement: Supplementary file 5 — Source data Fig. 3 [file 44318_2024_145_MOESM5_ESM.zip › Source_data_Figure_3/3K/Western Amphiphysin_right panel.jpg]

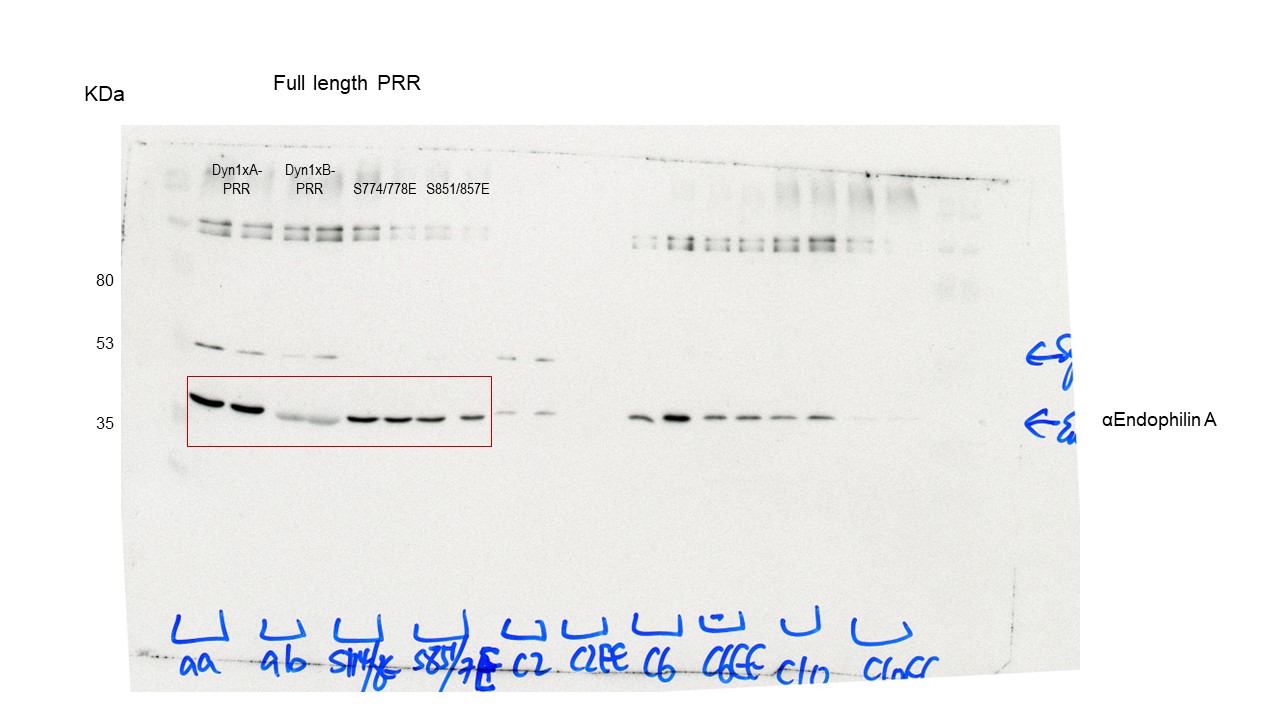

Supplement: Supplementary file 5 — Source data Fig. 3 [file 44318_2024_145_MOESM5_ESM.zip › Source_data_Figure_3/3K/Western Endophilin_left panel.jpg]

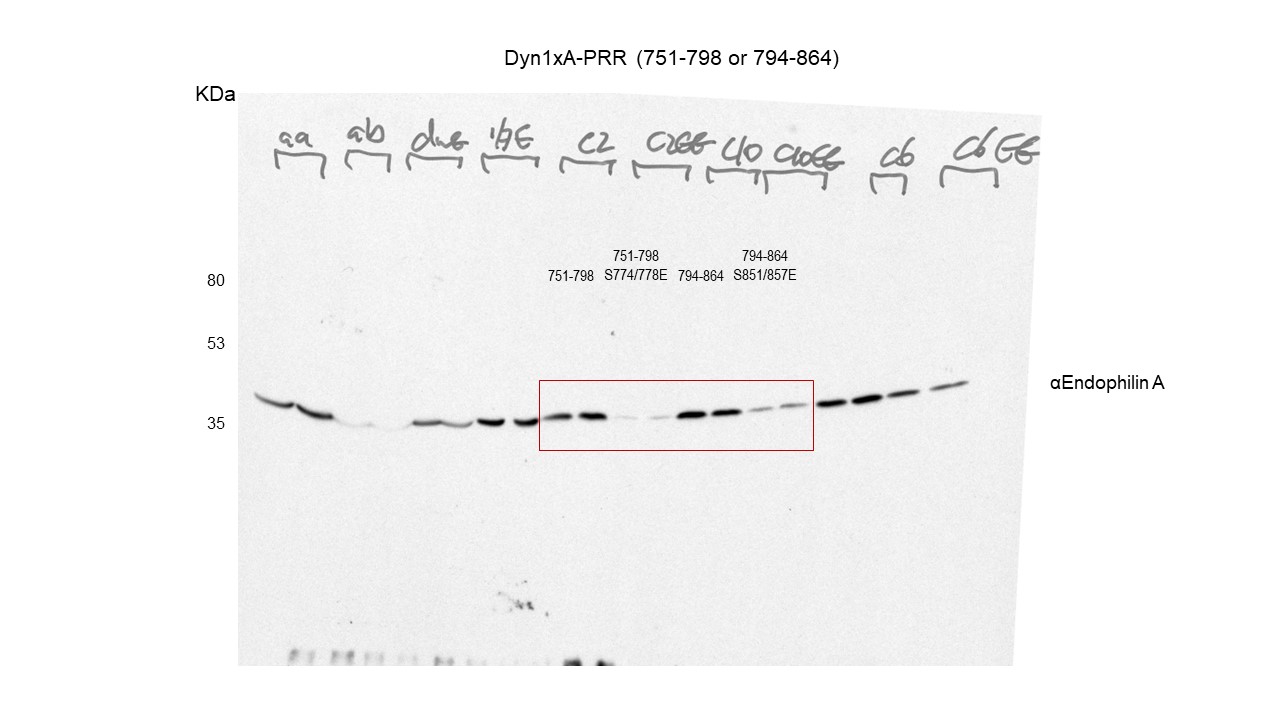

Supplement: Supplementary file 5 — Source data Fig. 3 [file 44318_2024_145_MOESM5_ESM.zip › Source_data_Figure_3/3K/Western Endophilin_right panel.jpg]

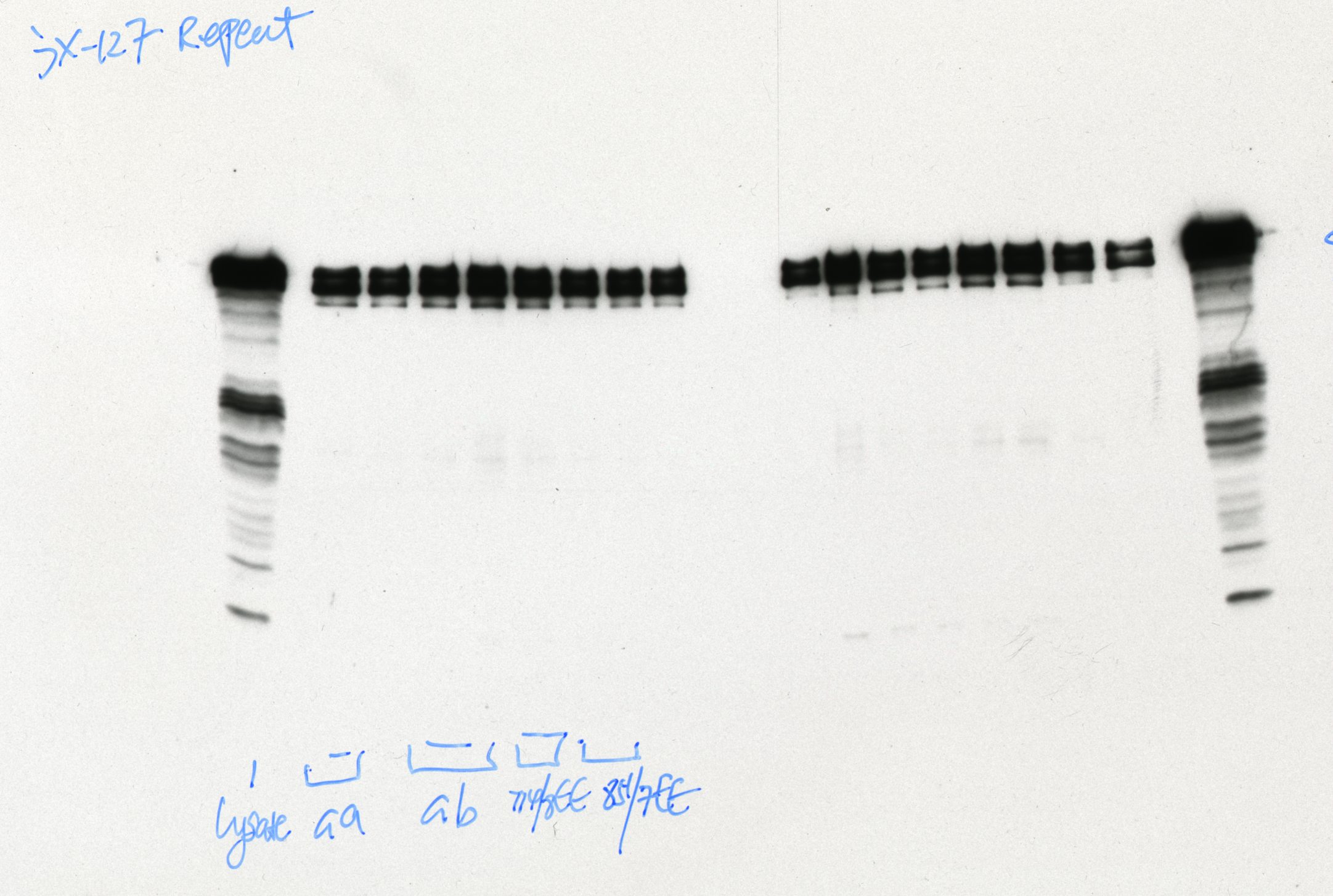

Supplement: Supplementary file 5 — Source data Fig. 3 [file 44318_2024_145_MOESM5_ESM.zip › Source_data_Figure_3/3L and 3M/Original WB Scans for endo binding to Phospho Mutants quantification/jx-127 repeat_Amph.tif]

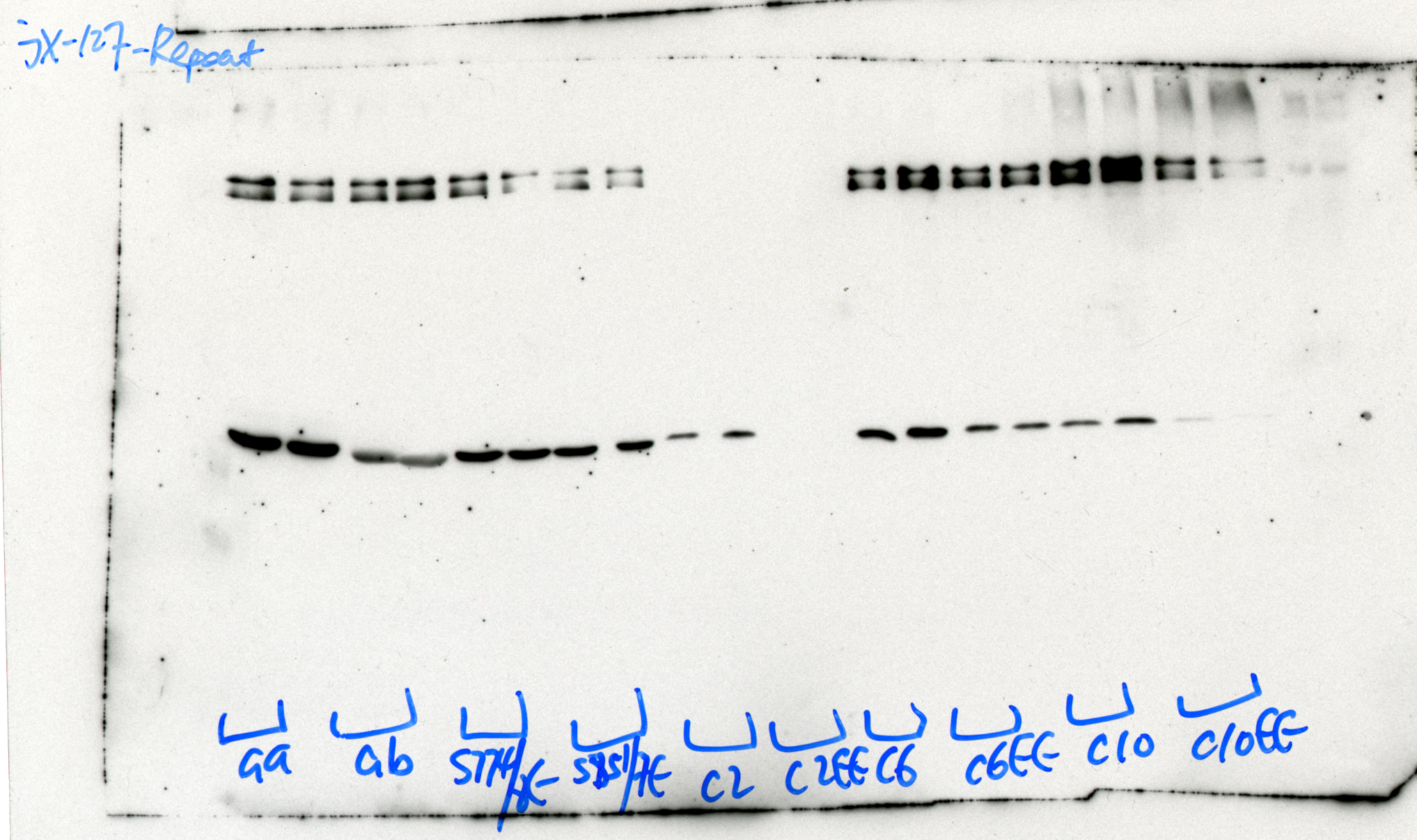

Supplement: Supplementary file 5 — Source data Fig. 3 [file 44318_2024_145_MOESM5_ESM.zip › Source_data_Figure_3/3L and 3M/Original WB Scans for endo binding to Phospho Mutants quantification/jx-127 repeat_Endo.tif]

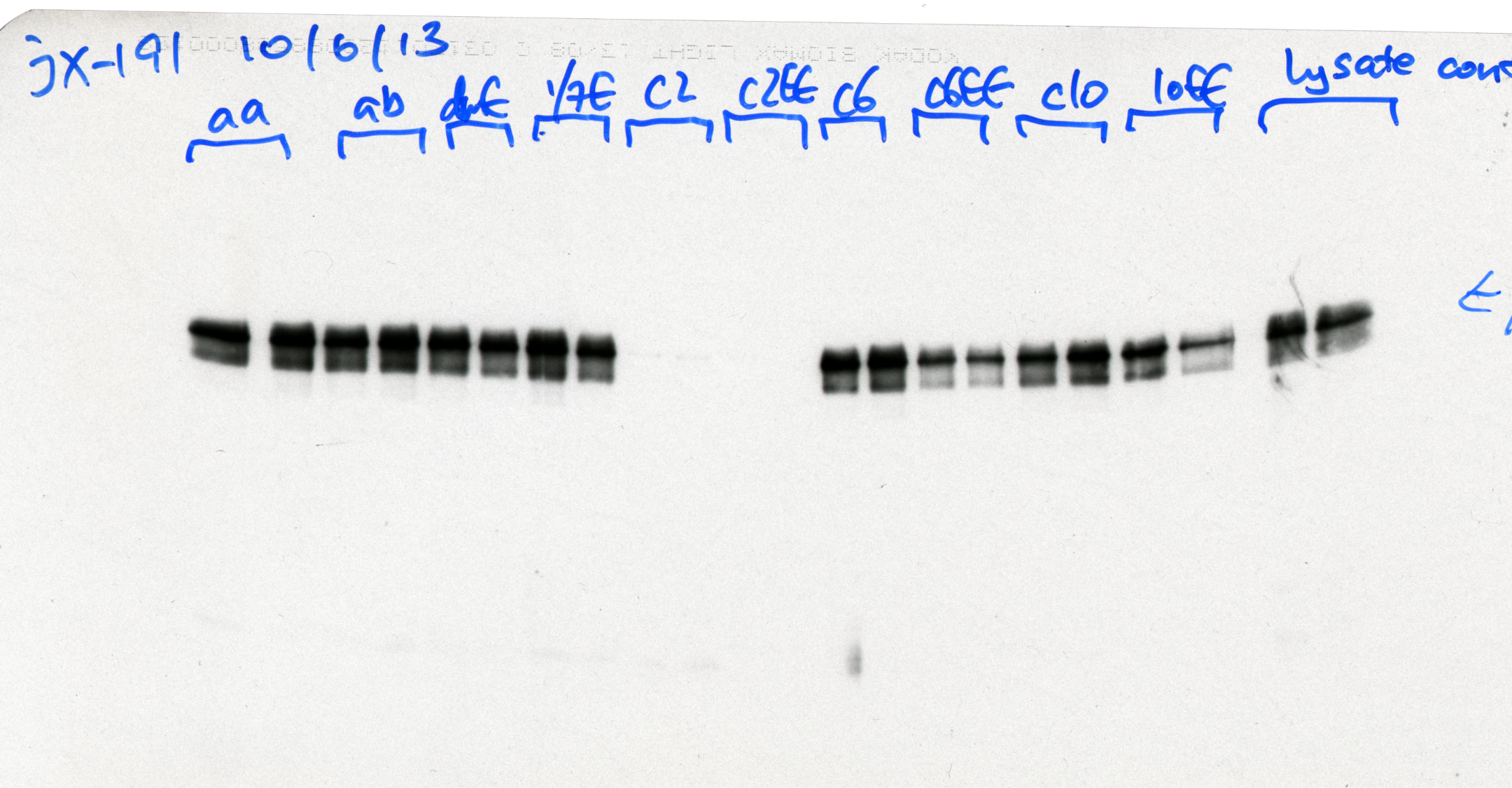

Supplement: Supplementary file 5 — Source data Fig. 3 [file 44318_2024_145_MOESM5_ESM.zip › Source_data_Figure_3/3L and 3M/Original WB Scans for endo binding to Phospho Mutants quantification/jx-191_Amph.tif]

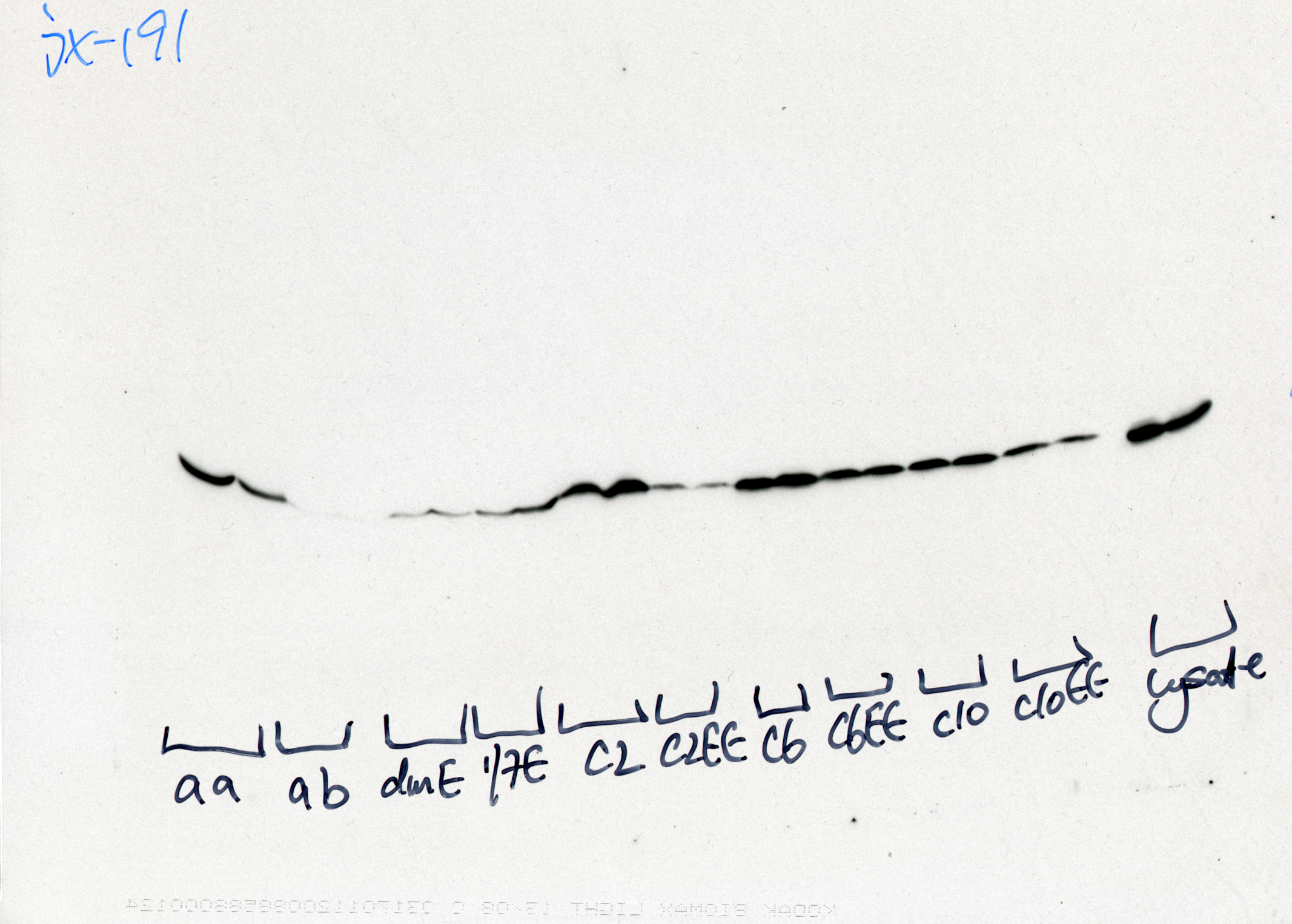

Supplement: Supplementary file 5 — Source data Fig. 3 [file 44318_2024_145_MOESM5_ESM.zip › Source_data_Figure_3/3L and 3M/Original WB Scans for endo binding to Phospho Mutants quantification/jx-191_Endo.tif]

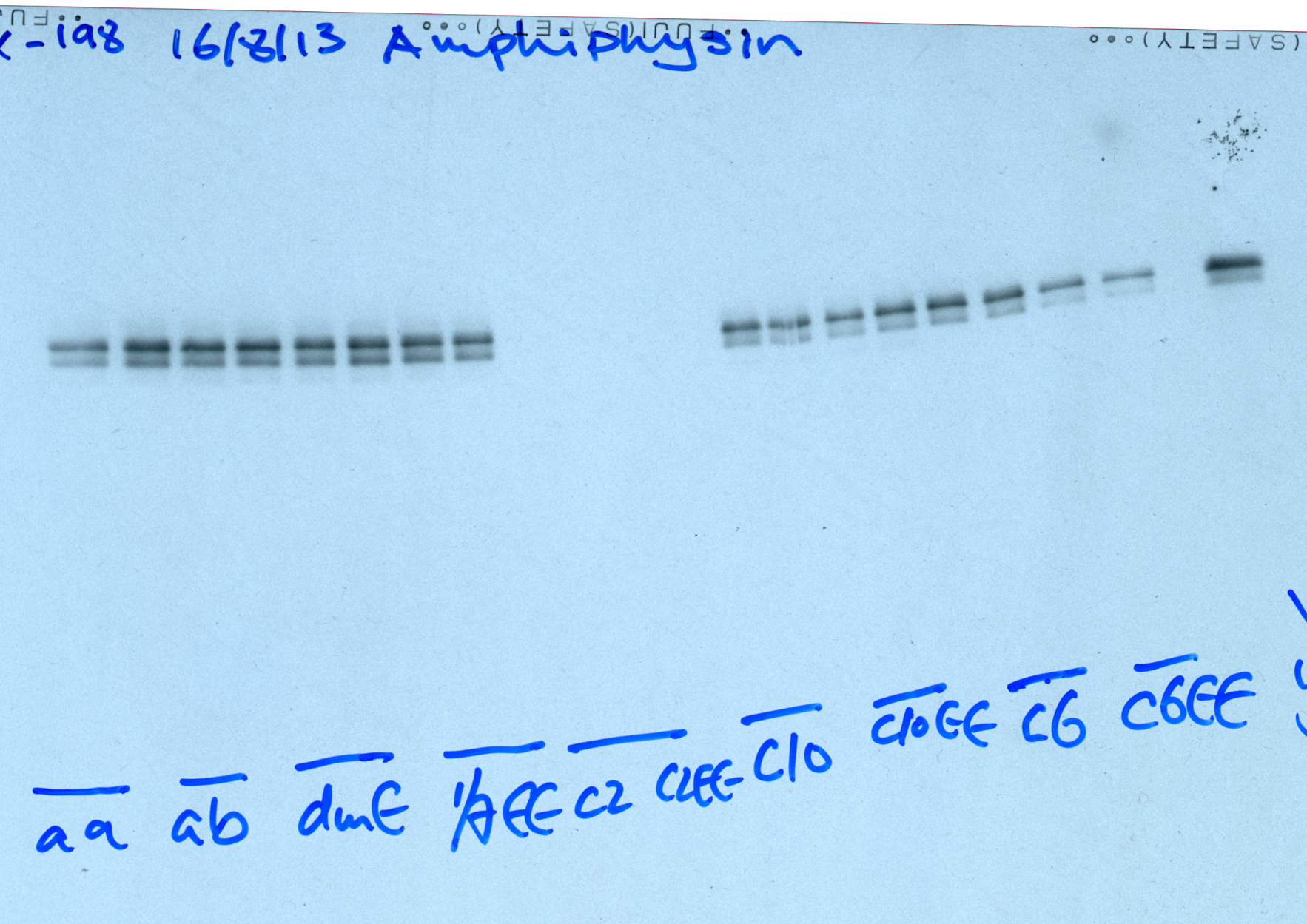

Supplement: Supplementary file 5 — Source data Fig. 3 [file 44318_2024_145_MOESM5_ESM.zip › Source_data_Figure_3/3L and 3M/Original WB Scans for endo binding to Phospho Mutants quantification/jx-198_Amph.tif]

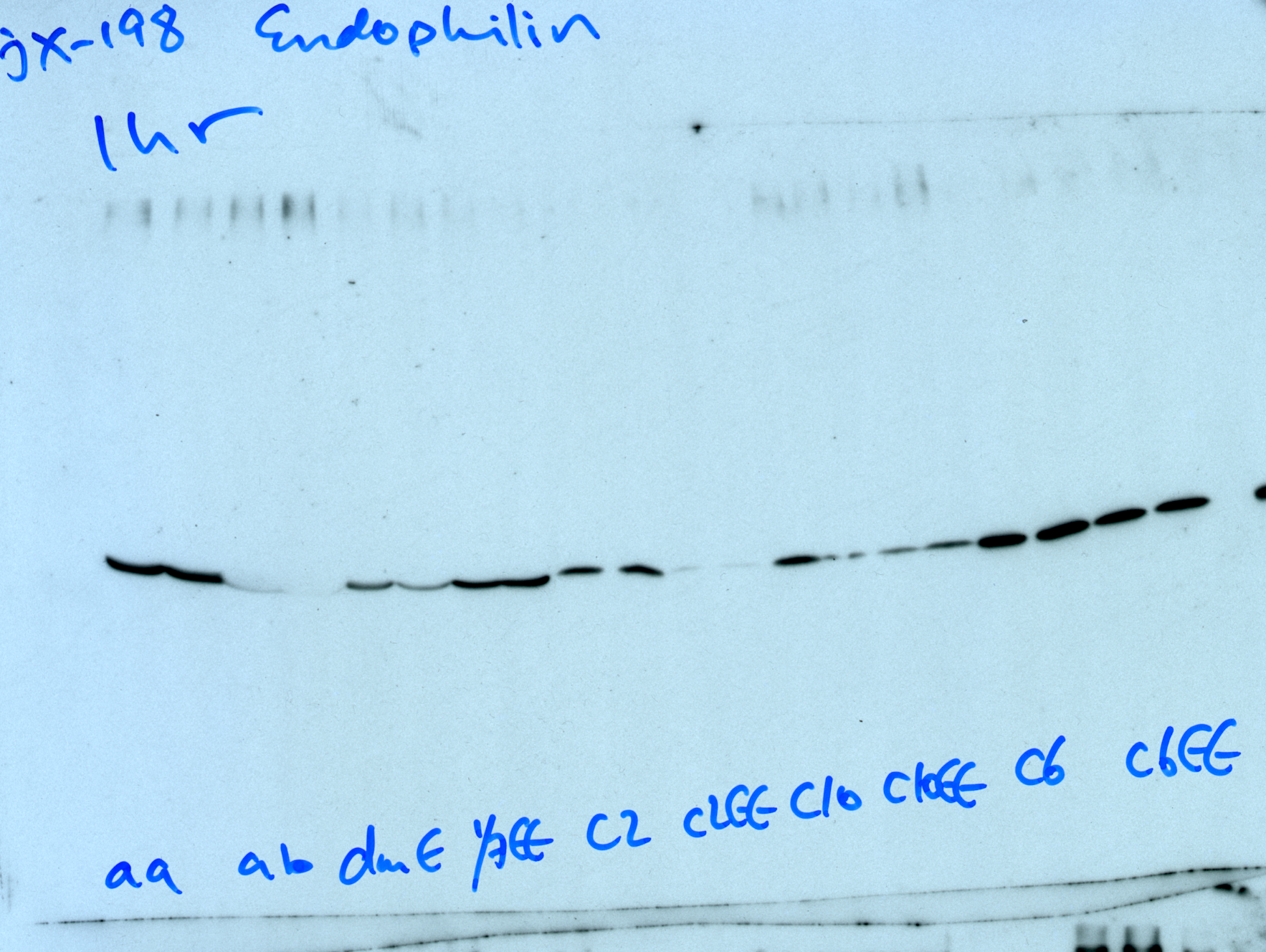

Supplement: Supplementary file 5 — Source data Fig. 3 [file 44318_2024_145_MOESM5_ESM.zip › Source_data_Figure_3/3L and 3M/Original WB Scans for endo binding to Phospho Mutants quantification/jx-198_Endo.tif]

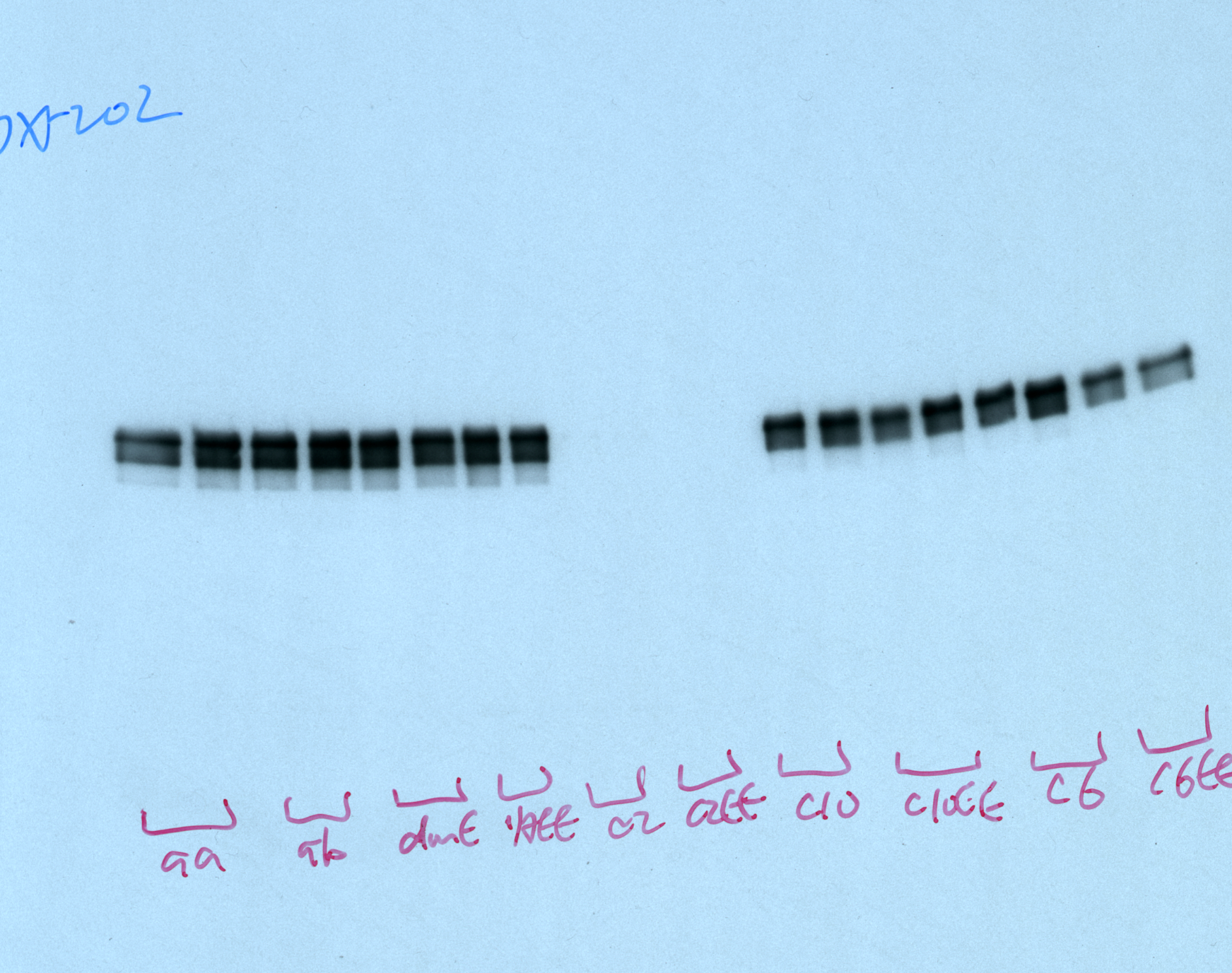

Supplement: Supplementary file 5 — Source data Fig. 3 [file 44318_2024_145_MOESM5_ESM.zip › Source_data_Figure_3/3L and 3M/Original WB Scans for endo binding to Phospho Mutants quantification/jx-202_Amph.tif]

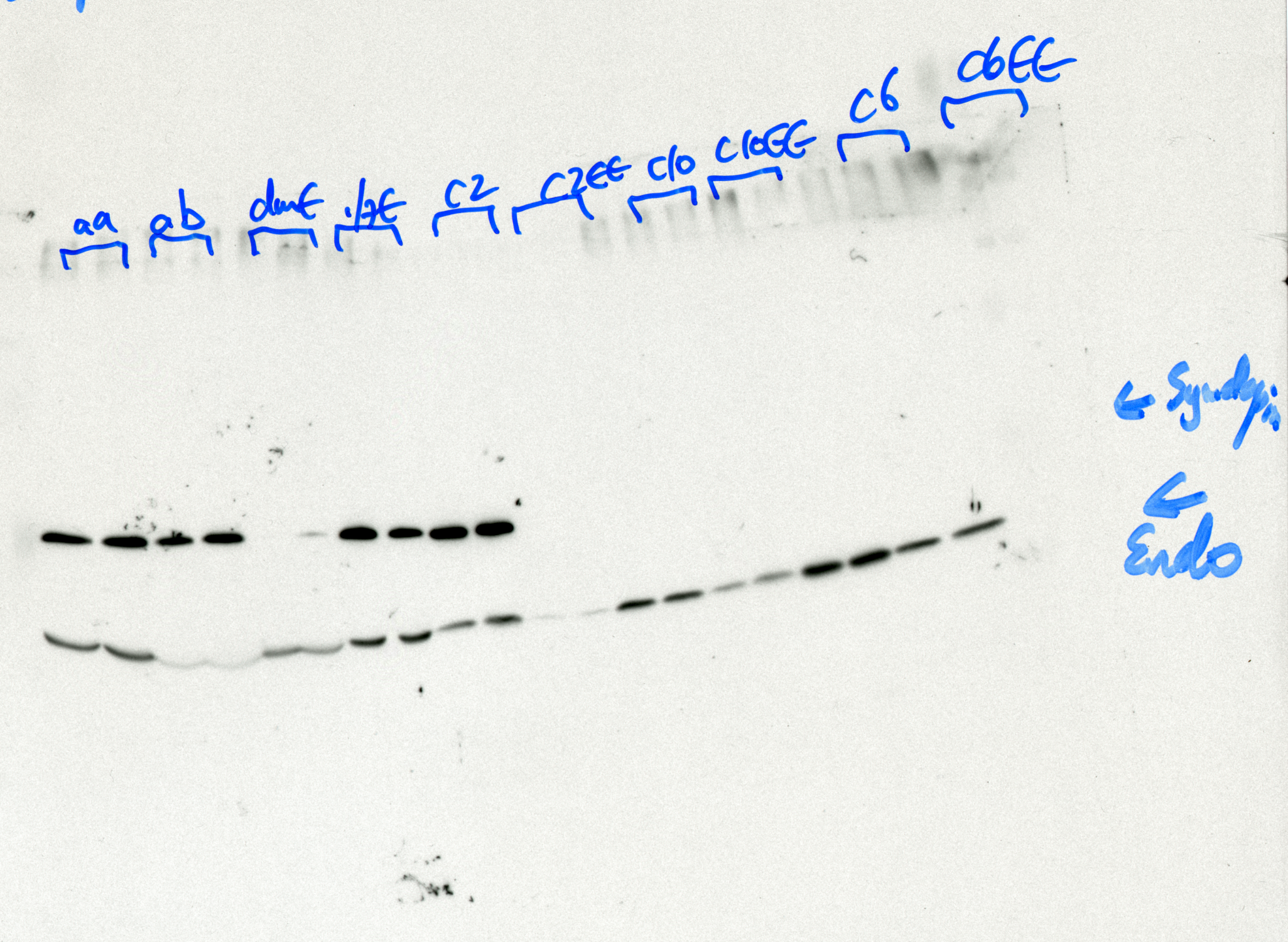

Supplement: Supplementary file 5 — Source data Fig. 3 [file 44318_2024_145_MOESM5_ESM.zip › Source_data_Figure_3/3L and 3M/Original WB Scans for endo binding to Phospho Mutants quantification/jx-202_Endo and Syndapin.tif]

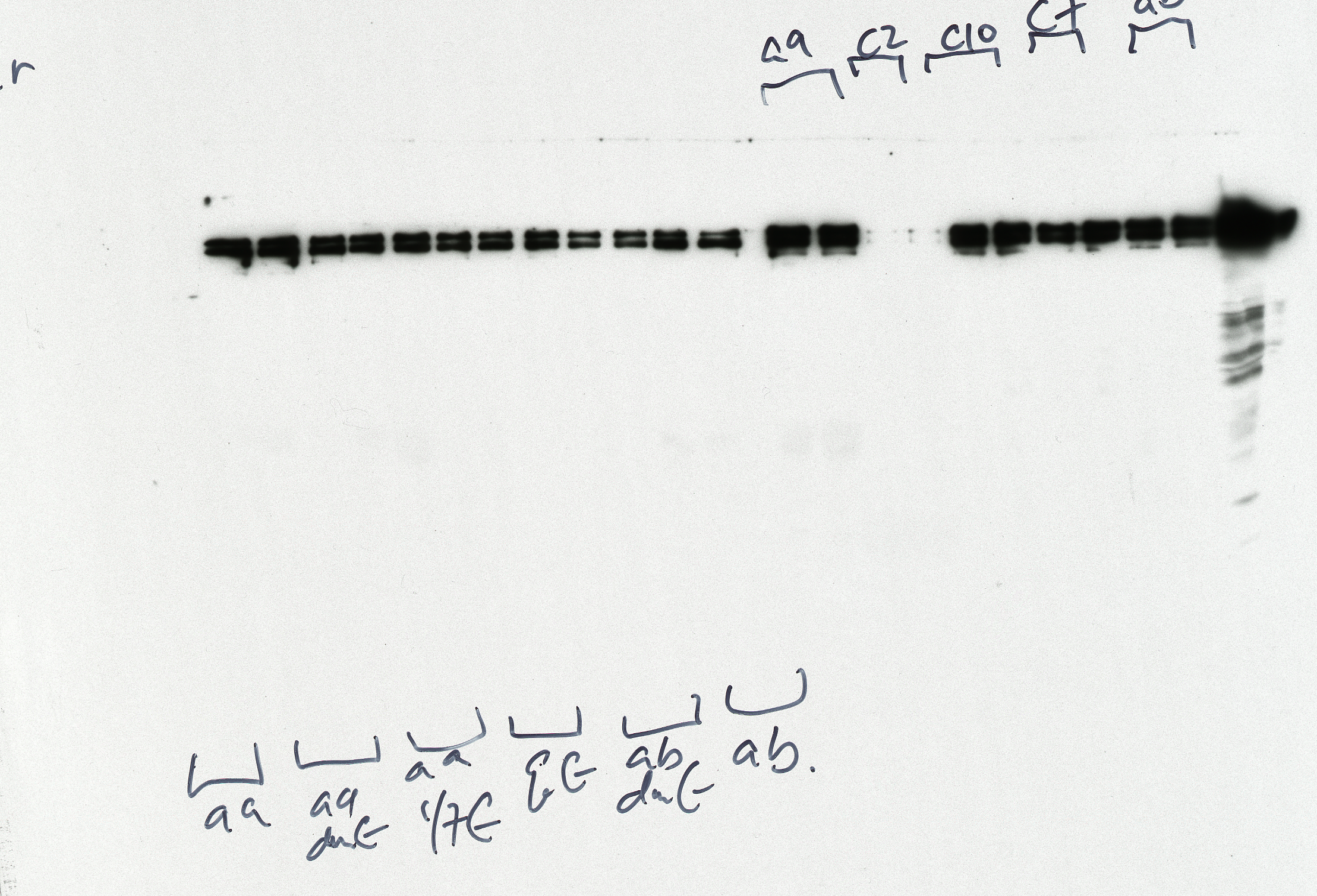

Supplement: Supplementary file 5 — Source data Fig. 3 [file 44318_2024_145_MOESM5_ESM.zip › Source_data_Figure_3/3L and 3M/Original WB Scans for endo binding to Phospho Mutants quantification/JX-238_Amphi.tif]

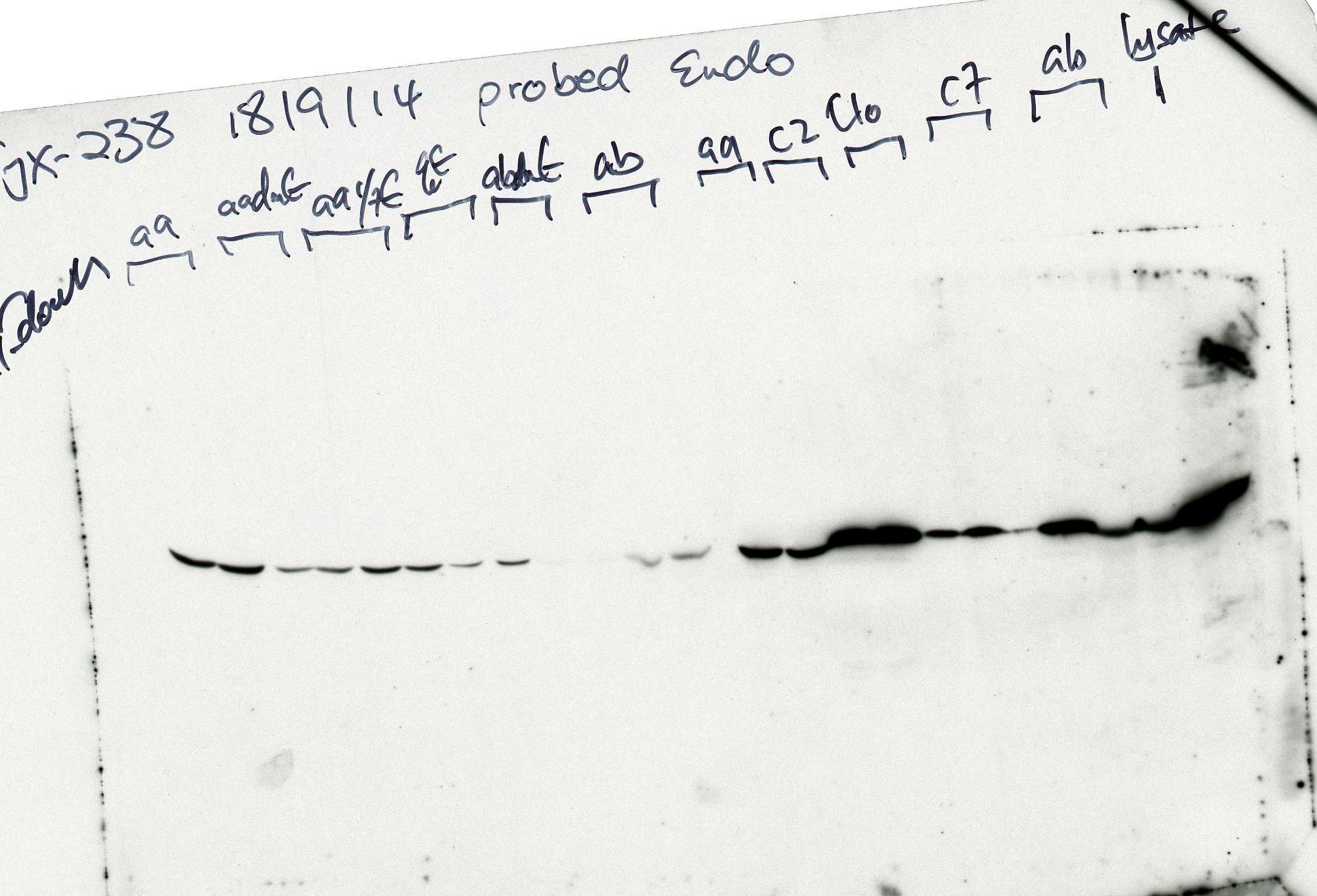

Supplement: Supplementary file 5 — Source data Fig. 3 [file 44318_2024_145_MOESM5_ESM.zip › Source_data_Figure_3/3L and 3M/Original WB Scans for endo binding to Phospho Mutants quantification/JX-238_endophilin.tif]

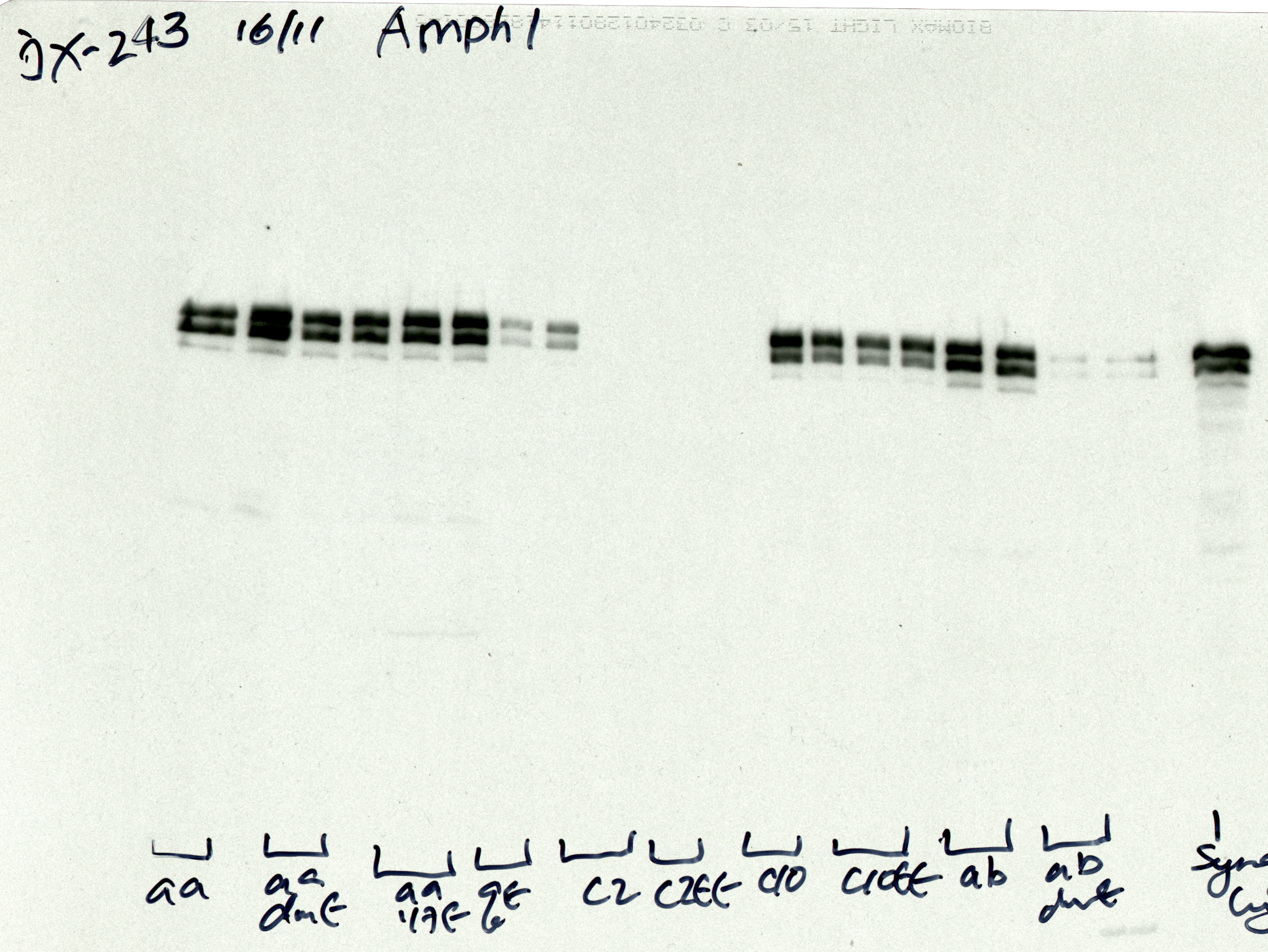

Supplement: Supplementary file 5 — Source data Fig. 3 [file 44318_2024_145_MOESM5_ESM.zip › Source_data_Figure_3/3L and 3M/Original WB Scans for endo binding to Phospho Mutants quantification/jx-243_Amph.tif]

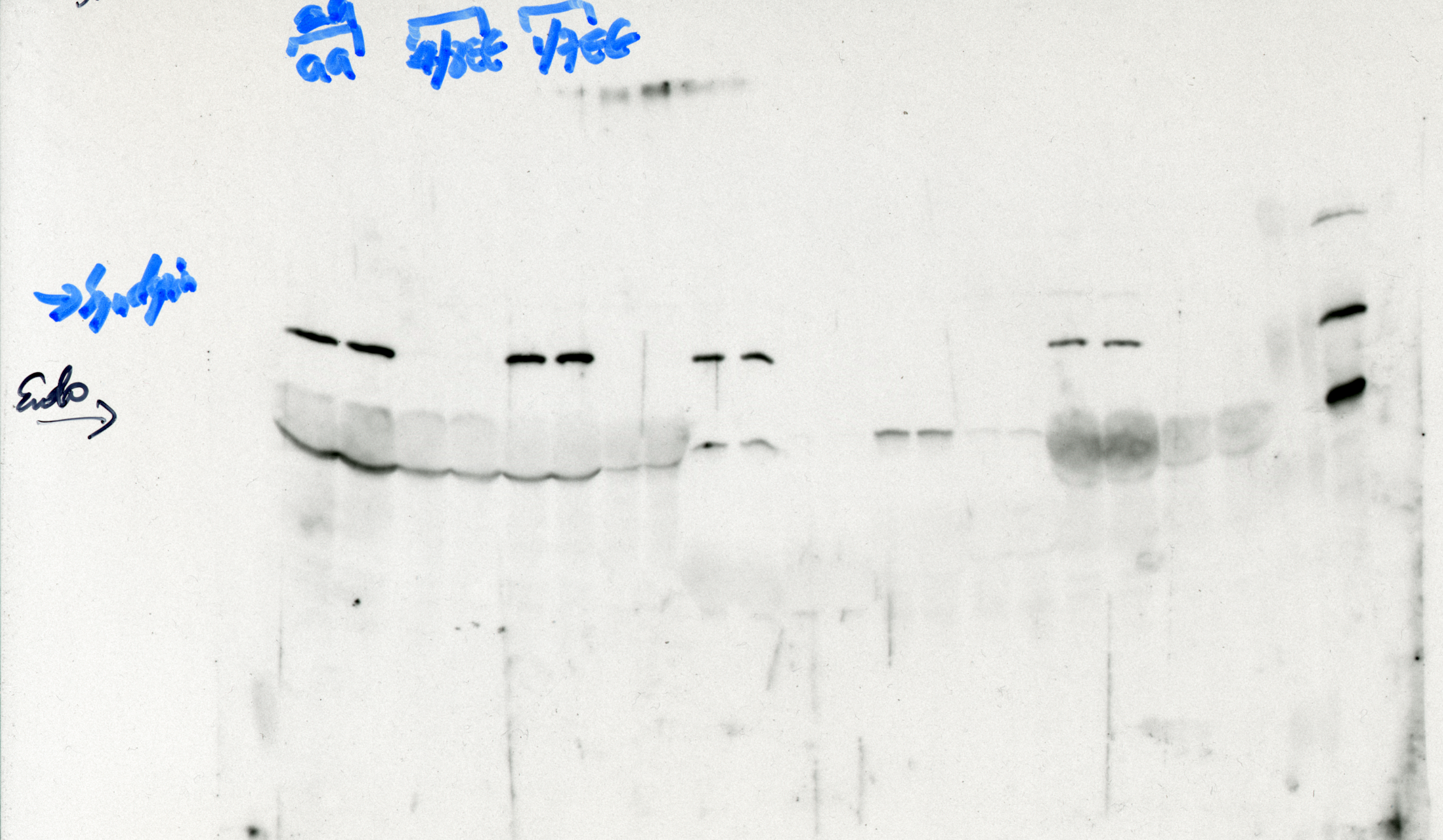

Supplement: Supplementary file 5 — Source data Fig. 3 [file 44318_2024_145_MOESM5_ESM.zip › Source_data_Figure_3/3L and 3M/Original WB Scans for endo binding to Phospho Mutants quantification/jx-243_Endo and syndapin.tif]

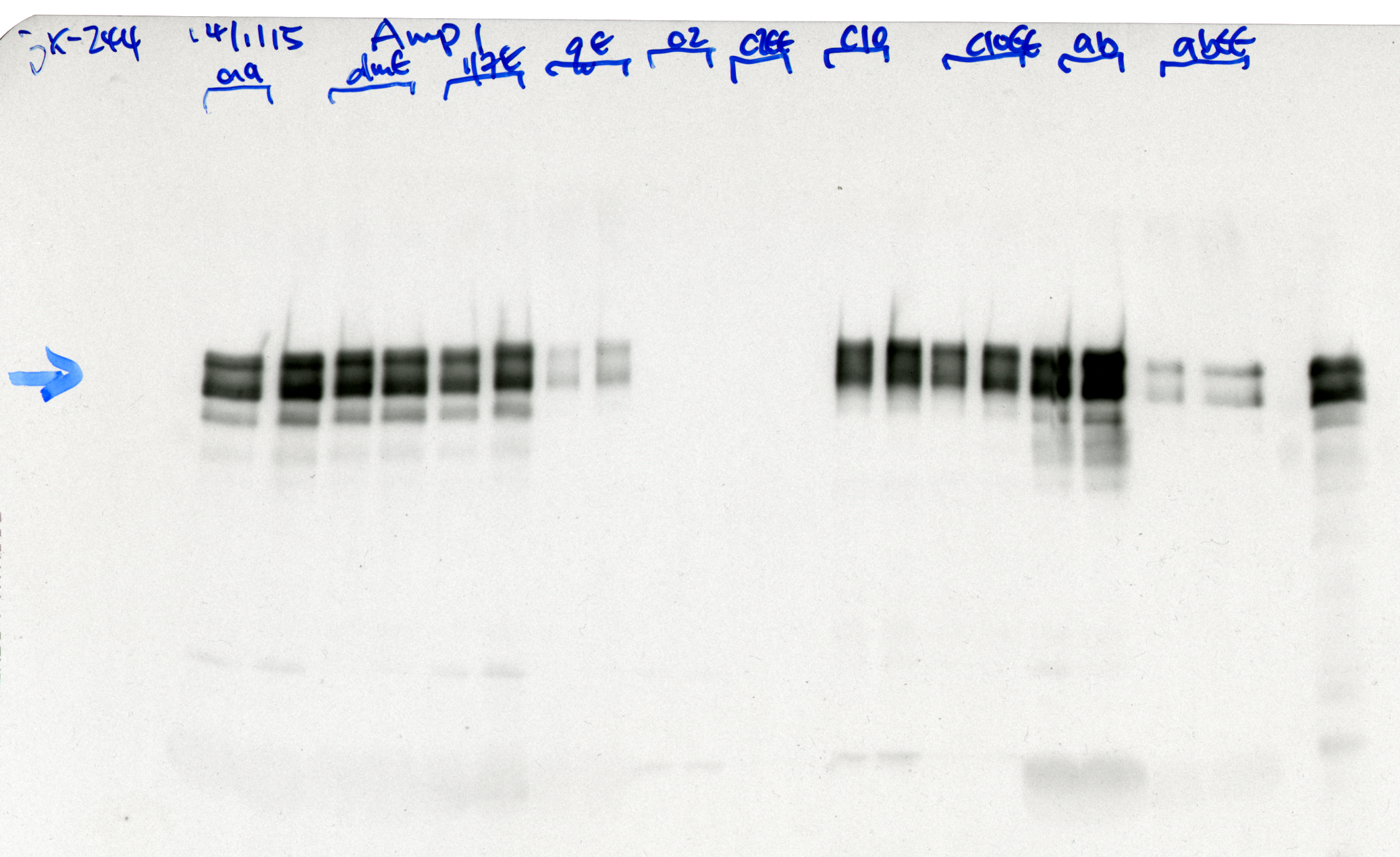

Supplement: Supplementary file 5 — Source data Fig. 3 [file 44318_2024_145_MOESM5_ESM.zip › Source_data_Figure_3/3L and 3M/Original WB Scans for endo binding to Phospho Mutants quantification/jx-244_Amph.tif]

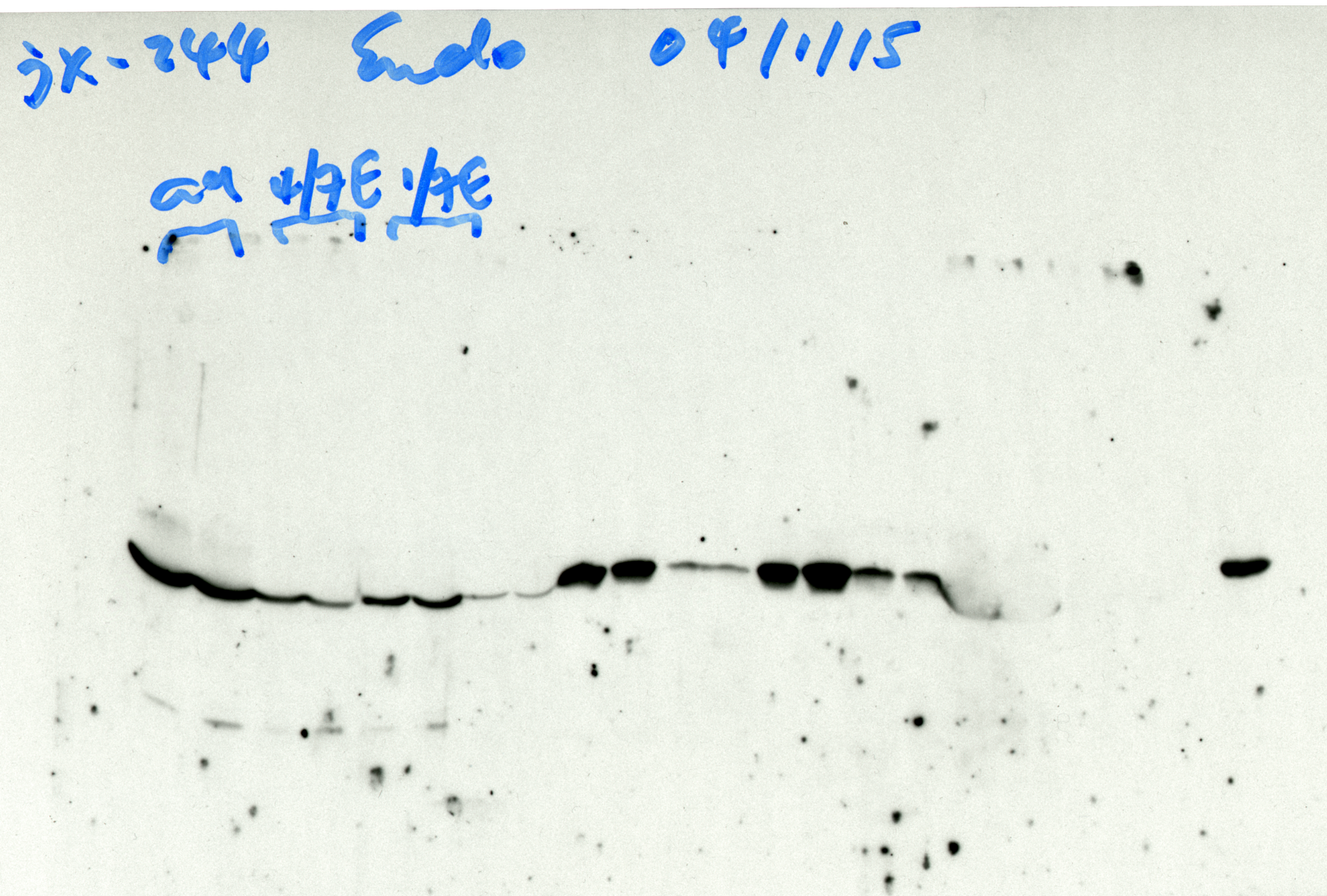

Supplement: Supplementary file 5 — Source data Fig. 3 [file 44318_2024_145_MOESM5_ESM.zip › Source_data_Figure_3/3L and 3M/Original WB Scans for endo binding to Phospho Mutants quantification/jx-244_Endo.tif]

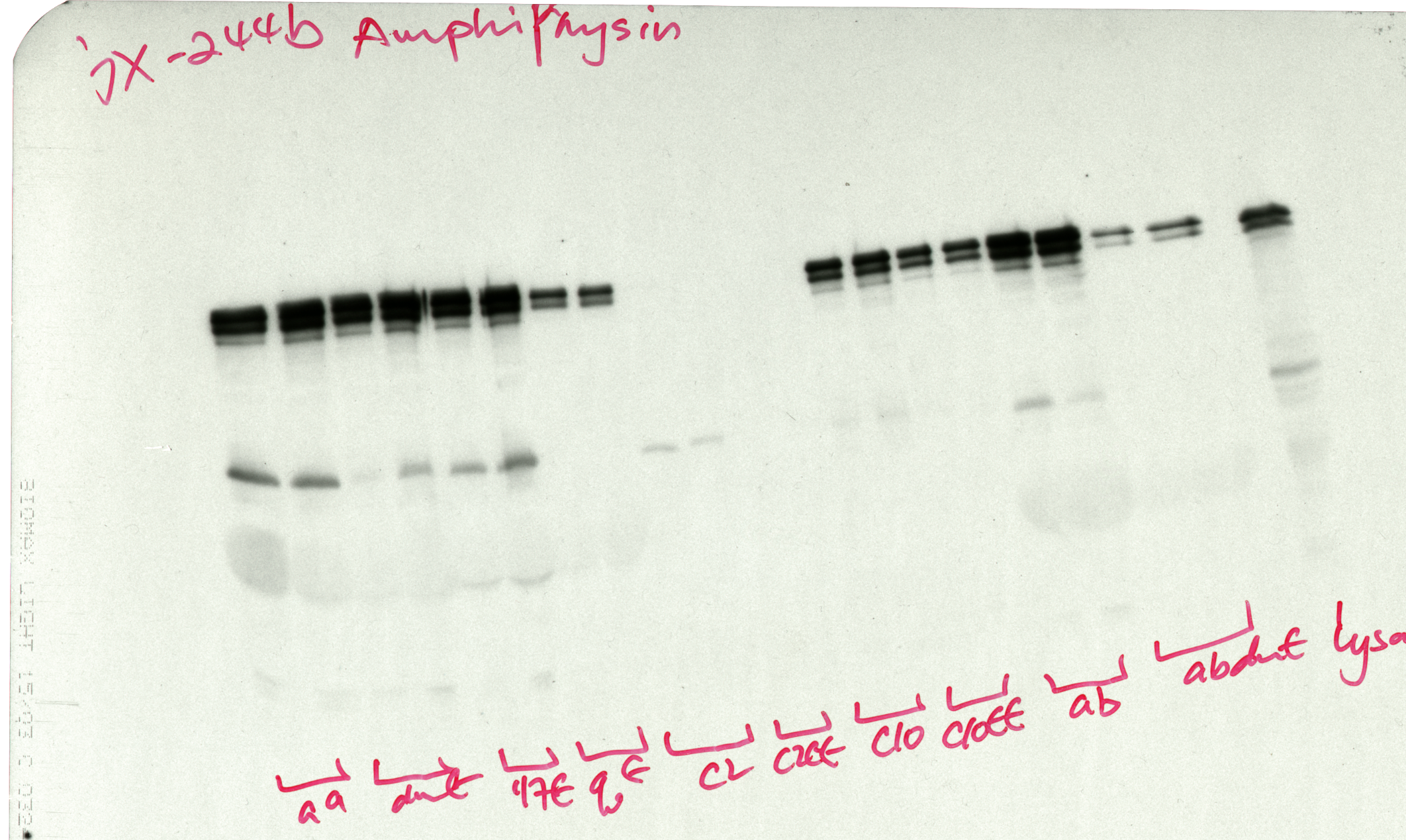

Supplement: Supplementary file 5 — Source data Fig. 3 [file 44318_2024_145_MOESM5_ESM.zip › Source_data_Figure_3/3L and 3M/Original WB Scans for endo binding to Phospho Mutants quantification/jx-244b_Amph.tif]

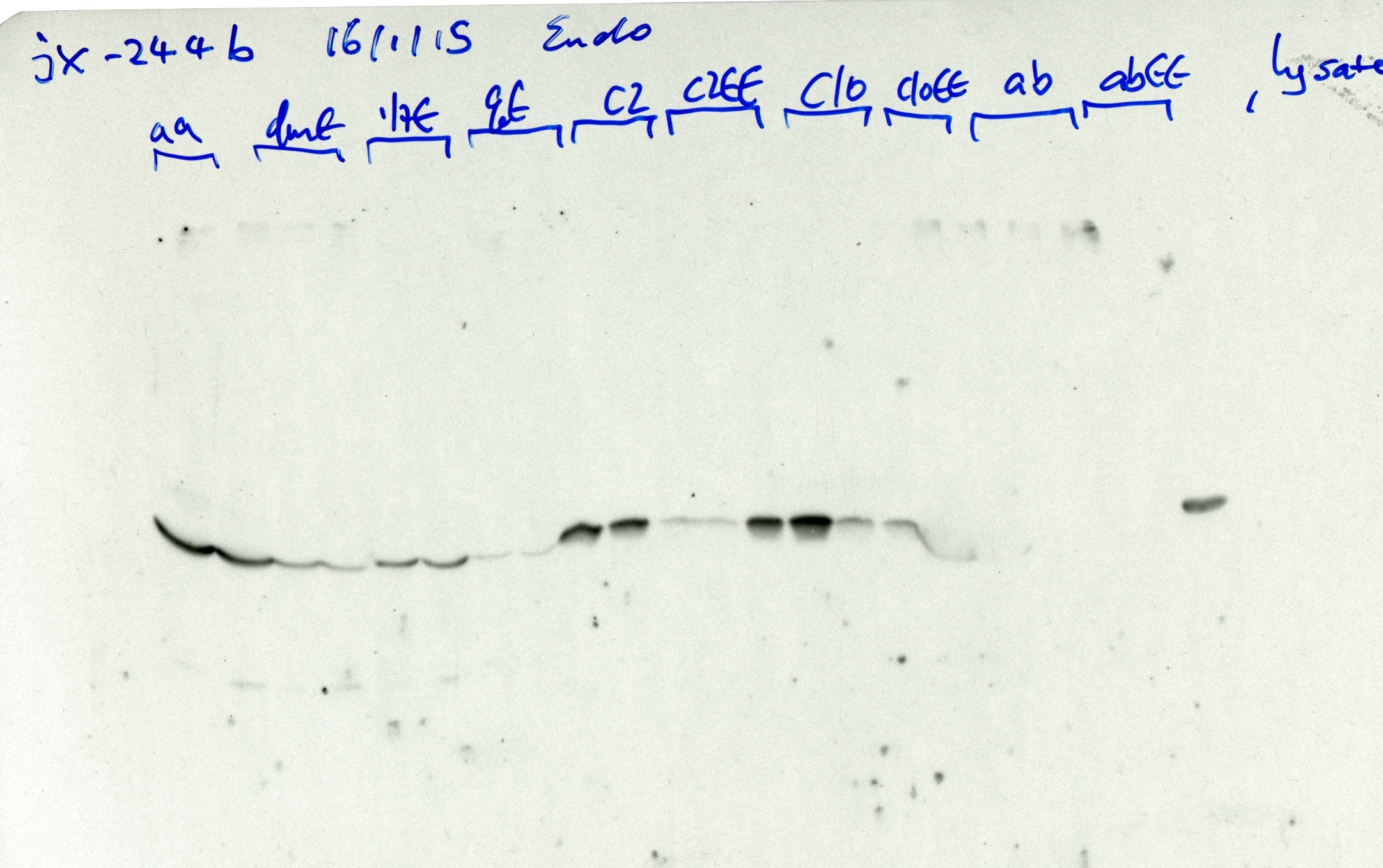

Supplement: Supplementary file 5 — Source data Fig. 3 [file 44318_2024_145_MOESM5_ESM.zip › Source_data_Figure_3/3L and 3M/Original WB Scans for endo binding to Phospho Mutants quantification/jx-244b_Endo.tif]

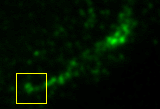

Supplement: Supplementary file 6 — Source data Fig. 4 [file 44318_2024_145_MOESM6_ESM.zip › Source_data_Figure_4/4A/1_Dyn1xA-GFP-AF594.tif]

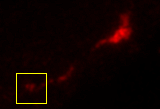

Supplement: Supplementary file 6 — Source data Fig. 4 [file 44318_2024_145_MOESM6_ESM.zip › Source_data_Figure_4/4A/1_EndoA1-mC-Atto647.tif]

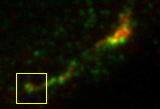

Supplement: Supplementary file 6 — Source data Fig. 4 [file 44318_2024_145_MOESM6_ESM.zip › Source_data_Figure_4/4A/1_Merged.tif]

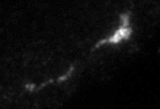

Supplement: Supplementary file 6 — Source data Fig. 4 [file 44318_2024_145_MOESM6_ESM.zip › Source_data_Figure_4/4A/1_Original_image_Decon_Dyn1xA-GFP-AF594_EndoA1-mC-Atto647_STED.tif]

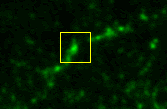

Supplement: Supplementary file 6 — Source data Fig. 4 [file 44318_2024_145_MOESM6_ESM.zip › Source_data_Figure_4/4A/2_Dyn1xA-GFP-AF594.tif]

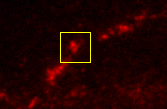

Supplement: Supplementary file 6 — Source data Fig. 4 [file 44318_2024_145_MOESM6_ESM.zip › Source_data_Figure_4/4A/2_EndoA1-mC-Atto647.tif]

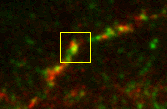

Supplement: Supplementary file 6 — Source data Fig. 4 [file 44318_2024_145_MOESM6_ESM.zip › Source_data_Figure_4/4A/2_Merged.tif]

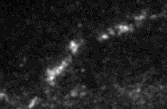

Supplement: Supplementary file 6 — Source data Fig. 4 [file 44318_2024_145_MOESM6_ESM.zip › Source_data_Figure_4/4A/2_Original_image_Decon_Dyn1xA-GFP-AF594_EndoA1-mC-Atto647_STED.tif]

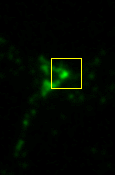

Supplement: Supplementary file 6 — Source data Fig. 4 [file 44318_2024_145_MOESM6_ESM.zip › Source_data_Figure_4/4A/3_Dyn1xA-GFP-AF594.tif]

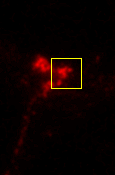

Supplement: Supplementary file 6 — Source data Fig. 4 [file 44318_2024_145_MOESM6_ESM.zip › Source_data_Figure_4/4A/3_EndoA1-mC-Atto647.tif]

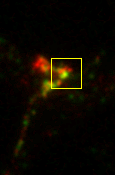

Supplement: Supplementary file 6 — Source data Fig. 4 [file 44318_2024_145_MOESM6_ESM.zip › Source_data_Figure_4/4A/3_Merged.tif]

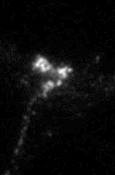

Supplement: Supplementary file 6 — Source data Fig. 4 [file 44318_2024_145_MOESM6_ESM.zip › Source_data_Figure_4/4A/3_Original_image_Decon_Dyn1xA-GFP-AF594_EndoA1-mC-Atto647_STED.tif]

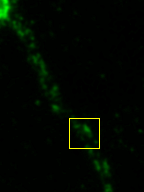

Supplement: Supplementary file 6 — Source data Fig. 4 [file 44318_2024_145_MOESM6_ESM.zip › Source_data_Figure_4/4B/1_Dyn1xA-mC-Atto647.tif]

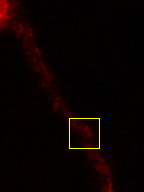

Supplement: Supplementary file 6 — Source data Fig. 4 [file 44318_2024_145_MOESM6_ESM.zip › Source_data_Figure_4/4B/1_EndoA2-GFP-AF594.tif]

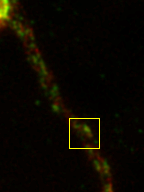

Supplement: Supplementary file 6 — Source data Fig. 4 [file 44318_2024_145_MOESM6_ESM.zip › Source_data_Figure_4/4B/1_Merged.tif]

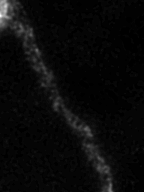

Supplement: Supplementary file 6 — Source data Fig. 4 [file 44318_2024_145_MOESM6_ESM.zip › Source_data_Figure_4/4B/1_Original_image_Decon_Dyn1xA-mC-Atto647_EndoA2-GFP-AF594_STED.tif]

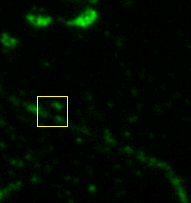

Supplement: Supplementary file 6 — Source data Fig. 4 [file 44318_2024_145_MOESM6_ESM.zip › Source_data_Figure_4/4B/2_Dyn1xA-mC-Atto647.tif]

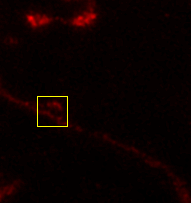

Supplement: Supplementary file 6 — Source data Fig. 4 [file 44318_2024_145_MOESM6_ESM.zip › Source_data_Figure_4/4B/2_EndoA2-GFP-AF594.tif]

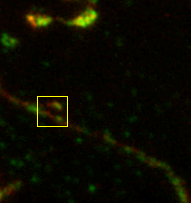

Supplement: Supplementary file 6 — Source data Fig. 4 [file 44318_2024_145_MOESM6_ESM.zip › Source_data_Figure_4/4B/2_Merged.tif]

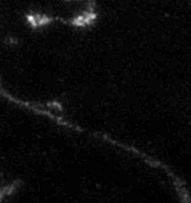

Supplement: Supplementary file 6 — Source data Fig. 4 [file 44318_2024_145_MOESM6_ESM.zip › Source_data_Figure_4/4B/2_Original_image_Decon_Dyn1xA-mC-Atto647_EndoA2-GFP-AF594_STED.tif]

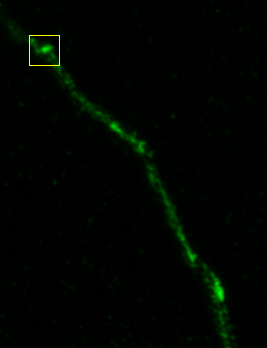

Supplement: Supplementary file 6 — Source data Fig. 4 [file 44318_2024_145_MOESM6_ESM.zip › Source_data_Figure_4/4B/3_Dyn1xA-mC-Atto647.tif]

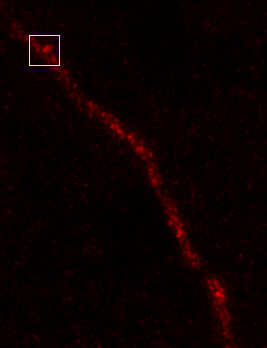

Supplement: Supplementary file 6 — Source data Fig. 4 [file 44318_2024_145_MOESM6_ESM.zip › Source_data_Figure_4/4B/3_EndoA2-GFP-AF594.tif]

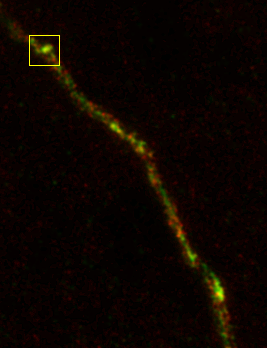

Supplement: Supplementary file 6 — Source data Fig. 4 [file 44318_2024_145_MOESM6_ESM.zip › Source_data_Figure_4/4B/3_Merged.tif]

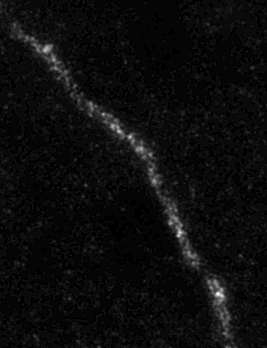

Supplement: Supplementary file 6 — Source data Fig. 4 [file 44318_2024_145_MOESM6_ESM.zip › Source_data_Figure_4/4B/3_Original_image_Decon_Dyn1xA-mC-Atto647_EndoA2-GFP-AF594_STED.tif]

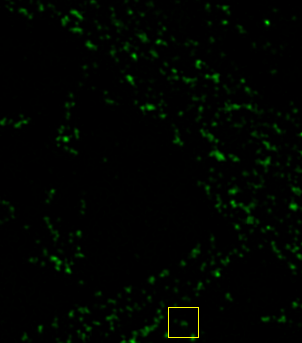

Supplement: Supplementary file 6 — Source data Fig. 4 [file 44318_2024_145_MOESM6_ESM.zip › Source_data_Figure_4/4E/Dyn1xA/1_aBassoon-AF594.tif]

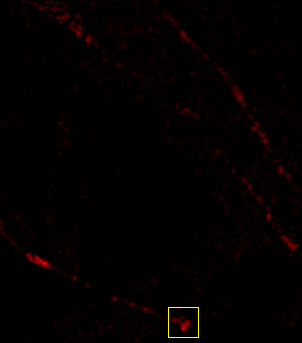

Supplement: Supplementary file 6 — Source data Fig. 4 [file 44318_2024_145_MOESM6_ESM.zip › Source_data_Figure_4/4E/Dyn1xA/1_Dyn1xA-GFP-Atto647.tif]

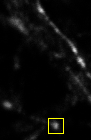

Supplement: Supplementary file 6 — Source data Fig. 4 [file 44318_2024_145_MOESM6_ESM.zip › Source_data_Figure_4/4E/Dyn1xA/1_Dyn1xA-GFP-Atto647_CONFOCAL.tif]

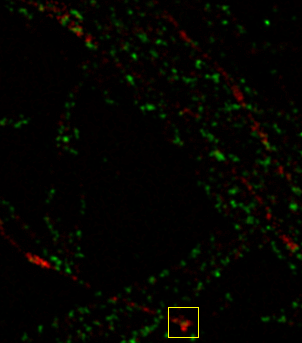

Supplement: Supplementary file 6 — Source data Fig. 4 [file 44318_2024_145_MOESM6_ESM.zip › Source_data_Figure_4/4E/Dyn1xA/1_Merged.tif]

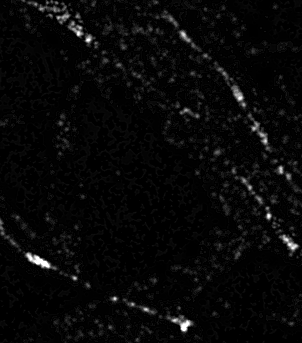

Supplement: Supplementary file 6 — Source data Fig. 4 [file 44318_2024_145_MOESM6_ESM.zip › Source_data_Figure_4/4E/Dyn1xA/1_Original_image_Decon_Dyn1xA-GFP-Atto647_aBassoon-AF594_STED.tif]

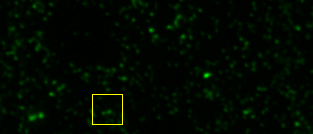

Supplement: Supplementary file 6 — Source data Fig. 4 [file 44318_2024_145_MOESM6_ESM.zip › Source_data_Figure_4/4E/Dyn1xA/2_aBassoon-AF594.tif]

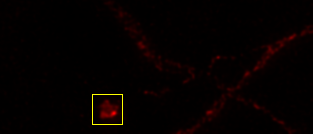

Supplement: Supplementary file 6 — Source data Fig. 4 [file 44318_2024_145_MOESM6_ESM.zip › Source_data_Figure_4/4E/Dyn1xA/2_Dyn1xA-GFP-Atto647.tif]

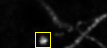

Supplement: Supplementary file 6 — Source data Fig. 4 [file 44318_2024_145_MOESM6_ESM.zip › Source_data_Figure_4/4E/Dyn1xA/2_Dyn1xA-GFP-Atto647_CONFOCAL.tif]

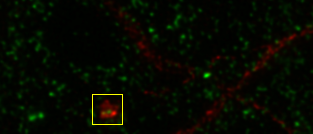

Supplement: Supplementary file 6 — Source data Fig. 4 [file 44318_2024_145_MOESM6_ESM.zip › Source_data_Figure_4/4E/Dyn1xA/2_Merged.tif]

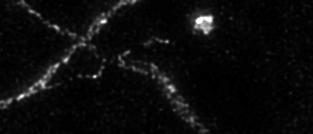

Supplement: Supplementary file 6 — Source data Fig. 4 [file 44318_2024_145_MOESM6_ESM.zip › Source_data_Figure_4/4E/Dyn1xA/2_Original_image_Decon_Dyn1xA-GFP-Atto647_aBassoon-AF594_STED.tif]

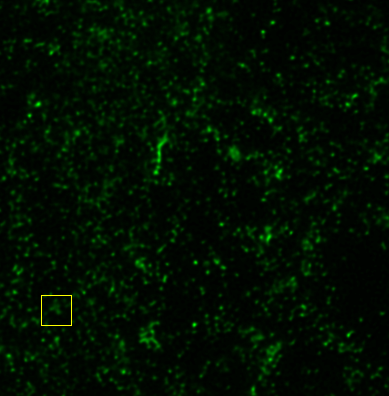

Supplement: Supplementary file 6 — Source data Fig. 4 [file 44318_2024_145_MOESM6_ESM.zip › Source_data_Figure_4/4E/Dyn1xA/3_aBassoon-AF594.tif]

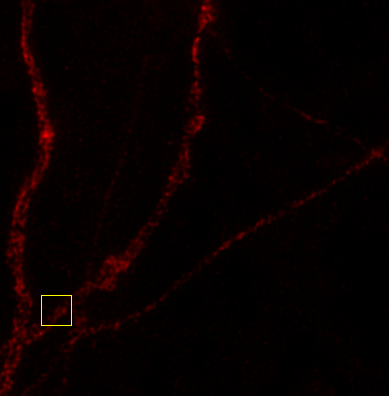

Supplement: Supplementary file 6 — Source data Fig. 4 [file 44318_2024_145_MOESM6_ESM.zip › Source_data_Figure_4/4E/Dyn1xA/3_Dyn1xA-GFP-Atto647.tif]

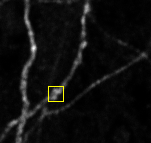

Supplement: Supplementary file 6 — Source data Fig. 4 [file 44318_2024_145_MOESM6_ESM.zip › Source_data_Figure_4/4E/Dyn1xA/3_Dyn1xA-GFP-Atto647_CONFOCAL.tif]

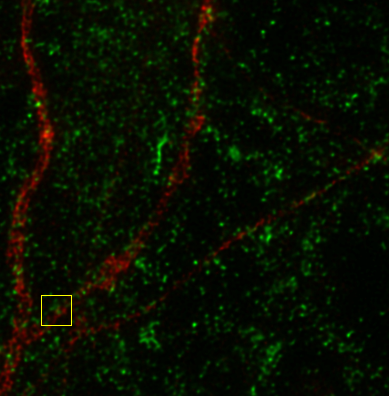

Supplement: Supplementary file 6 — Source data Fig. 4 [file 44318_2024_145_MOESM6_ESM.zip › Source_data_Figure_4/4E/Dyn1xA/3_Merged.tif]

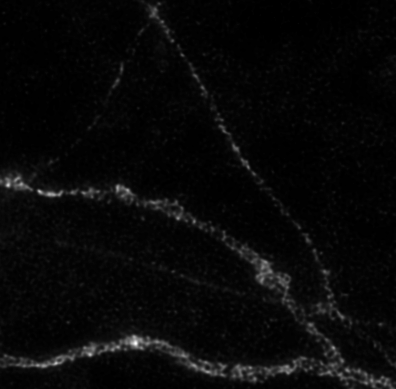

Supplement: Supplementary file 6 — Source data Fig. 4 [file 44318_2024_145_MOESM6_ESM.zip › Source_data_Figure_4/4E/Dyn1xA/3_Original_image_Decon_Dyn1xA-GFP-Atto647_aBassoon-AF594_STED.tif]

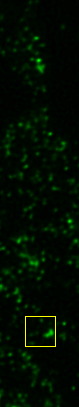

Supplement: Supplementary file 6 — Source data Fig. 4 [file 44318_2024_145_MOESM6_ESM.zip › Source_data_Figure_4/4E/Dyn1xA-R846A/1_aBassoon-AF594.tif]

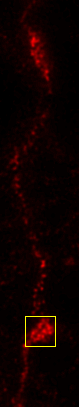

Supplement: Supplementary file 6 — Source data Fig. 4 [file 44318_2024_145_MOESM6_ESM.zip › Source_data_Figure_4/4E/Dyn1xA-R846A/1_Dyn1xA-R846A-GFP-Atto647.tif]

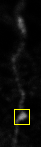

Supplement: Supplementary file 6 — Source data Fig. 4 [file 44318_2024_145_MOESM6_ESM.zip › Source_data_Figure_4/4E/Dyn1xA-R846A/1_Dyn1xA-R846A-GFP-Atto647_CONFOCAL.tif]

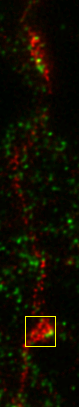

Supplement: Supplementary file 6 — Source data Fig. 4 [file 44318_2024_145_MOESM6_ESM.zip › Source_data_Figure_4/4E/Dyn1xA-R846A/1_Merged.tif]

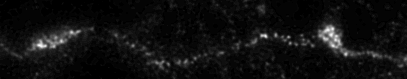

Supplement: Supplementary file 6 — Source data Fig. 4 [file 44318_2024_145_MOESM6_ESM.zip › Source_data_Figure_4/4E/Dyn1xA-R846A/1_Original_image_Decon_Dyn1xA-R846A-GFP-Atto647_aBassoon-AF594_STED.tif]

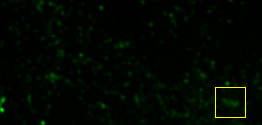

Supplement: Supplementary file 6 — Source data Fig. 4 [file 44318_2024_145_MOESM6_ESM.zip › Source_data_Figure_4/4E/Dyn1xA-R846A/2_aBassoon-AF594.tif]

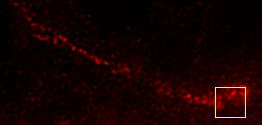

Supplement: Supplementary file 6 — Source data Fig. 4 [file 44318_2024_145_MOESM6_ESM.zip › Source_data_Figure_4/4E/Dyn1xA-R846A/2_Dyn1xA-R846A-GFP-Atto647.tif]

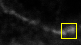

Supplement: Supplementary file 6 — Source data Fig. 4 [file 44318_2024_145_MOESM6_ESM.zip › Source_data_Figure_4/4E/Dyn1xA-R846A/2_Dyn1xA-R846A-GFP-Atto647_CONFOCAL.tif]

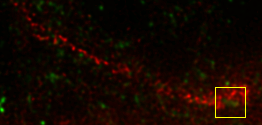

Supplement: Supplementary file 6 — Source data Fig. 4 [file 44318_2024_145_MOESM6_ESM.zip › Source_data_Figure_4/4E/Dyn1xA-R846A/2_Merged.tif]
