# Supplementary figures and images for: Dynamin 1xA interacts with Endophilin A1 via its spliced long C-terminus for ultrafast endocytosis (part 2 of 2)
Source: EMBO J. 2024 Jun 21;43(16):3327–57. doi: 10.1038/s44318-024-00145-x (PMC11329700; doi:10.1038/s44318-024-00145-x)

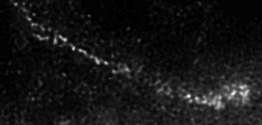

Supplement: Supplementary file 6 — Source data Fig. 4 [file 44318_2024_145_MOESM6_ESM.zip › Source_data_Figure_4/4E/Dyn1xA-R846A/2_Original_image_Decon_Dyn1xA-R846A-GFP-Atto647_aBassoon-AF594_STED.tif]

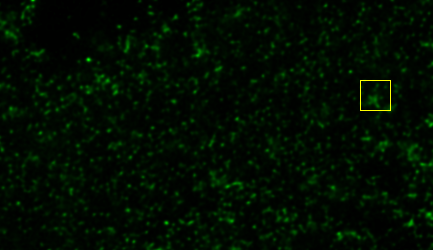

Supplement: Supplementary file 6 — Source data Fig. 4 [file 44318_2024_145_MOESM6_ESM.zip › Source_data_Figure_4/4E/Dyn1xA-R846A/3_aBassoon-AF594.tif]

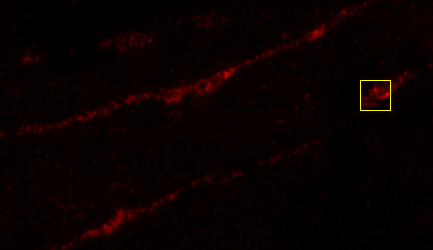

Supplement: Supplementary file 6 — Source data Fig. 4 [file 44318_2024_145_MOESM6_ESM.zip › Source_data_Figure_4/4E/Dyn1xA-R846A/3_Dyn1xA-R846A-GFP-Atto647.tif]

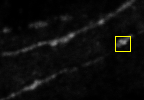

Supplement: Supplementary file 6 — Source data Fig. 4 [file 44318_2024_145_MOESM6_ESM.zip › Source_data_Figure_4/4E/Dyn1xA-R846A/3_Dyn1xA-R846A-GFP-Atto647_CONFOCAL.tif]

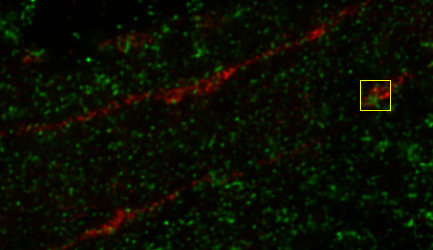

Supplement: Supplementary file 6 — Source data Fig. 4 [file 44318_2024_145_MOESM6_ESM.zip › Source_data_Figure_4/4E/Dyn1xA-R846A/3_Merged.tif]

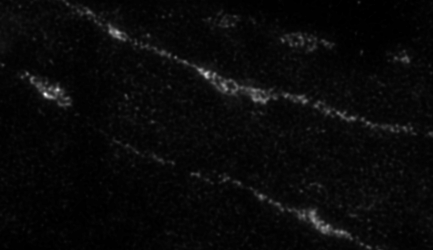

Supplement: Supplementary file 6 — Source data Fig. 4 [file 44318_2024_145_MOESM6_ESM.zip › Source_data_Figure_4/4E/Dyn1xA-R846A/3_Original_image_Decon_Dyn1xA-R846A-GFP-Atto647_aBassoon-AF594_STED.tif]

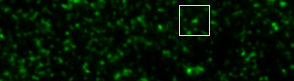

Supplement: Supplementary file 6 — Source data Fig. 4 [file 44318_2024_145_MOESM6_ESM.zip › Source_data_Figure_4/4E/Dyn1xA-S851D-857D/1_aBassoon-AF594_STED.tif]

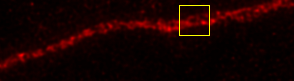

Supplement: Supplementary file 6 — Source data Fig. 4 [file 44318_2024_145_MOESM6_ESM.zip › Source_data_Figure_4/4E/Dyn1xA-S851D-857D/1_Dyn1xA-S851D-857D-GFP-Atto647.tif]

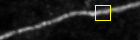

Supplement: Supplementary file 6 — Source data Fig. 4 [file 44318_2024_145_MOESM6_ESM.zip › Source_data_Figure_4/4E/Dyn1xA-S851D-857D/1_Dyn1xA-S851D-857D-GFP-Atto647_CONFOCAL.tif]

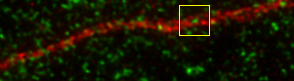

Supplement: Supplementary file 6 — Source data Fig. 4 [file 44318_2024_145_MOESM6_ESM.zip › Source_data_Figure_4/4E/Dyn1xA-S851D-857D/1_Merged.tif]

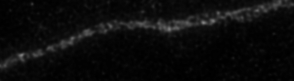

Supplement: Supplementary file 6 — Source data Fig. 4 [file 44318_2024_145_MOESM6_ESM.zip › Source_data_Figure_4/4E/Dyn1xA-S851D-857D/1_Original_image_Decon_Dyn1xA-S851D-857D-GFP-Atto647_aBassoon-AF594_STED.tif]

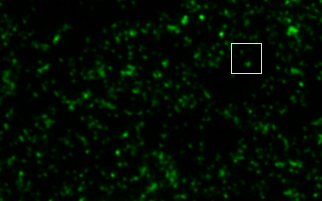

Supplement: Supplementary file 6 — Source data Fig. 4 [file 44318_2024_145_MOESM6_ESM.zip › Source_data_Figure_4/4E/Dyn1xA-S851D-857D/2_aBassoon-AF594_STED.tif]

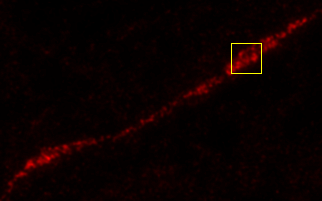

Supplement: Supplementary file 6 — Source data Fig. 4 [file 44318_2024_145_MOESM6_ESM.zip › Source_data_Figure_4/4E/Dyn1xA-S851D-857D/2_Dyn1xA-S851D-857D-GFP-Atto647.tif]

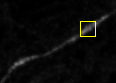

Supplement: Supplementary file 6 — Source data Fig. 4 [file 44318_2024_145_MOESM6_ESM.zip › Source_data_Figure_4/4E/Dyn1xA-S851D-857D/2_Dyn1xA-S851D-857D-GFP-Atto647_CONFOCAL.tif]

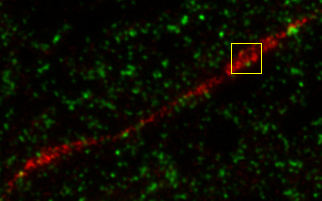

Supplement: Supplementary file 6 — Source data Fig. 4 [file 44318_2024_145_MOESM6_ESM.zip › Source_data_Figure_4/4E/Dyn1xA-S851D-857D/2_Merged.tif]

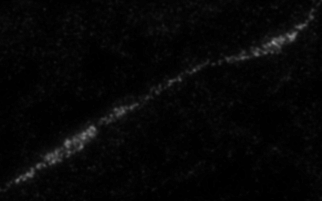

Supplement: Supplementary file 6 — Source data Fig. 4 [file 44318_2024_145_MOESM6_ESM.zip › Source_data_Figure_4/4E/Dyn1xA-S851D-857D/2_Original_image_Decon_Dyn1xA-S851D-857D-GFP-Atto647_aBassoon-AF594_STED.tif]

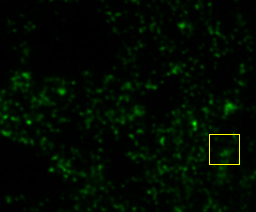

Supplement: Supplementary file 6 — Source data Fig. 4 [file 44318_2024_145_MOESM6_ESM.zip › Source_data_Figure_4/4E/Dyn1xA-S851D-857D/3_aBassoon-AF594_STED.tif]

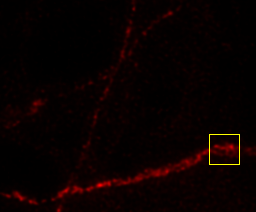

Supplement: Supplementary file 6 — Source data Fig. 4 [file 44318_2024_145_MOESM6_ESM.zip › Source_data_Figure_4/4E/Dyn1xA-S851D-857D/3_Dyn1xA-S851D-857D-GFP-Atto647.tif]

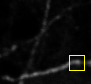

Supplement: Supplementary file 6 — Source data Fig. 4 [file 44318_2024_145_MOESM6_ESM.zip › Source_data_Figure_4/4E/Dyn1xA-S851D-857D/3_Dyn1xA-S851D-857D-GFP-Atto647_CONFOCAL.tif]

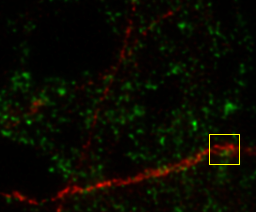

Supplement: Supplementary file 6 — Source data Fig. 4 [file 44318_2024_145_MOESM6_ESM.zip › Source_data_Figure_4/4E/Dyn1xA-S851D-857D/3_Merged.tif]

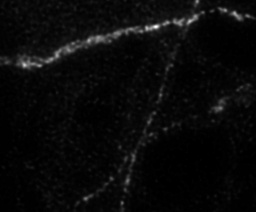

Supplement: Supplementary file 6 — Source data Fig. 4 [file 44318_2024_145_MOESM6_ESM.zip › Source_data_Figure_4/4E/Dyn1xA-S851D-857D/3_Original_image_Decon_Dyn1xA-S851D-857D-GFP-Atto647_aBassoon-AF594_STED.tif]

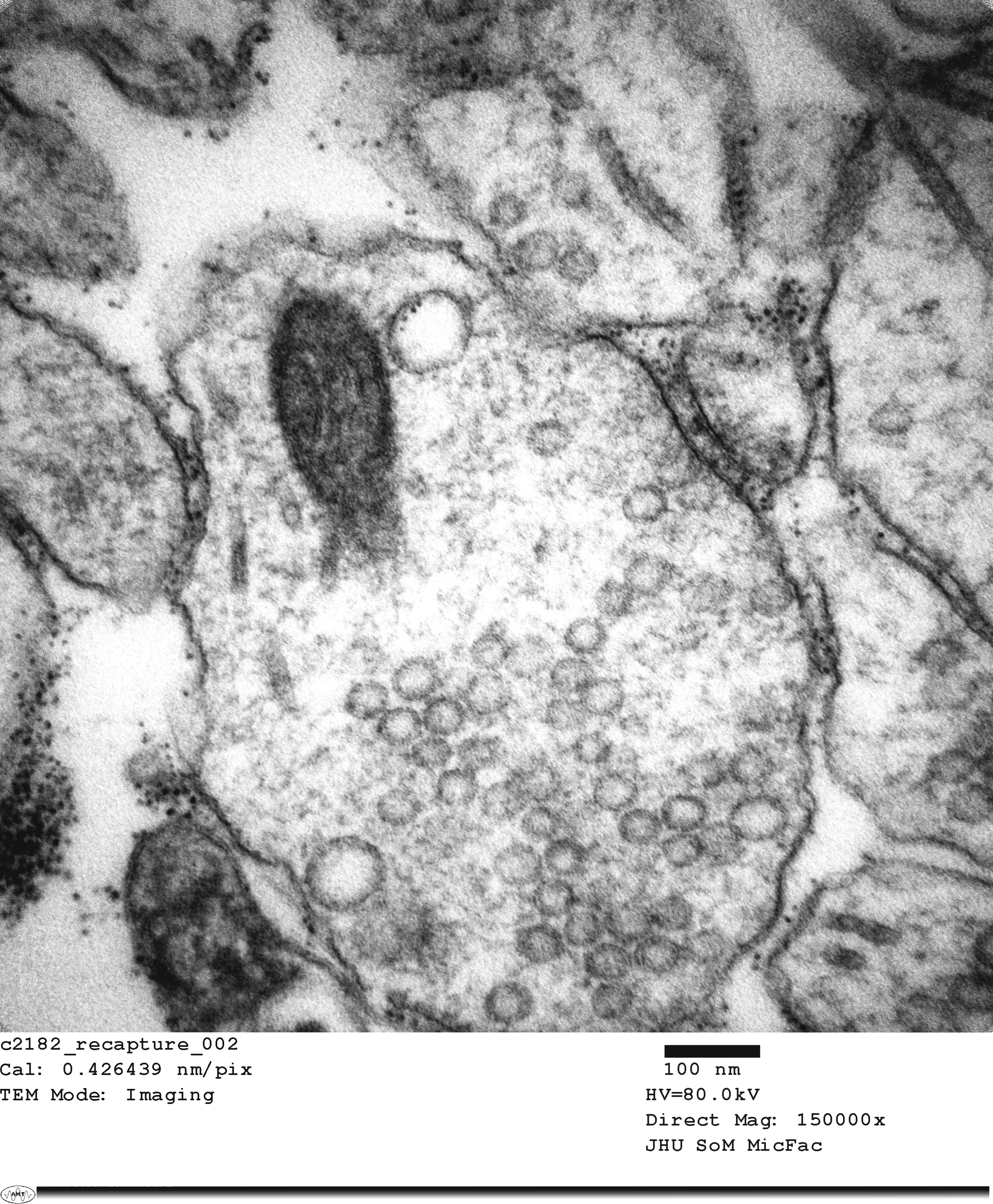

Supplement: Supplementary file 7 — Source data Fig. 5 [file 44318_2024_145_MOESM7_ESM.zip › Source_data_Figure_5/5A/c2182_recapture_002_(wild type 1s).tif]

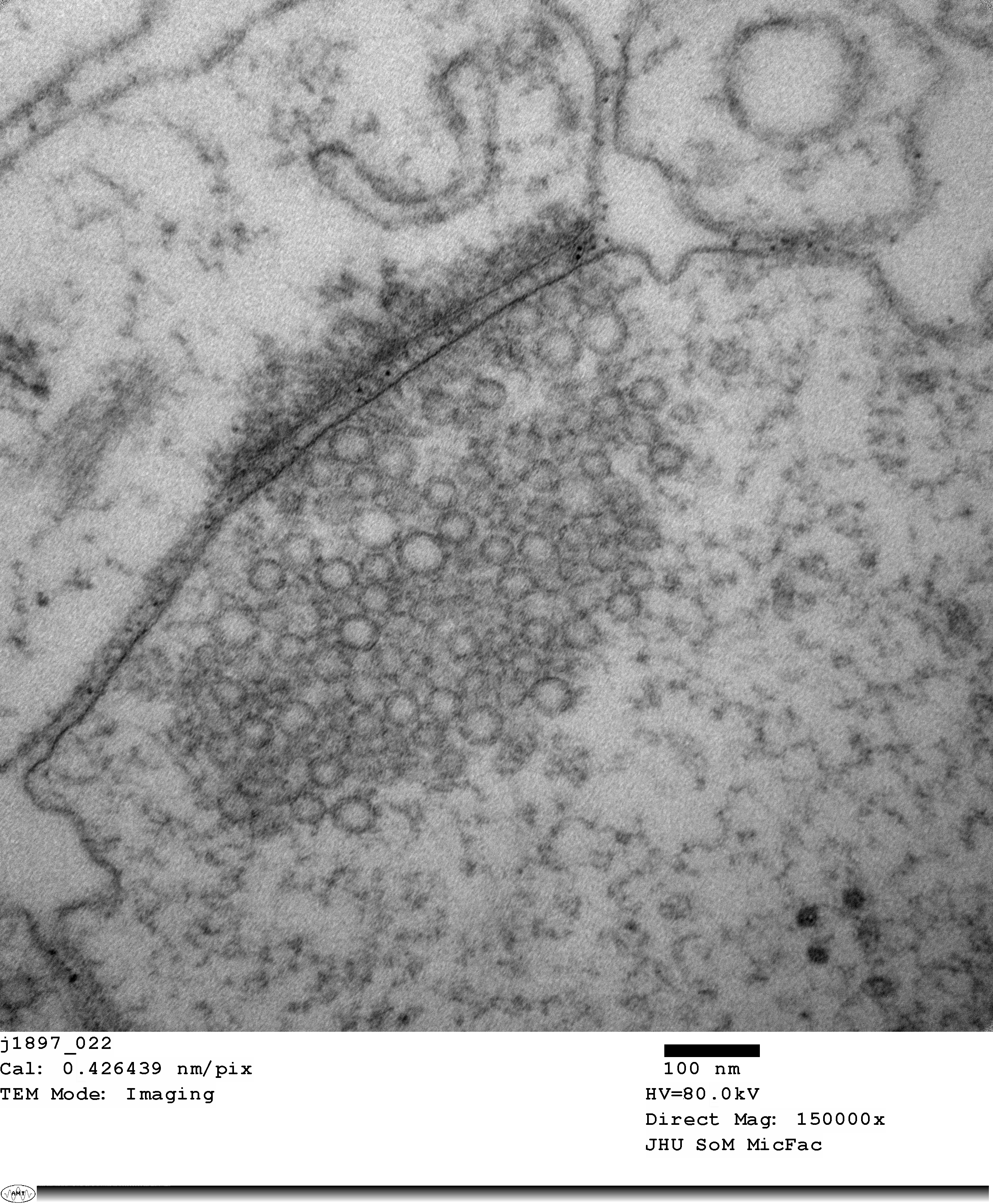

Supplement: Supplementary file 7 — Source data Fig. 5 [file 44318_2024_145_MOESM7_ESM.zip › Source_data_Figure_5/5A/j1897_022_16_(wild type 100ms).tif]

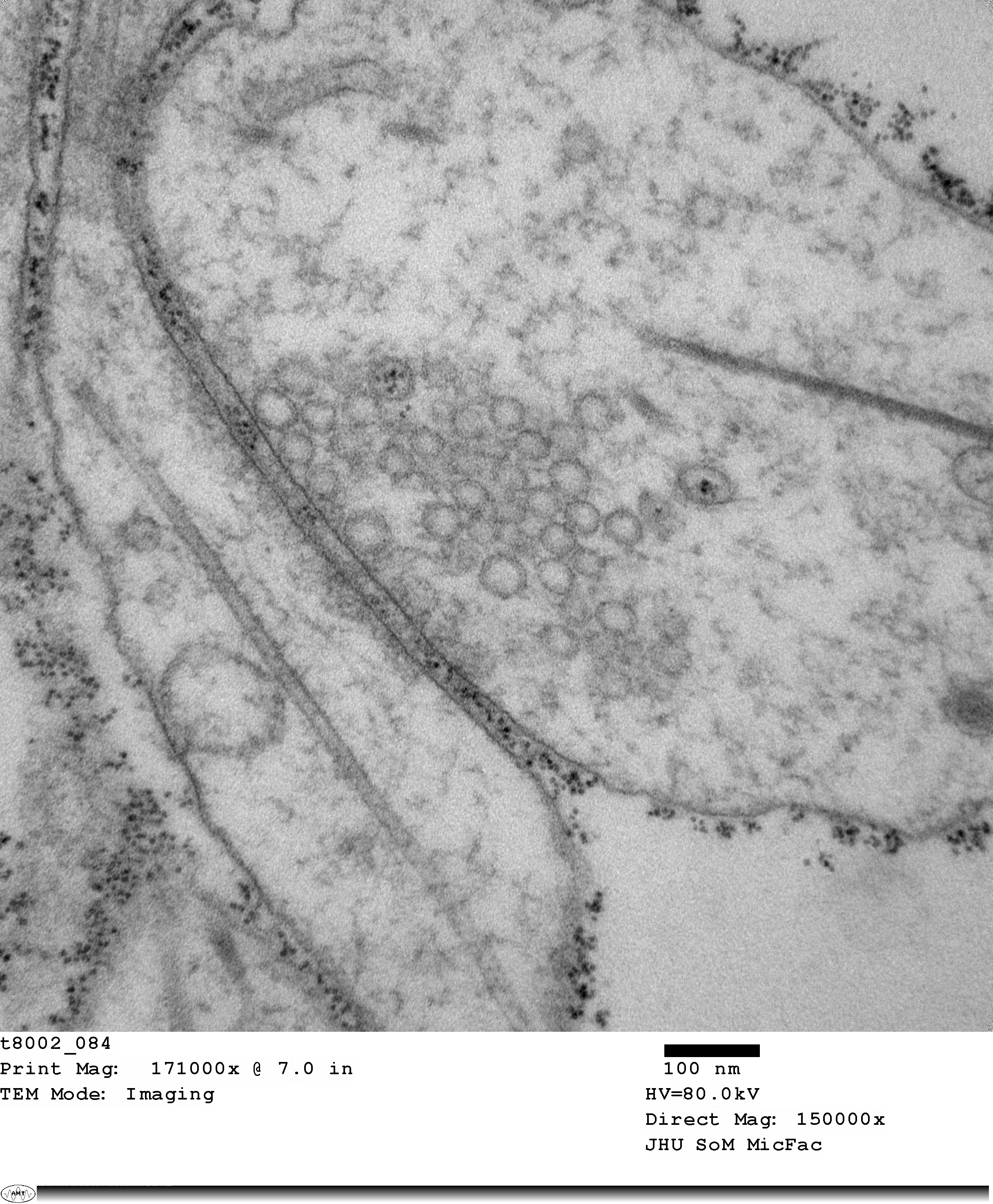

Supplement: Supplementary file 7 — Source data Fig. 5 [file 44318_2024_145_MOESM7_ESM.zip › Source_data_Figure_5/5A/t8002_084_16_(wild type 10s).tif]

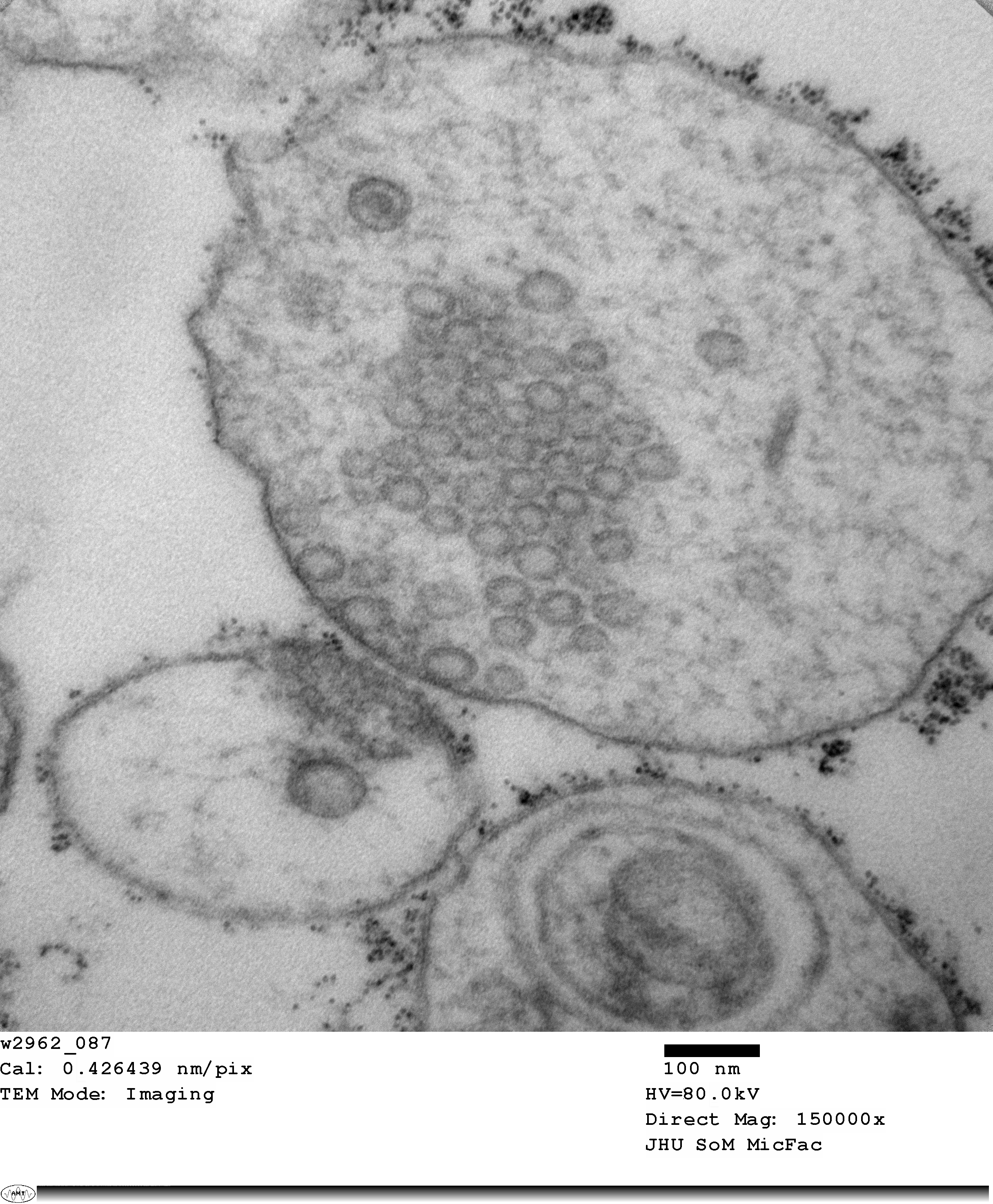

Supplement: Supplementary file 7 — Source data Fig. 5 [file 44318_2024_145_MOESM7_ESM.zip › Source_data_Figure_5/5A/w2962_087_16_(wild type no stim).tif]

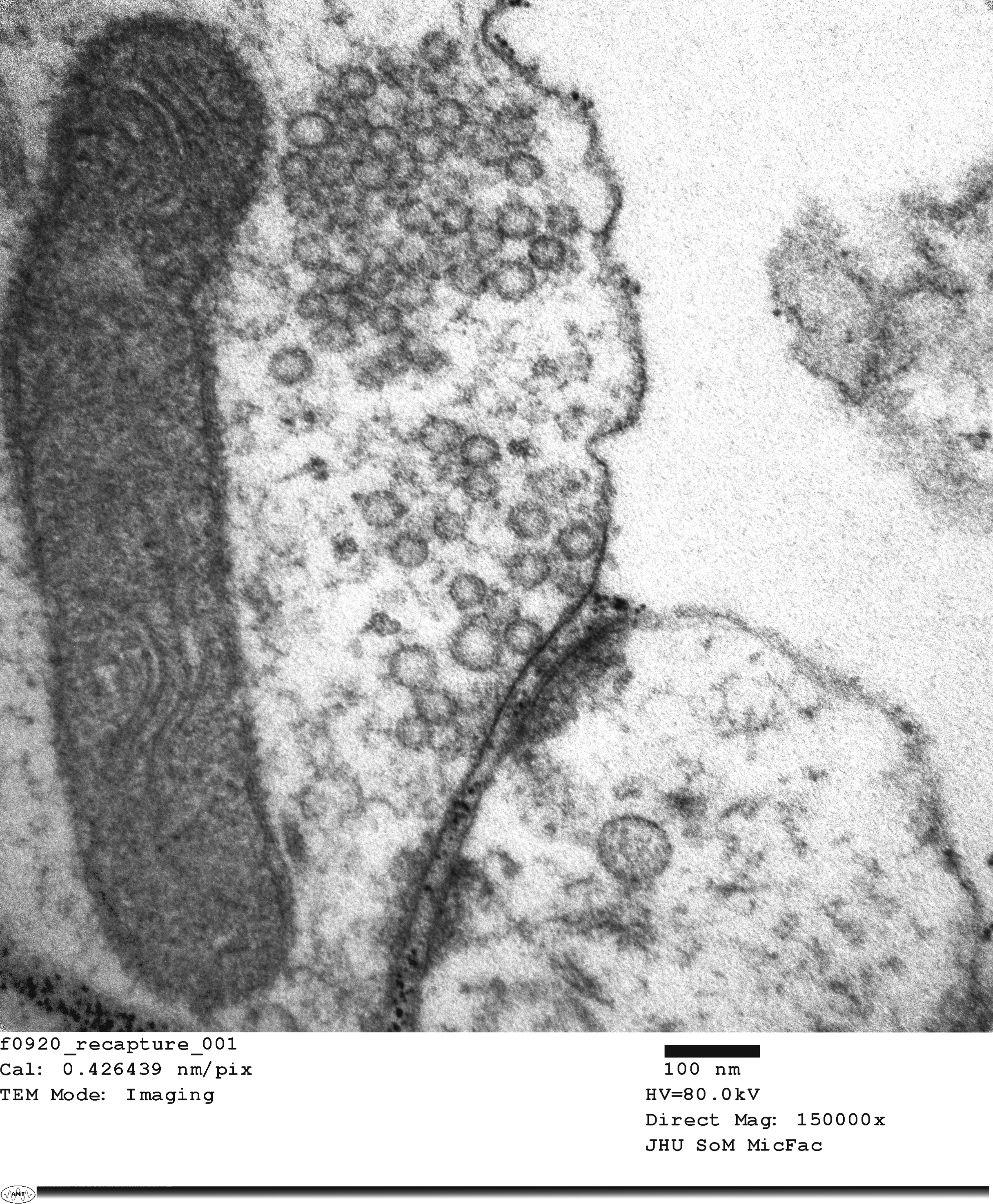

Supplement: Supplementary file 7 — Source data Fig. 5 [file 44318_2024_145_MOESM7_ESM.zip › Source_data_Figure_5/5B/f0920_recapture_001_(Dyn1KO 1s).tif]

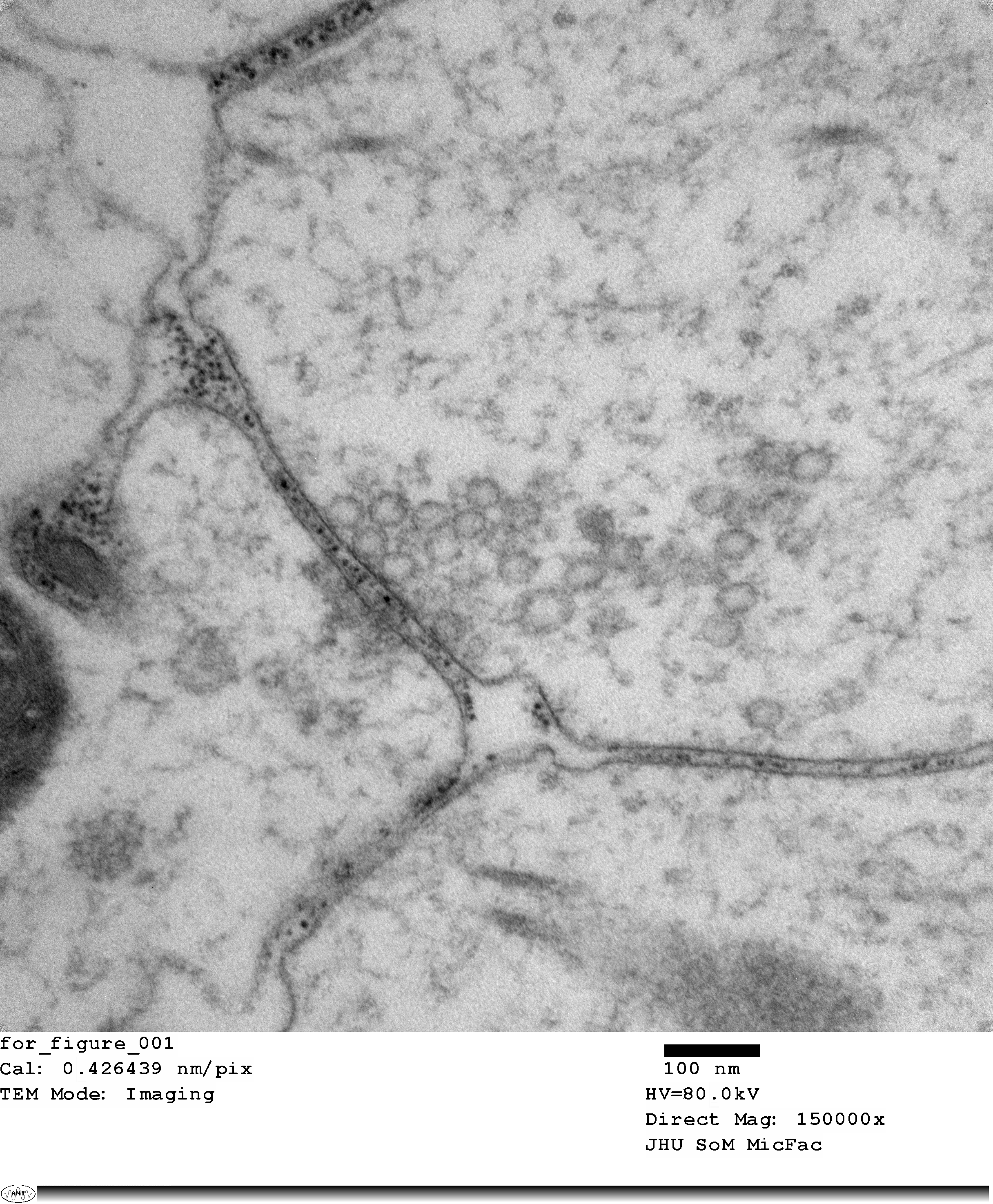

Supplement: Supplementary file 7 — Source data Fig. 5 [file 44318_2024_145_MOESM7_ESM.zip › Source_data_Figure_5/5B/g3139_recaptured_(Dyn1KO 100ms).TIF]

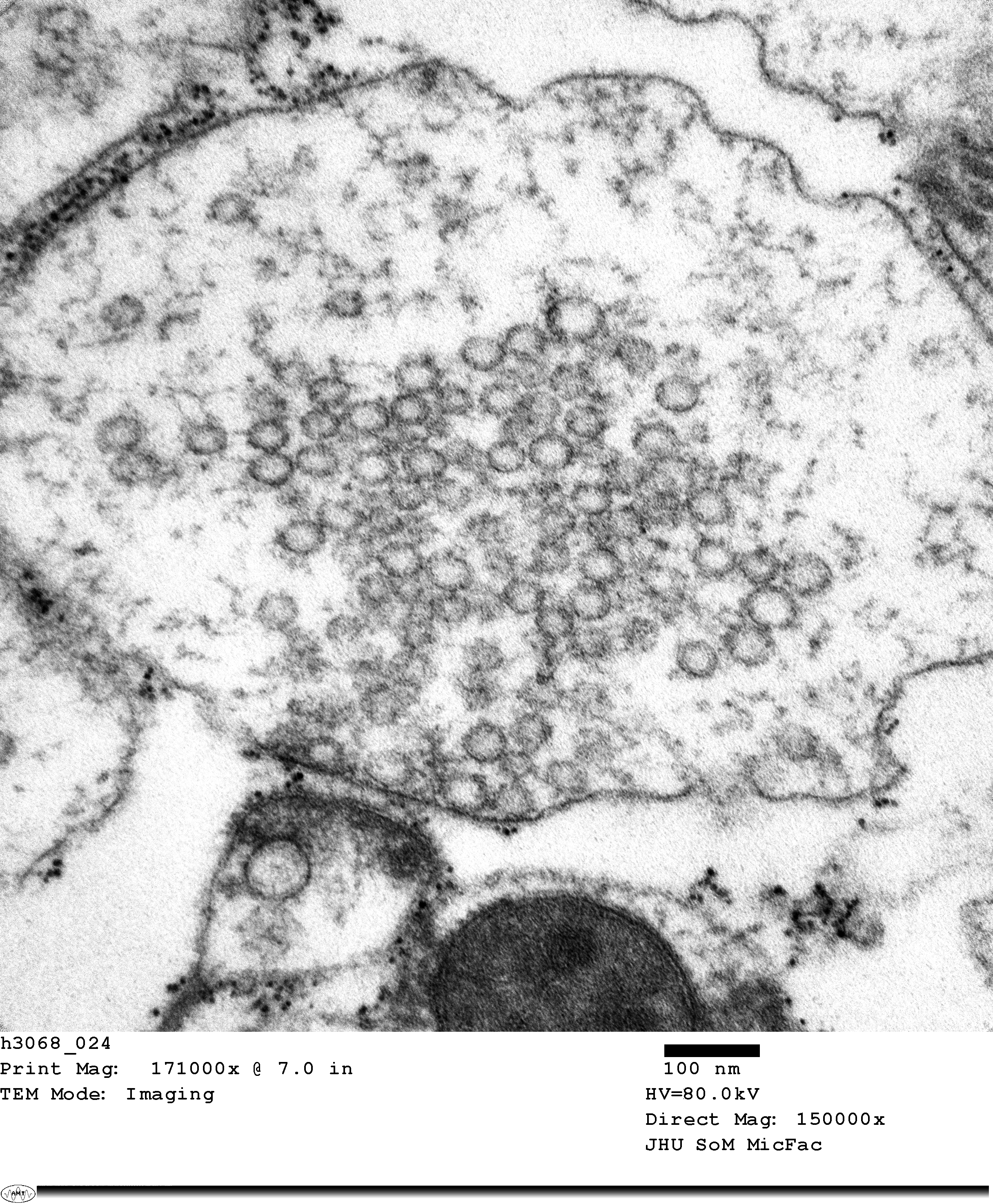

Supplement: Supplementary file 7 — Source data Fig. 5 [file 44318_2024_145_MOESM7_ESM.zip › Source_data_Figure_5/5B/h3068_024_16_(Dyn1KO no stim).tif]

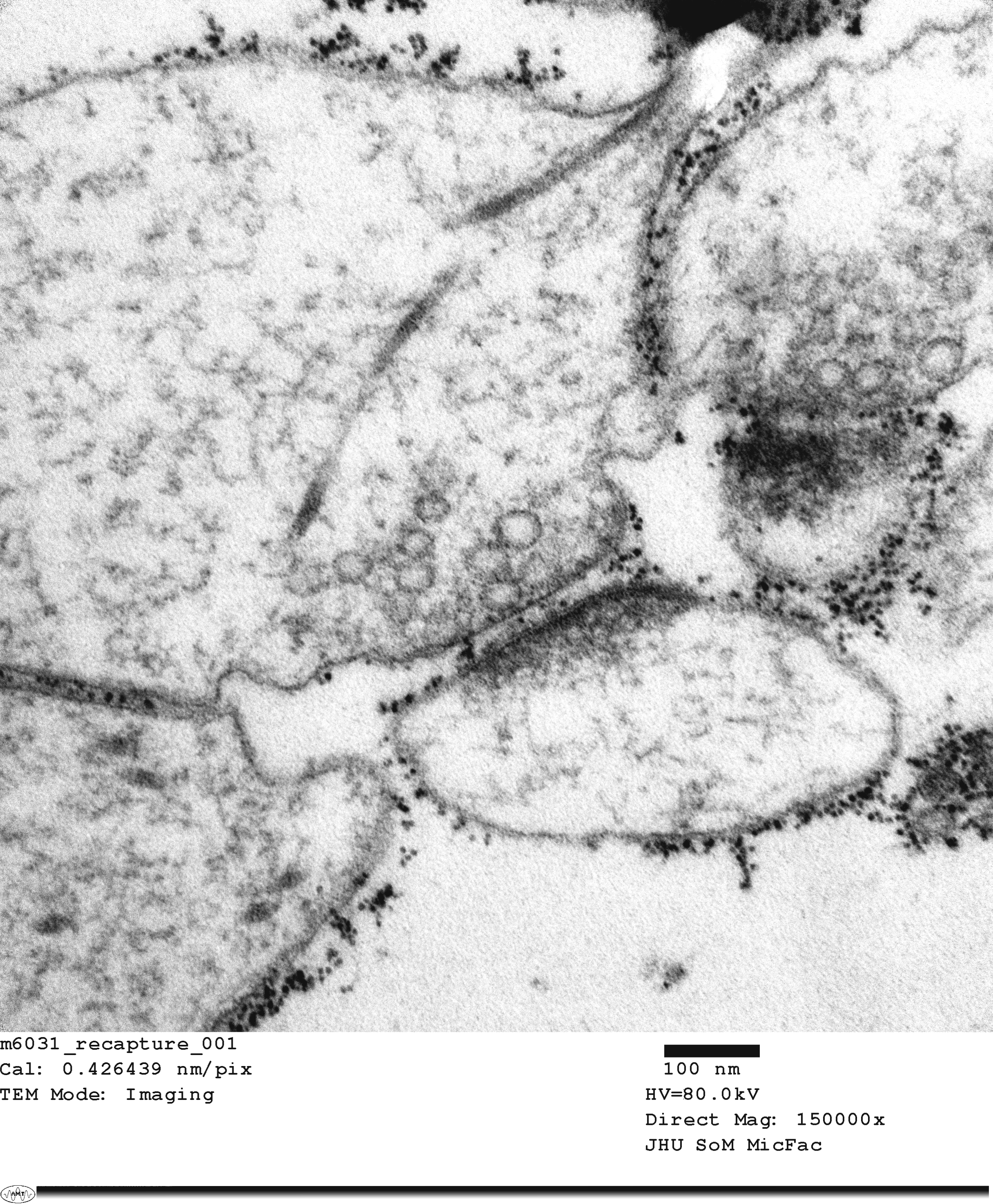

Supplement: Supplementary file 7 — Source data Fig. 5 [file 44318_2024_145_MOESM7_ESM.zip › Source_data_Figure_5/5B/m6031_recapture_001_(Dyn1KO 10s).tif]

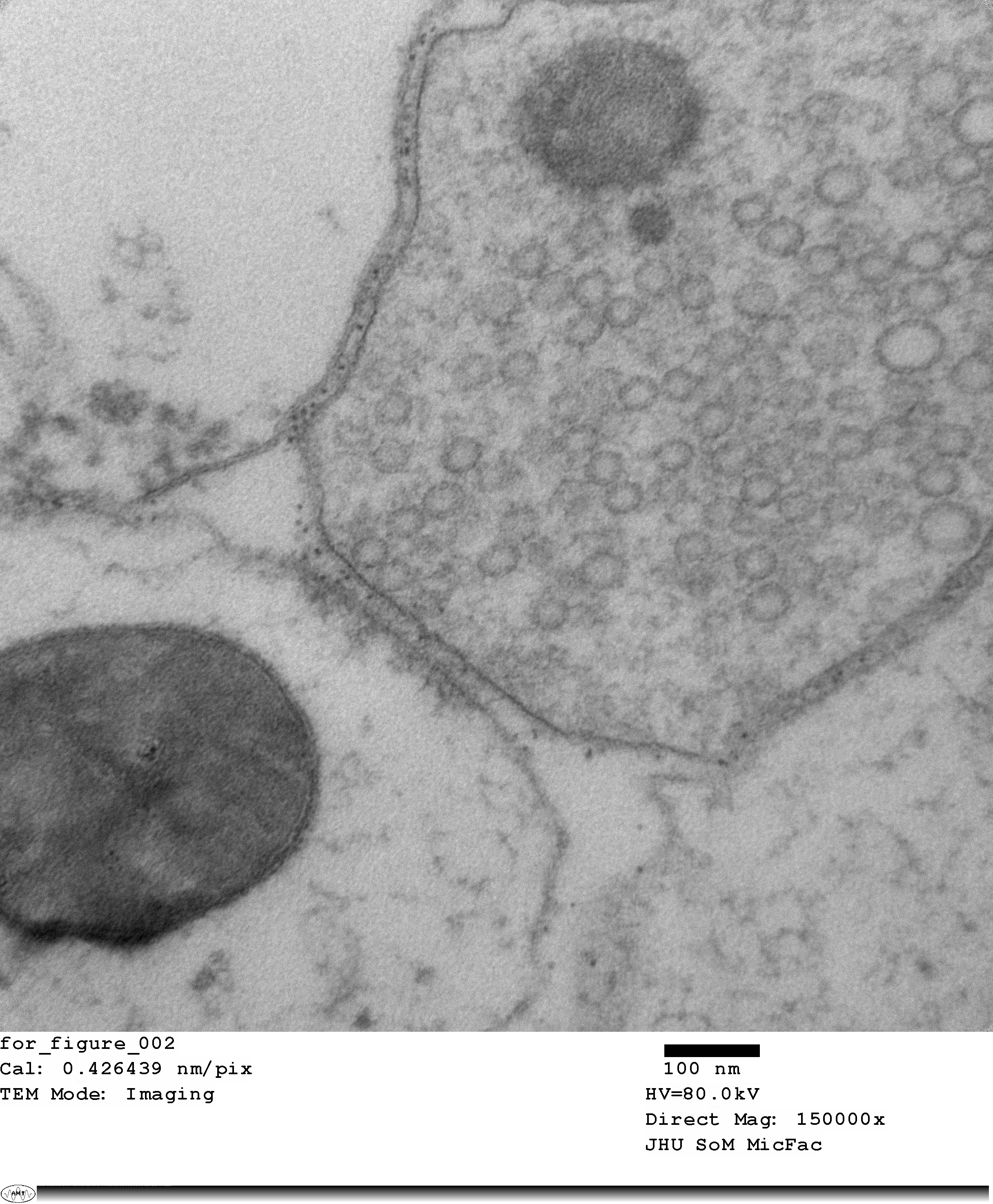

Supplement: Supplementary file 7 — Source data Fig. 5 [file 44318_2024_145_MOESM7_ESM.zip › Source_data_Figure_5/5C/a8902_recaptured_16_(Dyn1KO +Dyn1xA OE no stim).TIF]

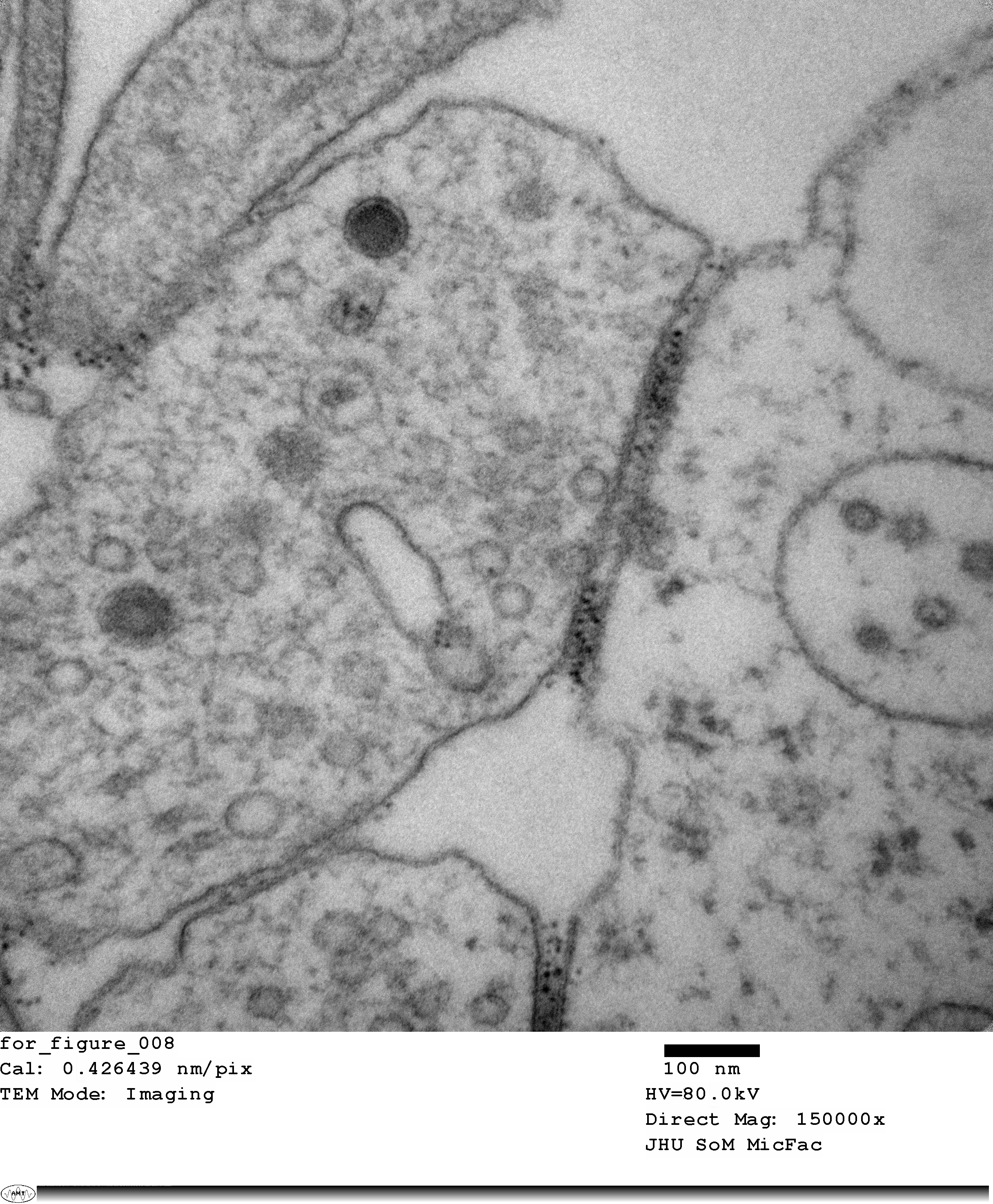

Supplement: Supplementary file 7 — Source data Fig. 5 [file 44318_2024_145_MOESM7_ESM.zip › Source_data_Figure_5/5C/d5087_recaptured_16_(Dyn1KO +dyn1xA OE 1s).tif]

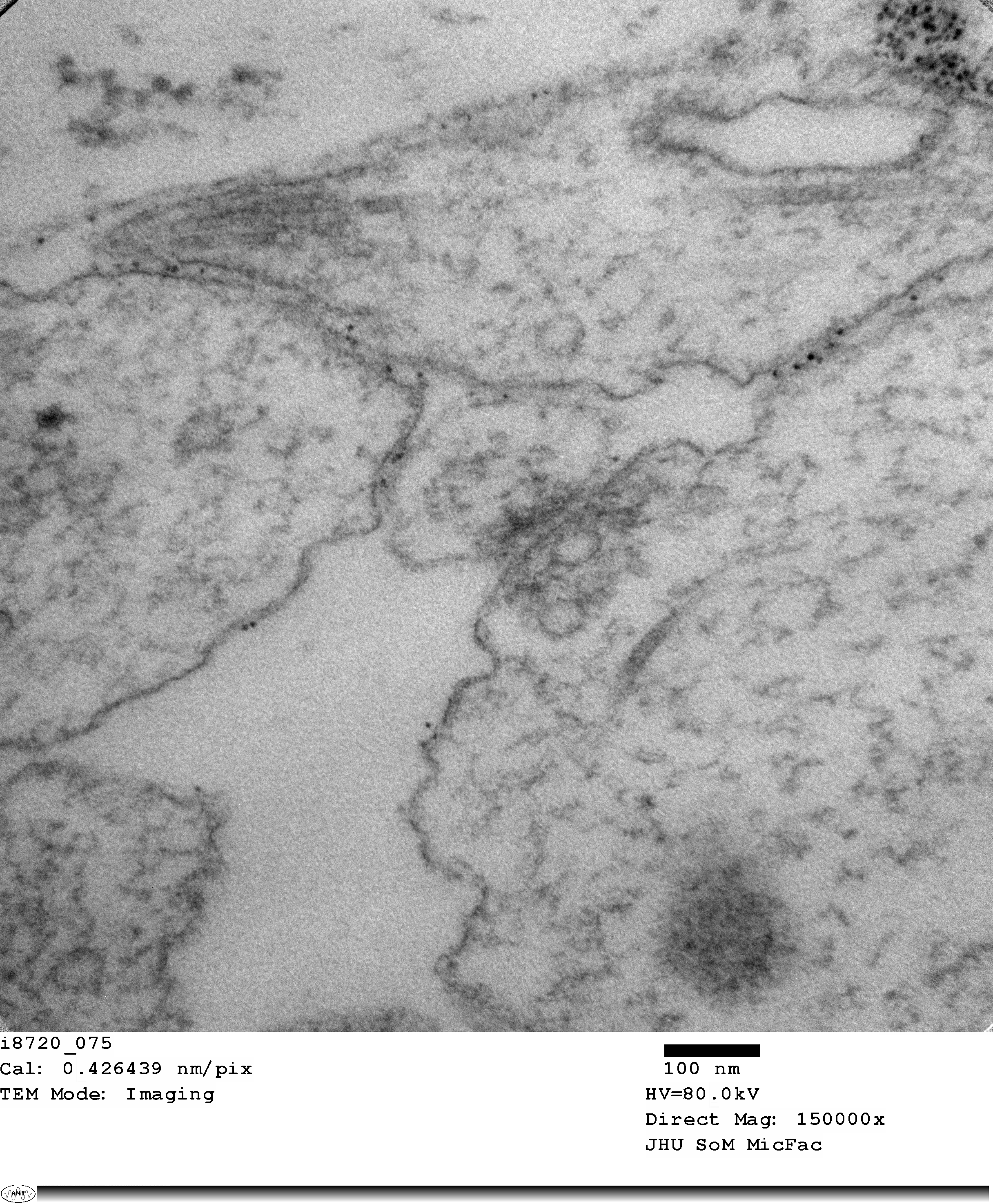

Supplement: Supplementary file 7 — Source data Fig. 5 [file 44318_2024_145_MOESM7_ESM.zip › Source_data_Figure_5/5C/i8720_075_16_(Dyn1KO +dyn1xA OE 100ms).tif]

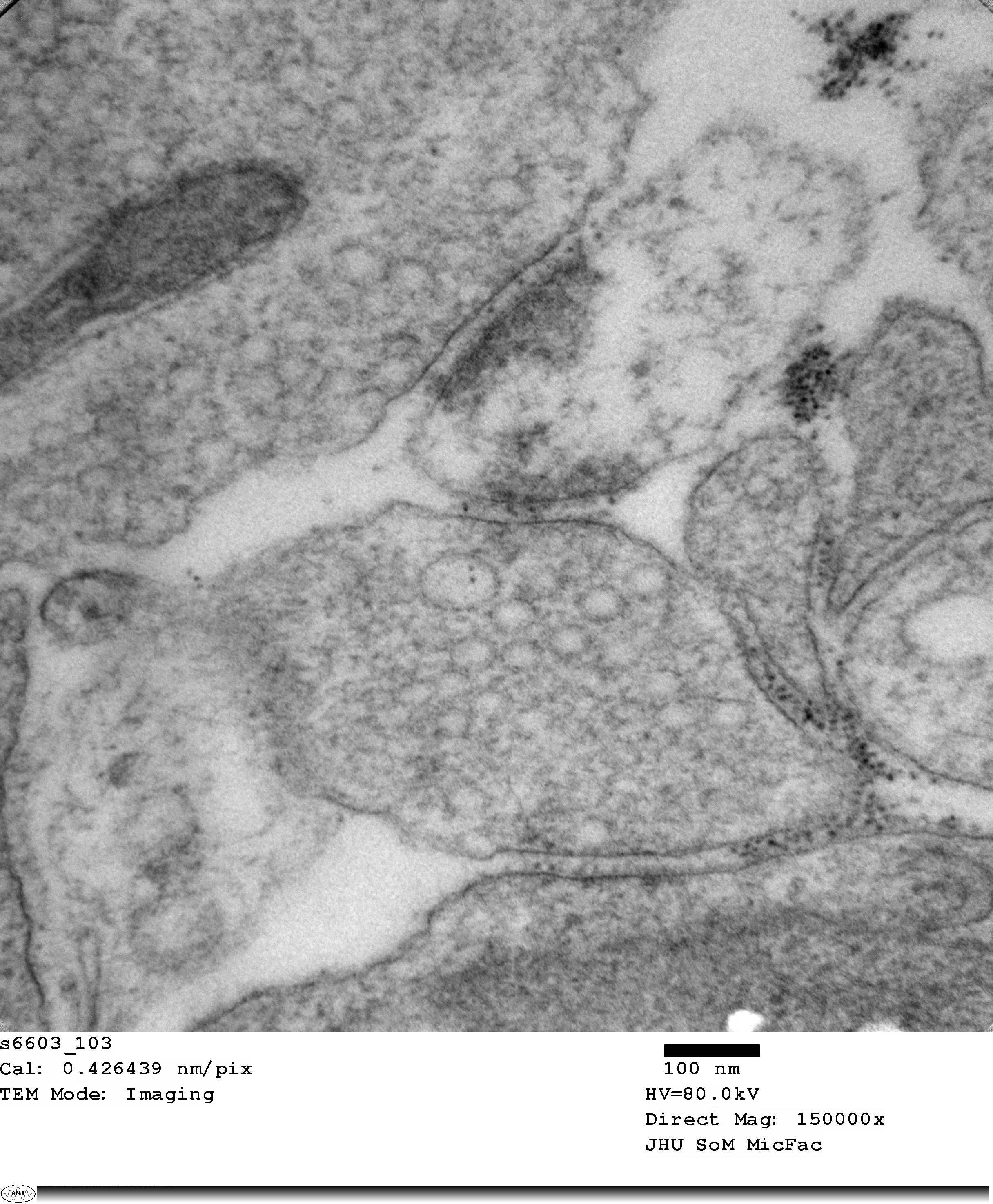

Supplement: Supplementary file 7 — Source data Fig. 5 [file 44318_2024_145_MOESM7_ESM.zip › Source_data_Figure_5/5C/s6603_103_16_(Dyn1KO +dyn1xA OE 1s).tif]

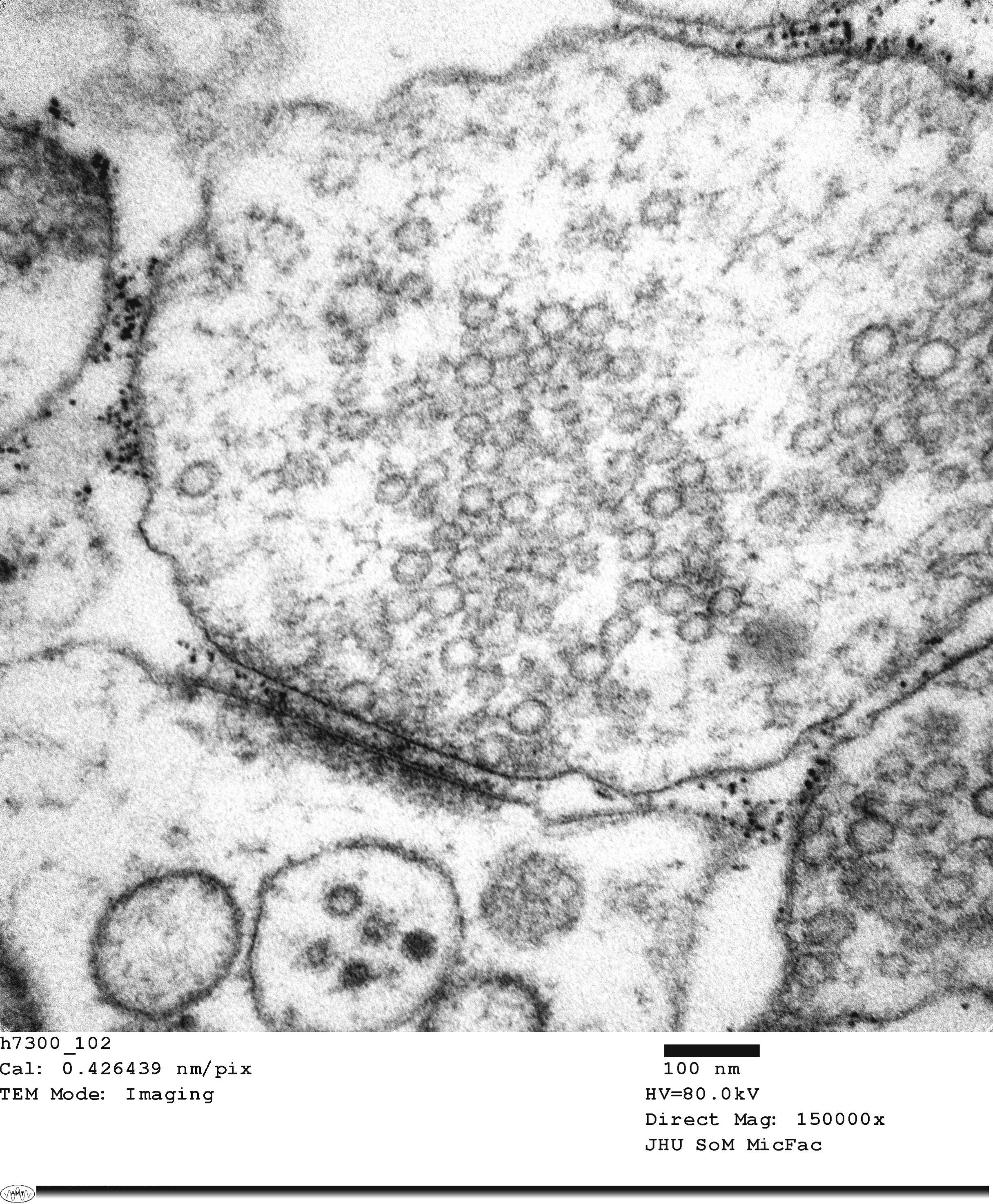

Supplement: Supplementary file 7 — Source data Fig. 5 [file 44318_2024_145_MOESM7_ESM.zip › Source_data_Figure_5/5D/h7300_102_(Dyn1KO +Dyn1xA-S851D-857D 100ms).tif]

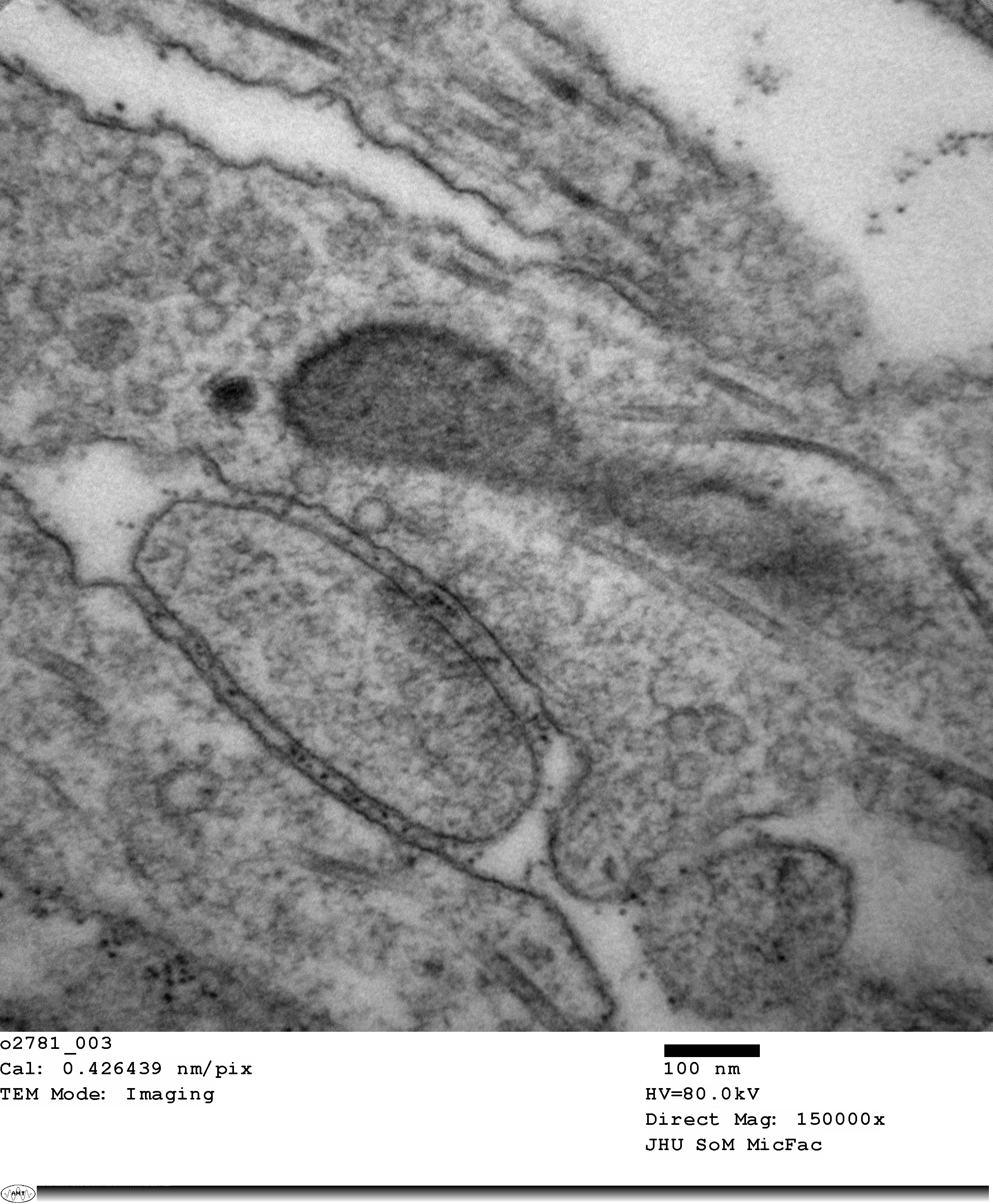

Supplement: Supplementary file 7 — Source data Fig. 5 [file 44318_2024_145_MOESM7_ESM.zip › Source_data_Figure_5/5D/o2781_003_16_(Dyn1KO +Dyn1xA-S851D-857D 1s).tif]

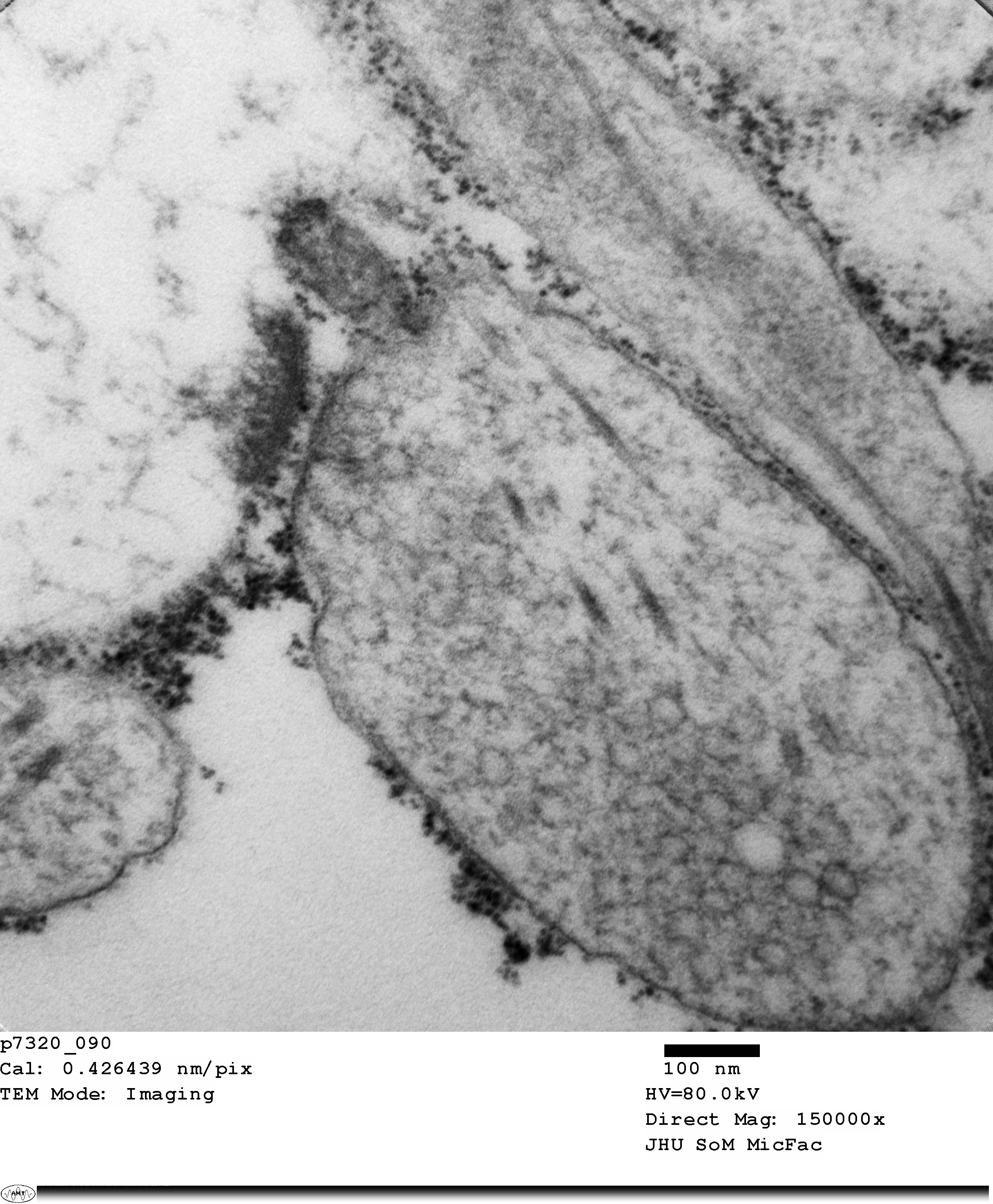

Supplement: Supplementary file 7 — Source data Fig. 5 [file 44318_2024_145_MOESM7_ESM.zip › Source_data_Figure_5/5D/p7320_090_16_(Dyn1KO +Dyn1xA-S851D-857D 10s).tif]

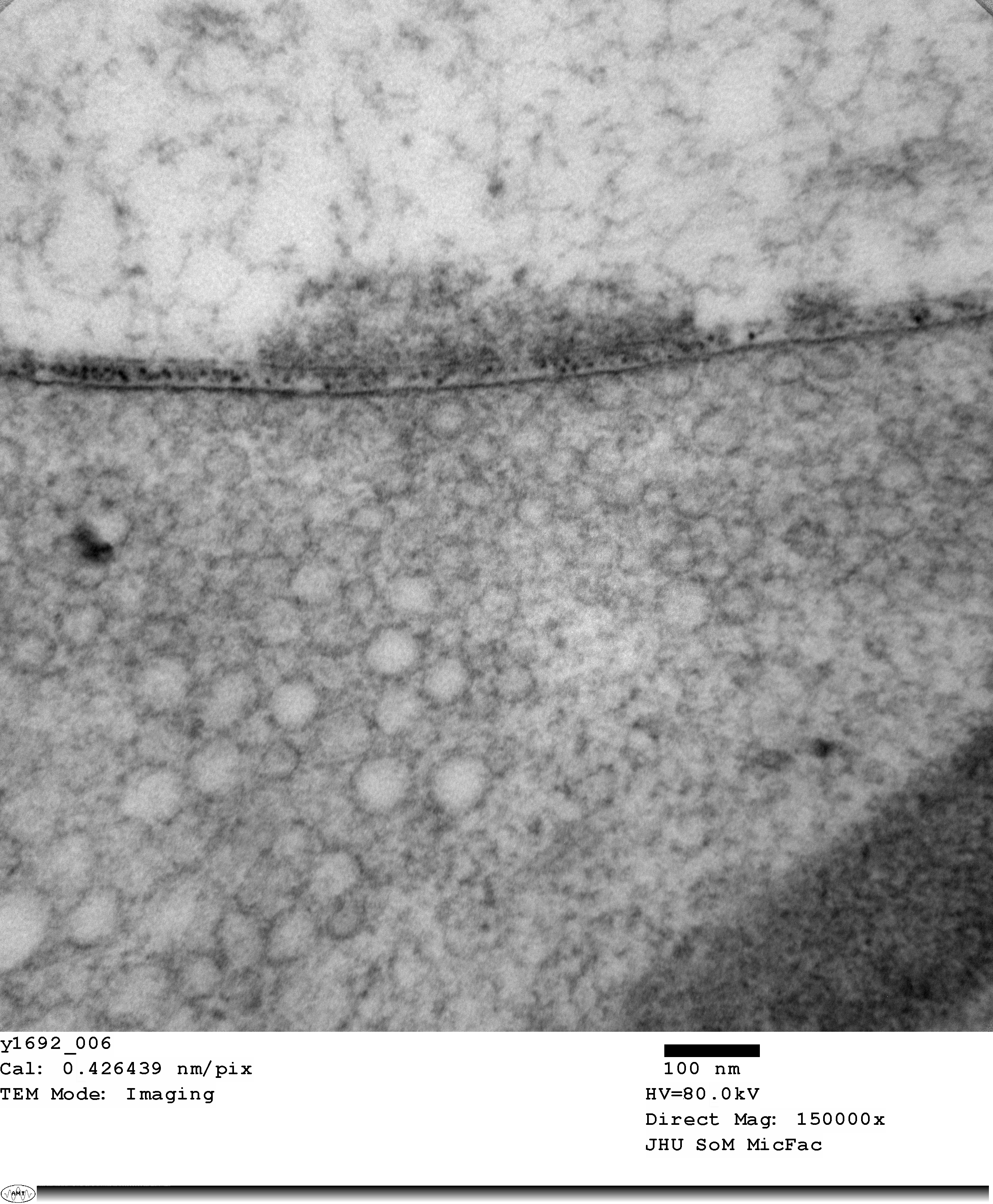

Supplement: Supplementary file 7 — Source data Fig. 5 [file 44318_2024_145_MOESM7_ESM.zip › Source_data_Figure_5/5D/y1692_006_16_(Dyn1KO +Dyn1xA-S851D-857D no stim).tif]

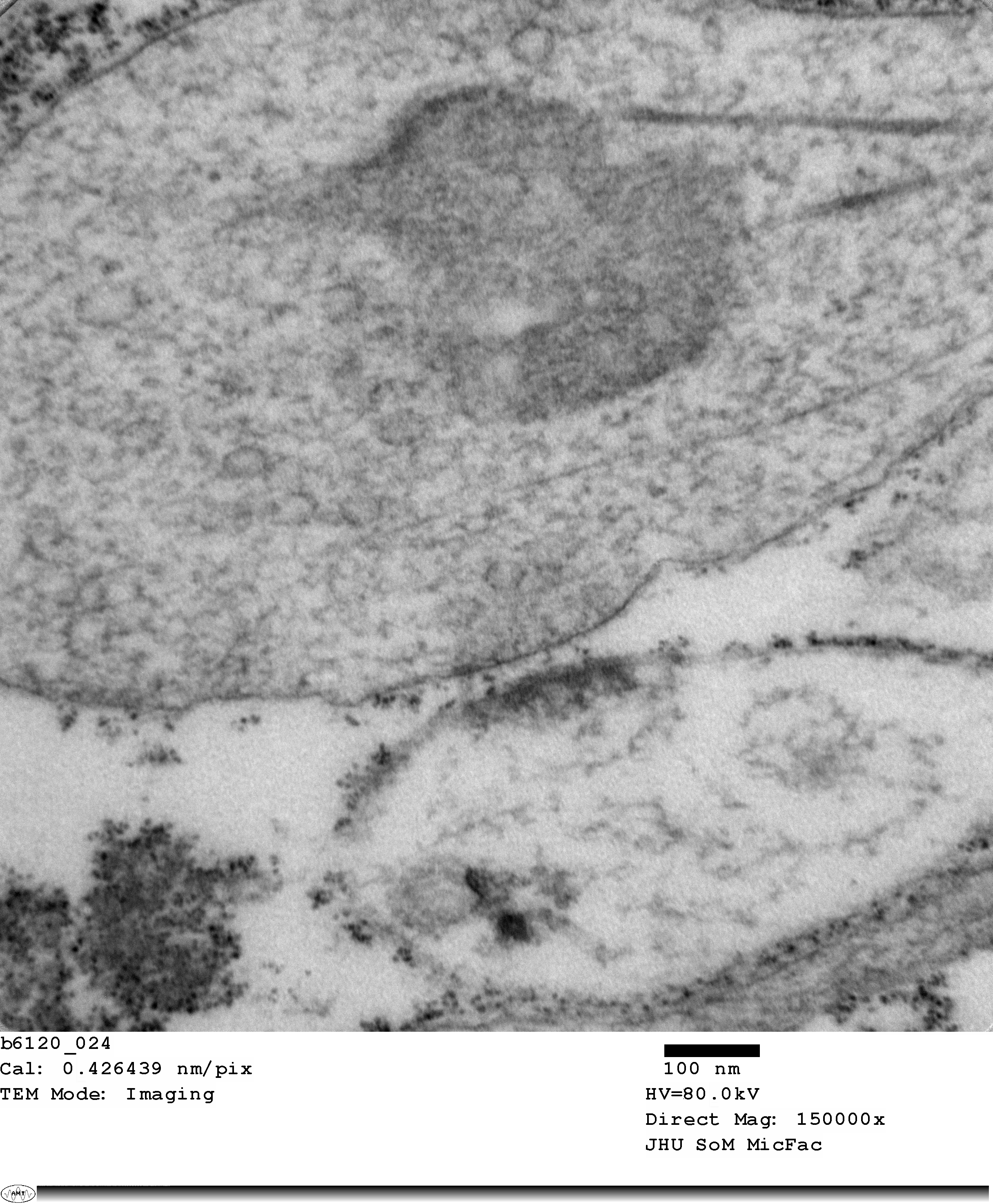

Supplement: Supplementary file 7 — Source data Fig. 5 [file 44318_2024_145_MOESM7_ESM.zip › Source_data_Figure_5/5E/b6120_024_16_(Dyn1KO +Dyn1xA-R846A 10s).tif]

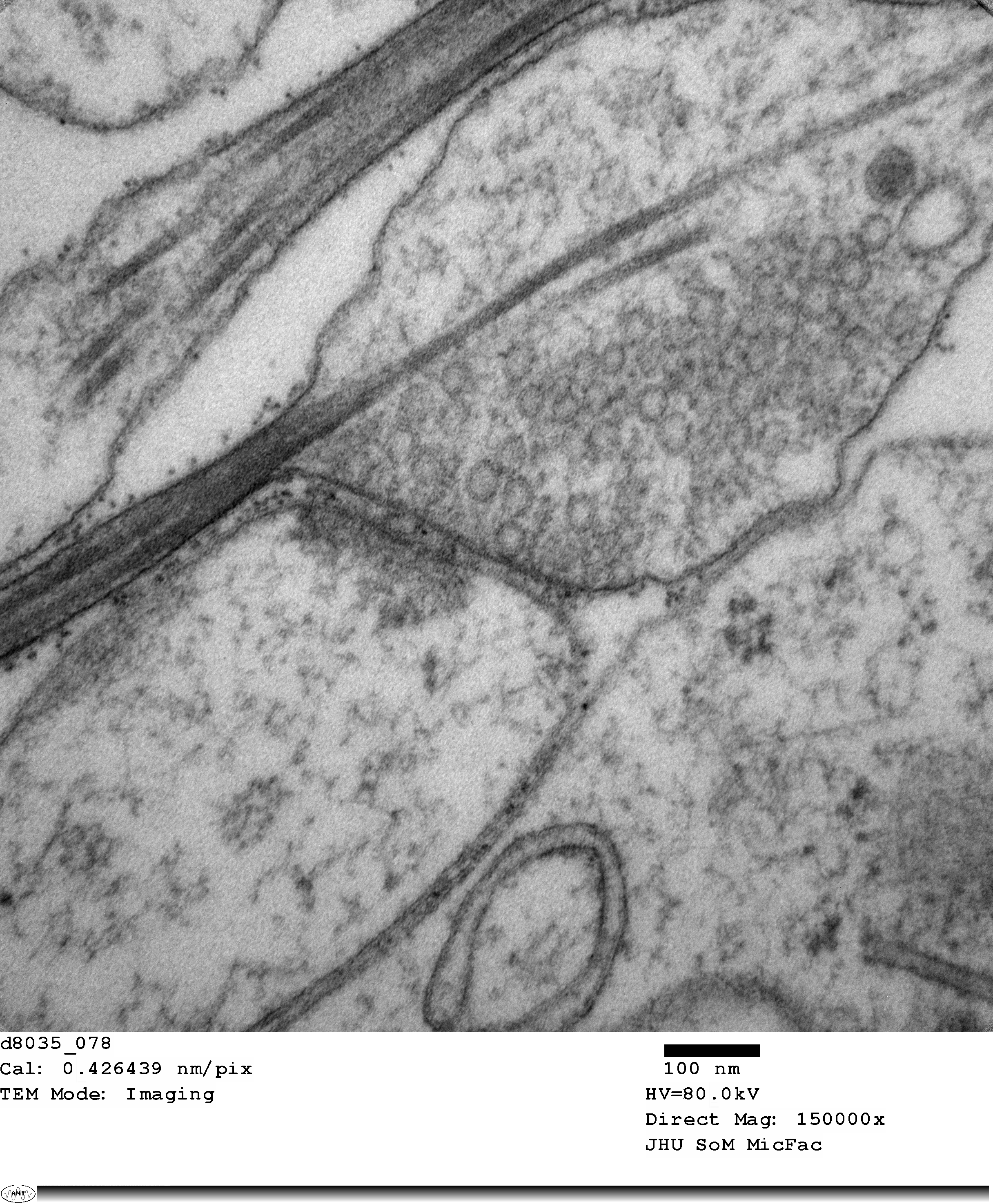

Supplement: Supplementary file 7 — Source data Fig. 5 [file 44318_2024_145_MOESM7_ESM.zip › Source_data_Figure_5/5E/d8035_078_16_(Dyn1KO +Dyn1xA-R846A 100ms).tif]

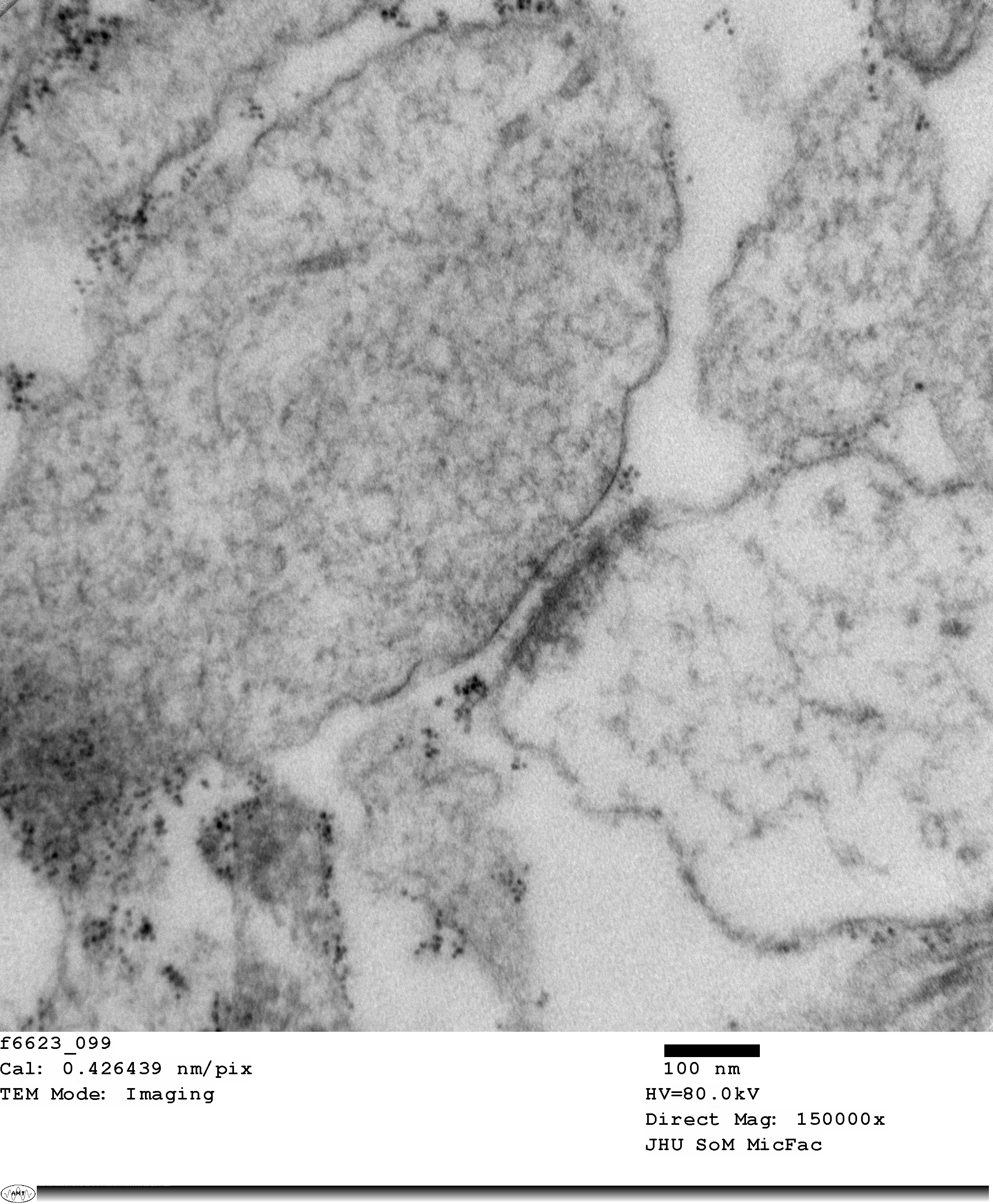

Supplement: Supplementary file 7 — Source data Fig. 5 [file 44318_2024_145_MOESM7_ESM.zip › Source_data_Figure_5/5E/f6623_099_16_(Dyn1KO +Dyn1xA-R846A 1s).tif]

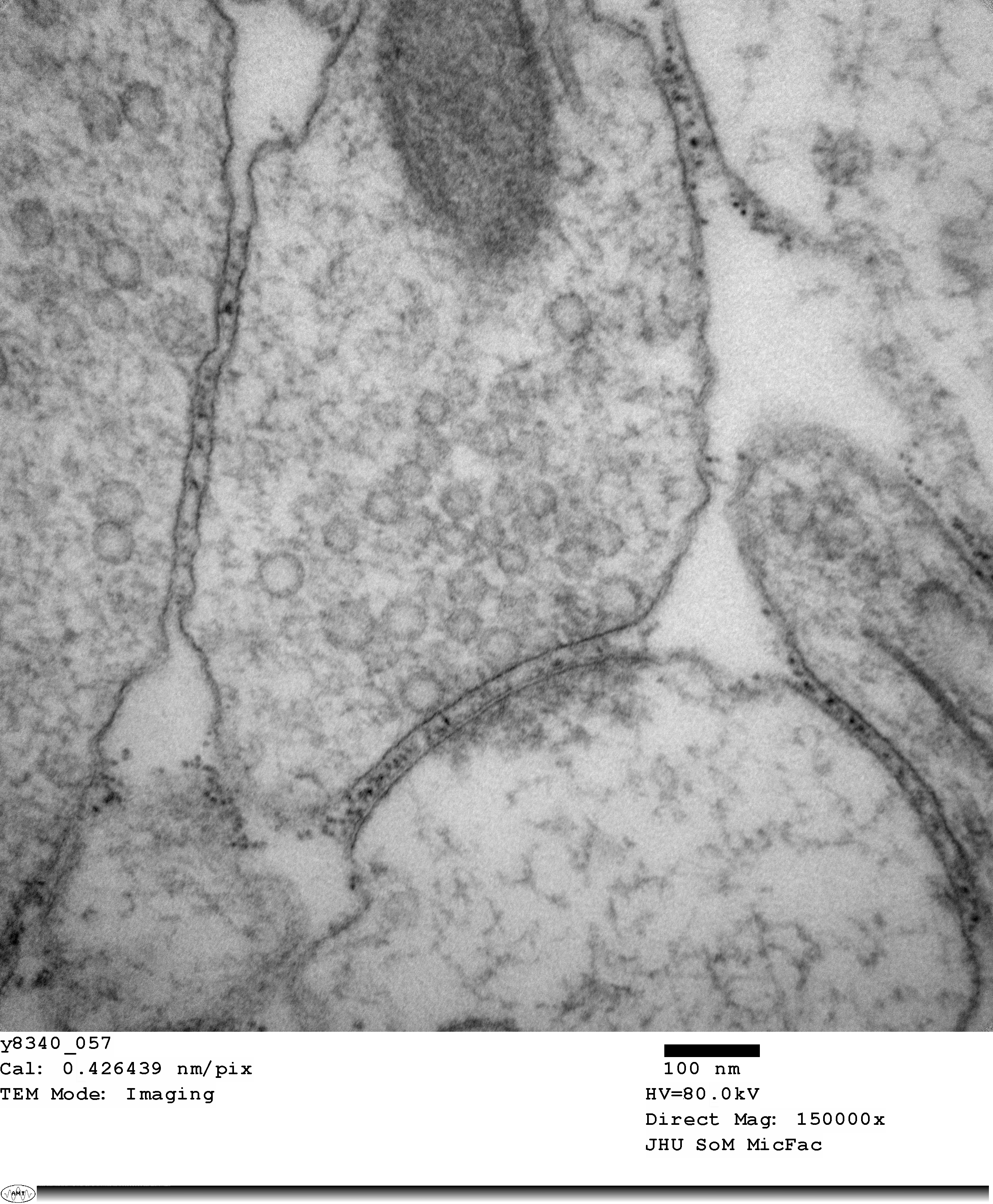

Supplement: Supplementary file 7 — Source data Fig. 5 [file 44318_2024_145_MOESM7_ESM.zip › Source_data_Figure_5/5E/y8340_057_16_(Dyn1KO +Dyn1xA-R846A no stim).tif]
