# Supplementary material for: Dynamin 1xA interacts with Endophilin A1 via its spliced long C-terminus for ultrafast endocytosis
Source: EMBO J. 2024 Jun 21;43(16):3327–57. doi: 10.1038/s44318-024-00145-x (PMC11329700; doi:10.1038/s44318-024-00145-x)
Supplement: Supplementary file 9 — Expanded View Figures [file 44318_2024_145_MOESM9_ESM.pdf]

Expanded View Figures

A

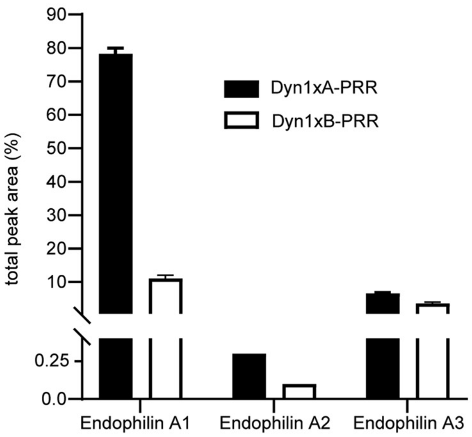

B

Endophilin A1

1. K.VGGAEGTKLDDDFKEMER.K [21, 38]
2. R.AVMEIMTK.T [46, 53]
3. K.LSMINTMSK.I [68, 76]
4. K.QNFIDPLQNLHDK.D [137, 149]
5. K.QAVQILQQVTVR.L [228, 239]
6. R.ALYDFEPENEGELGFK.E [297, 312]

Endophilin A2

1. K.VGGAEGTKLDDDFREMEK.K [21, 38
2. K.QNFIDPLQNLCDK.D [137, 149]
3. R.QAVQILEELADK.L [228, 239]
4. K.ITASSSFR.S [283, 290]
5. K.ALYDFEPENDGELGFR.E [313, 328]

Endophilin A3

1. K.ASQLFSEK.I [13, 20]
2. K.ATEYLQPNPAYR.A [54, 65]
3. K.DSLDINVK.Q [129, 136]
4. R.QSTEILQELQNK.L [228, 239]
5. R.IALASQVPR.R [244, 252]
6. R.GLYDFEPENEGELGFK.E [292, 307]

Figure EV1. Mass spectrometry evidence for identification of Endophilin A1 bound to Dyn1xA-PRR.

(**A**) Rat brain synaptosome lysates were incubated with GST-Dyn1-PRR (either xA or xB) coupled to GSH-sepharose beads. Bound proteins were separated by SDS-PAGE and stained with Coomassie blue. The protein band at 40 kDa from both GST-Dyn1-PRR (either xA or xB) were individually cut and digested using Trypsin/LysC and analyzed using a targeted LC–MS/MS SRM analyses. The total amount of each Endophilin protein bound to each Dyn1-PRR was calculated by summing the total peak area for each Q1/Q3 transition to provide the total peak area. The percentage of the total peak area for each protein was then calculated. Endophilin A1 was the predominant protein with a level of Endophilin A3 at 11-fold lower levels, while A2 levels were more than 250-fold lower than A1. (**B**) List of unique Endophilin isoform-specific peptides used for SRM assay (rat sequences: Endophilin-A1_sp|O35179|SH3G2, Endophilin-A2-sp|O35964|SH3G1, Endophilin-A3-sp|O35180|SH3G3).

 © The Author(s)

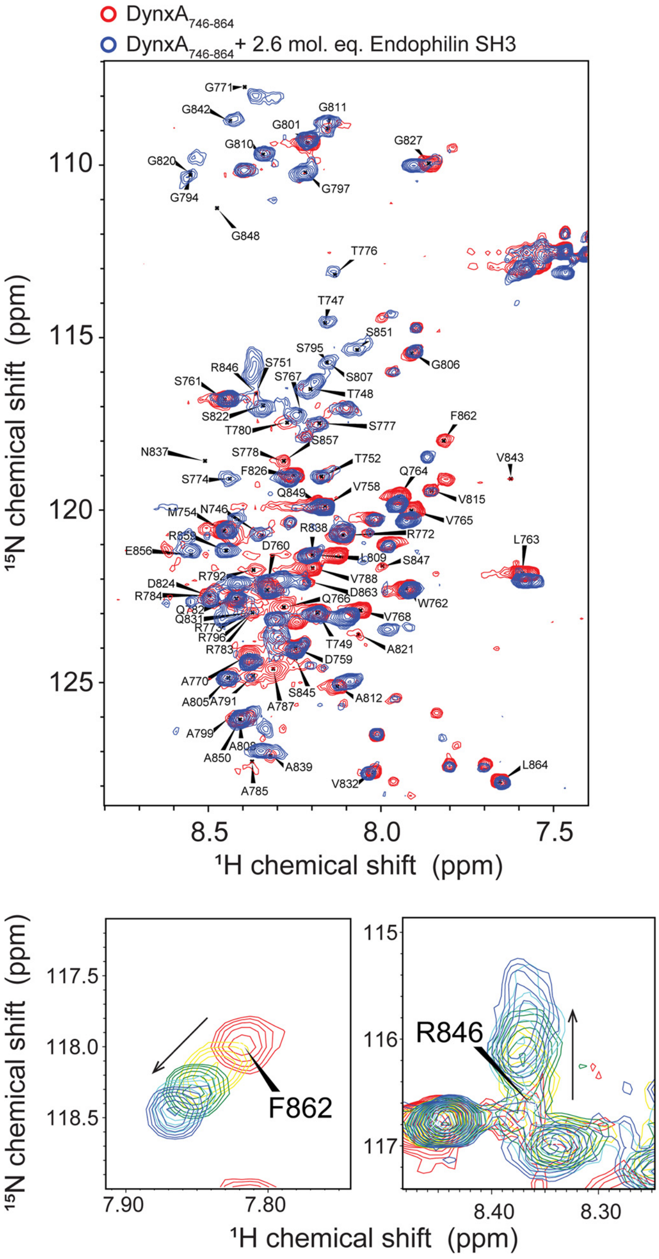

Figure EV2. ^{15}N-HSQC titration data for the dynamin–endophilin interaction.

Partial ^{15}N-HSQC spectra are shown for 0.27 mM ^{15}N-DynxA$_{746\text{-}864}$ alone (red) and for the same protein following the addition of 2.6 molar equivalents of unlabeled Endophilin A1 SH3 domain. Assignments are shown for DynxA alone. Note that some signals for which assignment locations are marked are not visible in this spectrum but were visible in other spectra recorded for longer times. The bottom panels show cropped portions of R846 and F862 from the top panel. Arrows indicate peak shifting during the titration processes.

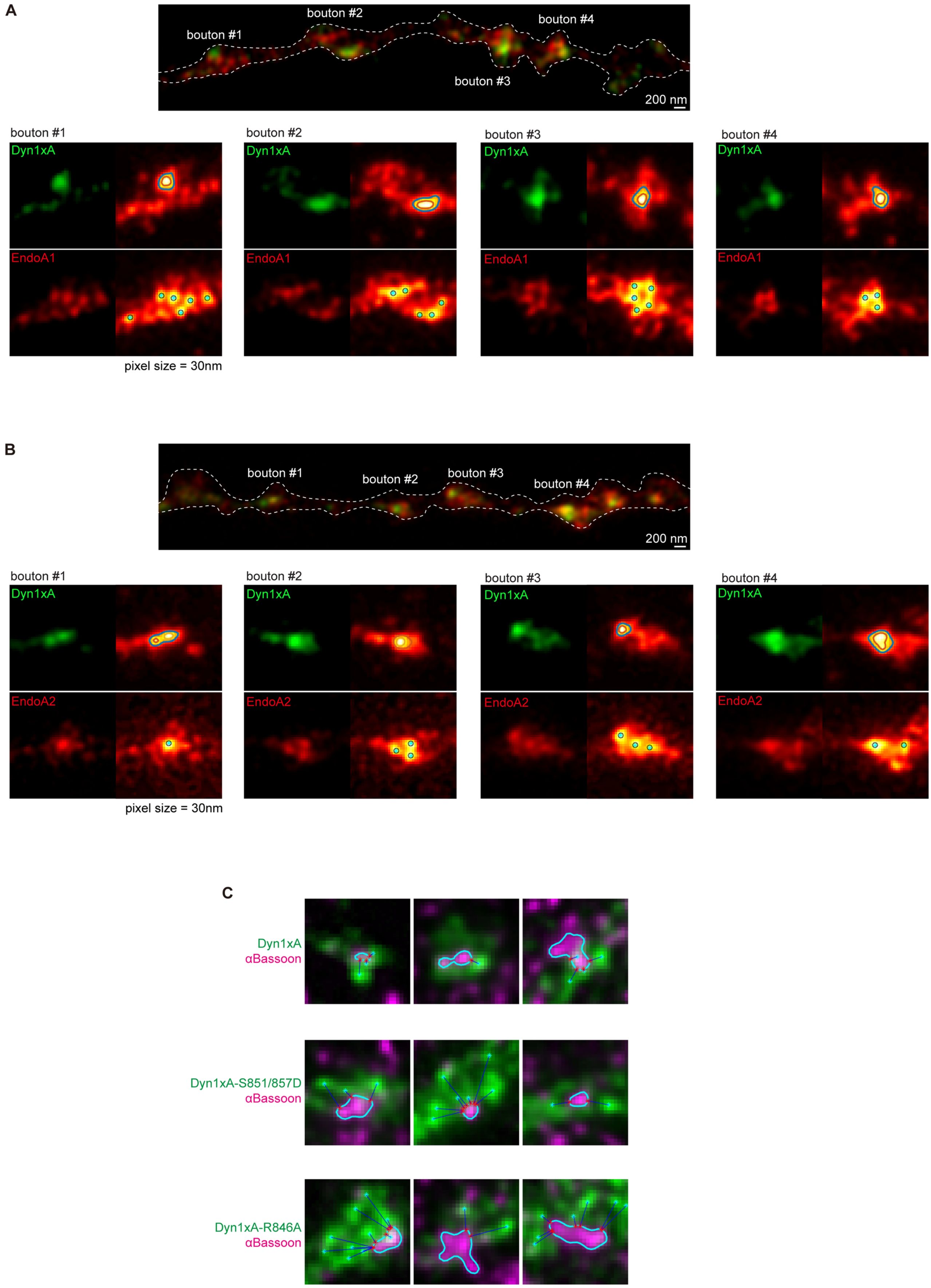
A
bouton #1
bouton #2
bouton #3
bouton #4
200 nm
Dyn1xA
EndoA1
pixel size = 30nm
B
bouton #1
bouton #2
bouton #3
bouton #4
200 nm
Dyn1xA
EndoA2
pixel size = 30nm
C
Dyn1xA
αBassoon
Dyn1xA-S851/857D
αBassoon
Dyn1xA-R846A
αBassoon

 © The Author(s)

Figure EV3. Additional STED images for Fig. 4.

(**A**) The top image shows an axon containing multiple boutons. Signals show overexpression of GFP-tagged Dyn1xA (Dyn1xA) and mCherry-tagged Endophilin A1 (EndoA1). The bottom images show magnifications of four boutons in the top image. Red hot LUT images on the right side of Dyn1xA and EndoA1 images are enhanced contrast images. Outer and inner contour 50% and 70% of local maxima of the Dyn1xA, respectively. Black circles represent local maxima of Endophilin A1. In these boutons, there are multiple EndophilinA1 puncta in each bouton. (**B**) The top image shows an axon containing multiple boutons. Signals show overexpression of mCherry-tagged Dyn1xA (Dyn1xA) and GFP-tagged Endophilin A2 (EndoA2). The bottom images show magnifications of four boutons in the top image. Red hot LUT images on the right side of Dyn1xA and EndoA2 images are enhanced contrast images. Outer and inner contour 50% and 70% of local maxima of the Dyn1xA, respectively. Black circles represent local maxima of Endophilin A2. In these boutons, there are multiple EndophilinA2 puncta in each bouton. (**C**) STED micrographs of the same synapses as in Fig. 4E with an active zone marker Bassoon (magenta) visualized by antibody staining of GFP-tagged Dyn1xA, Dyn1xA S851D/857D or Dyn1xA R846A (green). Local maxima of Dyn1xA, Dyn1xA S851D/857D or Dyn1xA R846A signals and minimum distance to the active zone boundary are overlayed.

© The Author(s)

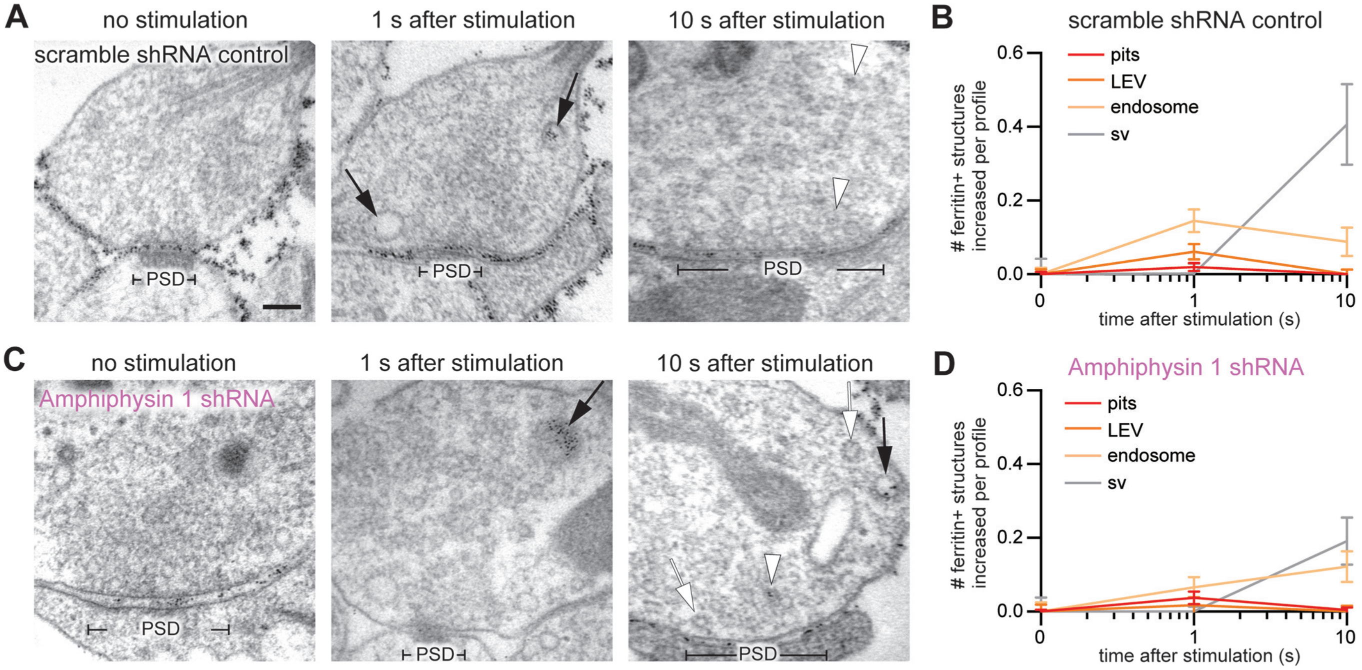

Figure EV4. Amphiphysin 1 is not essential for ultrafast endocytosis.

(**A**, **C**) Example micrographs showing endocytic pits and ferritin-containing endocytic structures at the indicated time points in neurons expressing scramble RNA (**A**) and Amphiphysin 1 shRNA (**C**). Black arrows, ferritin-positive large endocytic vesicles (LEVs) or endosomes; white arrowheads, ferritin-positive synaptic vesicles. Scale bar: 100 nm. PSD, post-synaptic density. (**B**, **D**) Plots showing the increase in the number of each endocytic structure per synaptic profile after a single stimulus in neurons expressing scramble RNA (**B**) and Amphiphysin 1 shRNA (**D**). The mean and SEM are shown in each graph. All data are from two independent experiments from $N = 2$ cultures prepared and frozen on different days. n = scramble RNA, 436; Amphiphysin 1 shRNA, 609.

 © The Author(s)

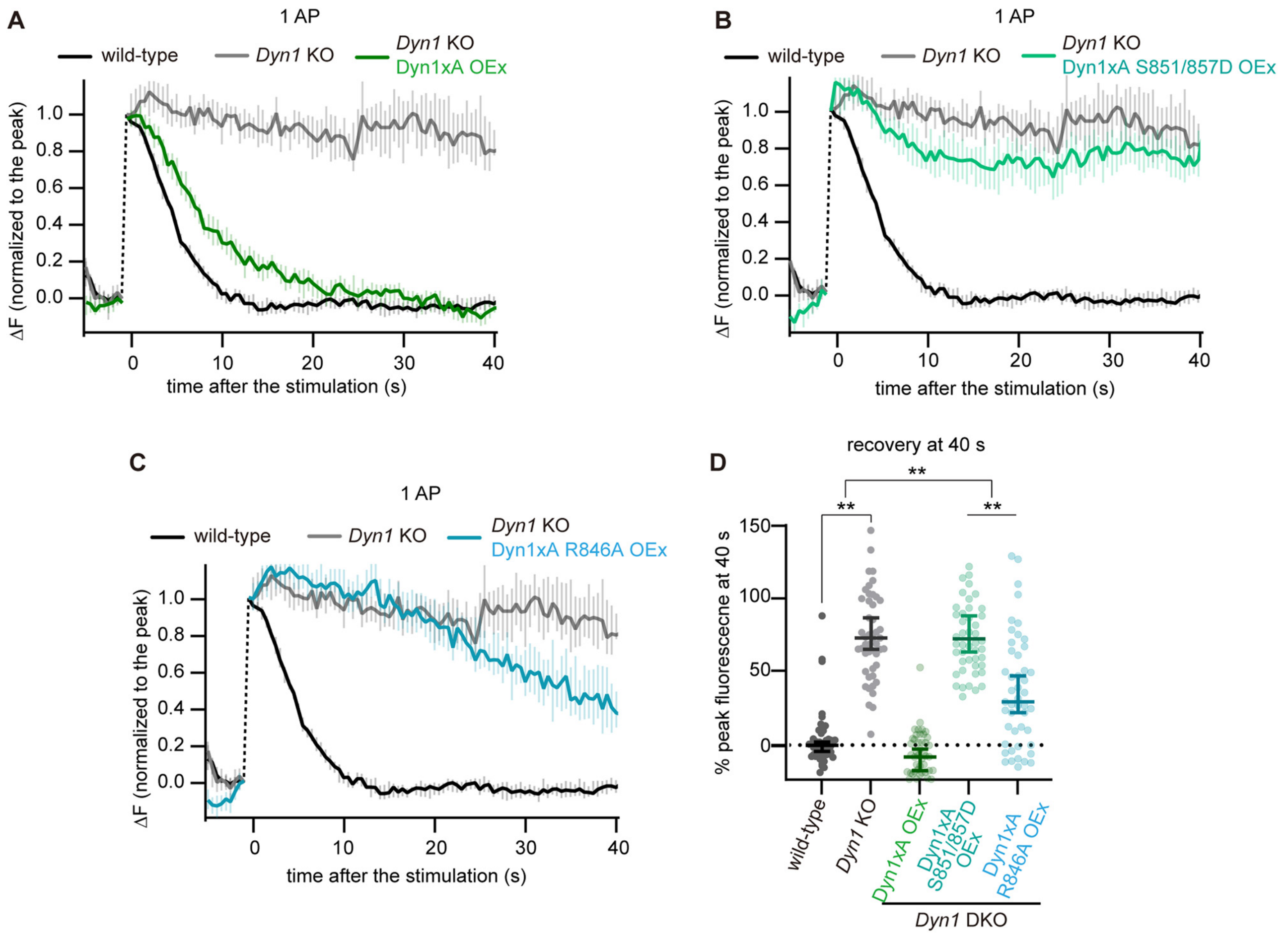

Figure EV5. Dyn1xA and its long tail is important for endocytosis of synaptic vesicle protein at single action potential.

(**A–C**) Plots showing average responses of vesicular glutamate transporter 1 (VGLUT1)-pHluorin in DNM1$^{+/+}$ (wild-type), DNM1$^{-/-}$ (*Dyn1* KO), *Dyn1* KO neurons, overexpressing Dyn1xA (*Dyn1KO* Dyn1xA OEx) (**A**), *Dyn1* KO neurons, overexpressing Dyn1xA S851/857D (*Dyn1KO* Dyn1xA S851/857D OEx) (**B**) or *Dyn1* KO neurons, overexpressing Dyn1xA R846A (*Dyn1KO* Dyn1xA R846A OEx). Mouse primary cultured hippocampal neurons were stimulated at single action potentials (AP). The fluorescence signals are normalized to the peak for each bouton. Before stimulation, fluorescence images are acquired for 5 s followed by the stimulation and continued acquisition. (**D**) The percentage of peak fluorescence remaining at 40 s after the beginning of the imaging. $n > 60$ presynaptic boutons from five different coverslips in each condition. $N = 2$ culture born from three different mothers at DIV14. $^{**}p < 0.0001$. Knock out neurons are from the littermates in all cases. Kruskal–Wallis Test with full comparisons by post hoc Dunn's multiple comparisons tests.
